# Parental genomes segregate into distinct blastomeres during multipolar zygotic divisions leading to mixoploid and chimeric blastocysts

**DOI:** 10.1186/s13059-022-02763-2

**Published:** 2022-10-03

**Authors:** Tine De Coster, Heleen Masset, Olga Tšuiko, Maaike Catteeuw, Yan Zhao, Nicolas Dierckxsens, Ainhoa Larreategui Aparicio, Eftychia Dimitriadou, Sophie Debrock, Karen Peeraer, Marta de Ruijter-Villani, Katrien Smits, Ann Van Soom, Joris Robert Vermeesch

**Affiliations:** 1 Laboratory for Cytogenetics and Genome Research, Department of Human Genetics, KU Leuven, 3000 Leuven, Belgium; 2 Reproductive Biology Unit, Department of Internal Medicine, Reproduction and Population Medicine, Ghent University, 9820 Merelbeke, Belgium; 3 Department of Clinical Sciences, Faculty of Veterinary Medicine, Utrecht University, 3584CM Utrecht, The Netherlands; 4 Hubrecht Institute, 3584CT Utrecht, The Netherlands; 5 Leuven University Fertility Center, University Hospitals of Leuven, 3000 Leuven, Belgium; 6 Division of Woman and Baby, Department Obstetrics and Gynaecology, University Medical Centre Utrecht, 3508 GA Utrecht, The Netherlands

**Keywords:** Zygote, Mitosis, Whole-genome segregation errors, Chromosomal instability, Triploidy, Chimerism, Mixoploidy, Mola, Multipolar division, Heterogoneic division

## Abstract

**Background:**

During normal zygotic division, two haploid parental genomes replicate, unite and segregate into two biparental diploid blastomeres.

**Results:**

Contrary to this fundamental biological tenet, we demonstrate here that parental genomes can segregate to distinct blastomeres during the zygotic division resulting in haploid or uniparental diploid and polyploid cells, a phenomenon coined heterogoneic division. By mapping the genomic landscape of 82 blastomeres from 25 bovine zygotes, we show that multipolar zygotic division is a tell-tale of whole-genome segregation errors. Based on the haplotypes and live-imaging of zygotic divisions, we demonstrate that various combinations of androgenetic, gynogenetic, diploid, and polyploid blastomeres arise via distinct parental genome segregation errors including the formation of additional paternal, private parental, or tripolar spindles, or by extrusion of paternal genomes. Hence, we provide evidence that private parental spindles, if failing to congress before anaphase, can lead to whole-genome segregation errors. In addition, anuclear blastomeres are common, indicating that cytokinesis can be uncoupled from karyokinesis. Dissociation of blastocyst-stage embryos further demonstrates that whole-genome segregation errors might lead to mixoploid or chimeric development in both human and cow. Yet, following multipolar zygotic division, fewer embryos reach the blastocyst stage and diploidization occurs frequently indicating that alternatively, blastomeres with genome-wide errors resulting from whole-genome segregation errors can be selected against or contribute to embryonic arrest.

**Conclusions:**

Heterogoneic zygotic division provides an overarching paradigm for the development of mixoploid and chimeric individuals and moles and can be an important cause of embryonic and fetal arrest following natural conception or IVF.

**Supplementary Information:**

The online version contains supplementary material available at 10.1186/s13059-022-02763-2.

## Background

A variety of errors occurring during initial mitotic divisions of the embryo following natural or in vitro fertilization (IVF) can lead to mosaicism, characterized by the co-existence of cell populations with a different genotype in a single individual [1–7]. It is well established that chromosomal mosaicism is notoriously common in human [8, 9] and bovine [10–12] early embryo development where it contributes to embryonic arrest, pregnancy loss [2, 13–22], or congenital disorders [23–27].

A peculiar form of mosaicism is characterized by the co-existence of cells with different genome ploidy or distinct parental genotypes in a single individual, identified as mixoploidy and chimerism, respectively. In particular, when the parental origin of one of these cell lineages is attributed to one parent only, this is called mosaic genome-wide (GW) uniparental disomy. Chimerism [28–32], mixoploidy [33–41], and mosaic GW uniparental disomy [42–48] have been shown to underlie rare developmental disorders in humans and cattle. They are also associated with defined clinical placental manifestations, such as placental mesenchymal dysplasia [49–51], and complete or partial hydatidiform moles [52, 53].

Although clearly associated with developmental disorders, the mechanistic origin of mixoploidy and chimerism remains elusive. Different models have been proposed to explain their origin, invoking (combinations of) zygotic and polar body aggregation, parthenogenetic activation, and fertilization errors. Some examples include the aggregation of two zygotes or a zygote and a fertilized polar body, the aggregation of a fertilized “empty egg” with a normal zygote and, the fusion of the second polar body or a sperm cell with one of the biparental diploid blastomeres at the two-cell stage [54–62]. However, these models are derived from studies performed on patients and abnormal placentae and rely on the cytogenetic or molecular genetic detection of cells that have undergone rigorous prenatal selection. Hence, these models remain speculative and largely unsupported by molecular or cell biological data.

With the development of concurrent GW single-cell haplotyping and copy number profiling [63, 64], we previously showed that androgenetic, gynogenetic, biparental, and triploid blastomeres can co-exist within individual day-2 and day-3 bovine embryos, both in vitro and in vivo [10, 11]. The existence of mixoploidy and chimerism was subsequently confirmed in other bovine [65], and non-human primate [66] in vitro-fertilized (IVF) cleavage-stage embryos.

As all cells from GW mosaic embryos originated from a single zygote, we hypothesized mixoploidy and chimerism to arise from the segregation of parental genomes into different daughter cells during the zygotic division. We coined this phenomenon “heterogoneic division” —Greek for a different parental origin— and reasoned that it might be enriched in embryos cleaving into more than two blastomeres directly (multipolar zygotic division) [10, 62]. Yet confirmation of this concept and its mechanism is lacking.

Here, we describe for the first time the existence of distinct parental cell lineages in human embryos. To test whether multipolar zygotic divisions coincide with parental genome segregation errors and gain insights in the mechanisms, we analyzed the GW haplotype architecture and ploidy state of all cells derived from in vitro-produced bovine zygotes undergoing multipolar division into three or four blastomeres. Moreover, to pinpoint how these mis-segregations arise, we followed the zygotic division, in real time, in bovine zygotes transiently expressing live fluorescent markers for chromosomes and microtubules. Together, these analyses prove that a multipolar zygotic division can result in the segregation of entire parental genomes into separate blastomeres and that resulting GW mosaicism can persist into the blastocyst stage.

## Results

### Genome-wide mosaicism exists in human blastocysts

**Fig. 1:**
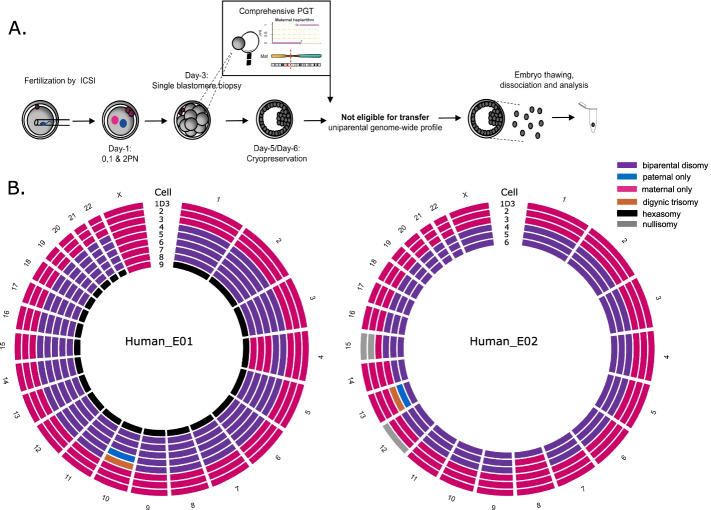
Human blastocysts characterized by GW mosaicism. **A** Experimental setup. ICSI: intracytoplasmic sperm injection; PN: pronuclei; PGT: preimplantation genetic testing. **B** Circos plots with each circle representing the genome constitution per chromosome of a single cell. The day-3 clinical biopsy (1D3) and cells 2 and 3 are gynogenetic. The remaining cells are biparental diploid or polyploid. Segmental chromosomal errors are not depicted

Considering that bovine and non-human primate embryogenesis parallel human embryogenesis, including the chromosomal instability [12, 66–70], we hypothesized mixoploidy and/or chimerism to be traceable in human cleavage- and blastocyst-stage embryos. In two human embryos, a gynogenetic blastomere at day-3 biopsy was identified through comprehensive haplotyping-based preimplantation genetic testing (PGT). Therefore, in order to understand if the detected GW aberrations were indicative of a mixoploid and/or chimeric embryo, we dissociated the resulting blastocysts for single-cell analysis (Fig. 1A). This analysis revealed a mixture of gynogenetic, biparental diploid, and polyploid cells (Fig. 1B). More specifically, comprehensive PGT showed a gynogenetic blastomere in both embryos, with human_E02 containing additional nullisomies. Upon dissociation of the blastocyst resulting from human_E01, two more gynogenetic cells in conjunction with six biparental cells were retrieved. In addition, one cell displayed an abundance of five identical copies of the maternal genome relative to paternal genome and, hence, represented a hexaploid cell. Analysis of five cells derived from blastocyst human_E02 showed the presence of two gynogenetic and three biparental diploid cells. As a result of mitotic segregation errors, (reciprocal) segmental or whole-chromosome losses and gains were detected in both the gynogenetic of one and biparental cells of both embryos. Taken together, these analyses point to the occurrence of human parental genome segregation errors and the persistence of these lineages to the blastocyst stage resulting into chimeric and/or mixoploid human blastocysts.

### Multipolar zygotic divisions are characterized by whole-genome segregation errors

**Fig. 2:**
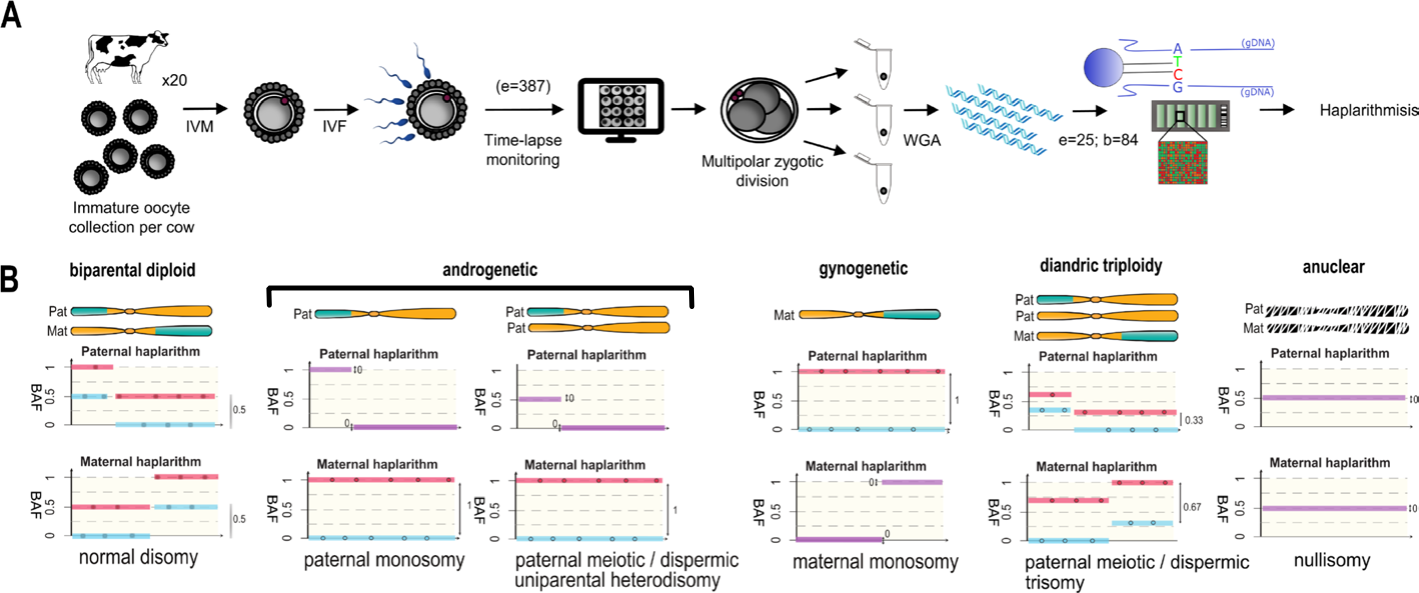
Study design and data analysis by haplarithmisis. **A** Study design. IVM: in vitro maturation; IVF: in vitro fertilization; WGA: whole-genome amplification; e=number of embryos; b=number of blastomeres and fragments. **B** Haplarithm patterns for a selection of genomic constitutions per chromosome (e.g., normal disomy, paternal monosomy, and paternal meiotic/dispermic uniparental heterodisomy (Full overview in Additional file 2: Fig. S2, A). Corresponding GW profiles (e.g., biparental diploid or androgenetic) are characterized by the manifestation of those patterns throughout the (majority of the) genome. During initial parental phasing, single-cell B-allele frequency (BAF) values are assigned to parental informative SNPs, rendering two paternal and two maternal subcategories (blue and red lines). Defined single-cell BAF values of the segmented subcategories in the sample form haplotype blocks, demarcated by pairwise breakpoints, i.e., homologous recombinations. Haplotype blocks, as well as the distance between the parental SNP subcategories in the paternal and maternal haplarithm, respectively, and the positioning of homologous recombinations, denote the origin and nature of copy number (more detailed explanation in Additional file 2: Fig. S2 and [64])

Because human embryo research is ethically and numerically restricted, we used a bovine model to pinpoint the origin of parental genome segregation errors. We combined the analysis of the genomic profiles of all blastomeres immediately following a multipolar zygotic division into three or four blastomeres (Fig. 2; Fig. 4A,B; Additional file 1: Movie S1, Additional file 2: Fig. S2) with the live-cell imaging of the zygotic division of zygotes transiently expressing live fluorescent markers for chromosomes and microtubules.

For analysis of genomic profiles and additionally, for staining purposes (see below), a total of 387 in vitro-produced bovine zygotes were recorded under time-lapse imaging until the zygotic division was observed. From all zygotes, 88.9% cleaved, of which 64.2% cleaved into two blastomeres and 35.8% cleaved into three or more blastomeres.

Haplarithmisis was subsequently performed in 82 single blastomeres and two cellular fragments obtained from a first set of 25 in vitro-produced bovine embryos. Embryos cleaved either into three blastomeres (*n*=16), three blastomeres with a fragment (*n*=2), or four blastomeres (*n*=7; Additional file 2: Fig. S2, B). Remarkably, in addition to (segmental) meiotic and mitotic chromosomal aneuploidies, all embryos (*n*=25) contained GW abnormalities in at least one blastomere, including polyploidy with additional maternal or paternal genomes, uniparental signatures (androgenetic or gynogenetic), the GW presence of complex aneuploidies, or the apparent absence of DNA. The separate segregation of whole parental genomes in different blastomeres (i.e., heterogoneic division) resulted in androgenetic and diploid or polyploid blastomeres or androgenetic and gynogenetic blastomeres in 18 embryos. Such an event occurred in 17 polyspermic and one monospermic embryos.

**Fig. 3:**
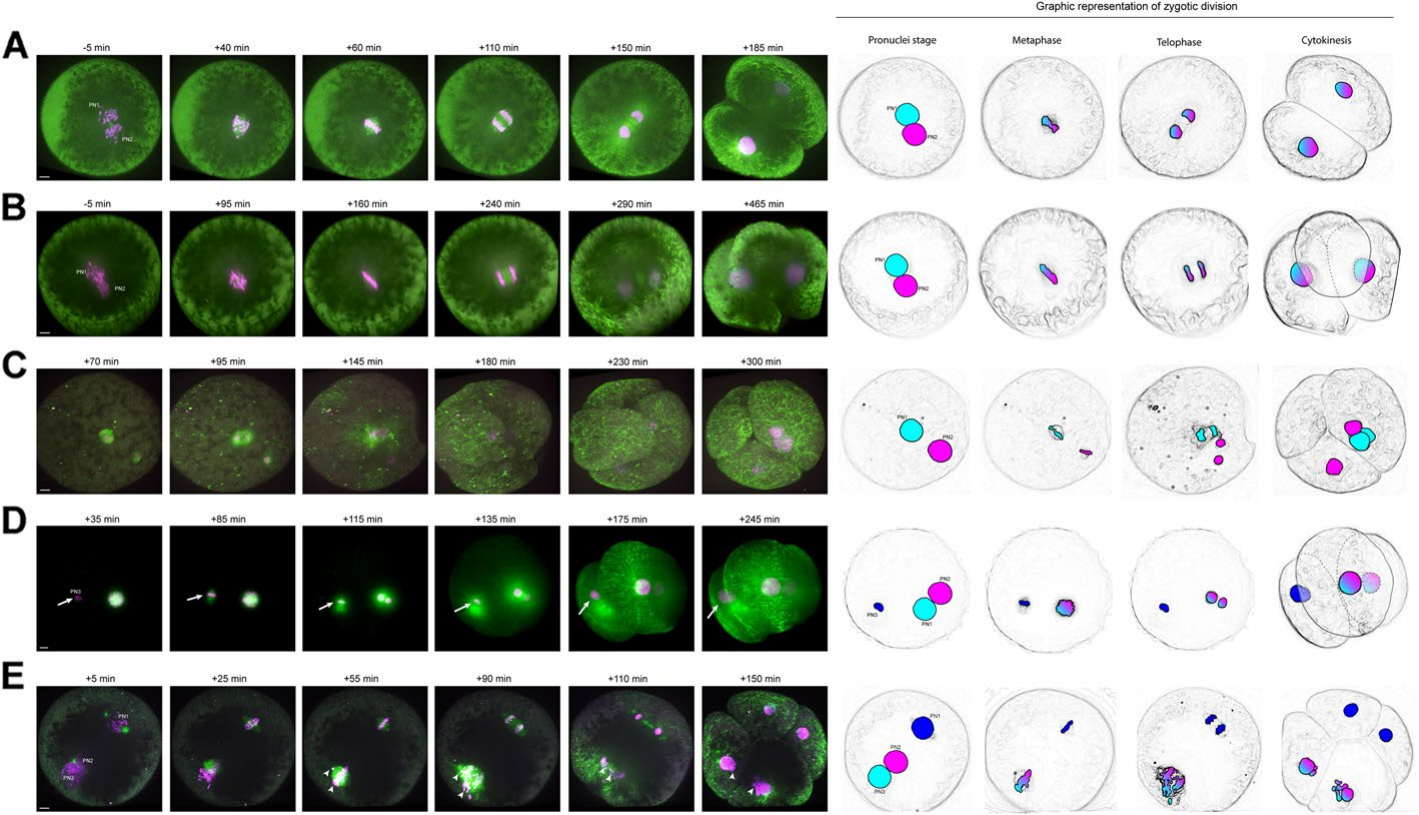
Spindle assembly and cytokinesis dynamics in live-imaged bovine zygotes. Spindle assembly and cytokinesis into two (**A**) or multiple blastomeres (**B–E**) in live-imaged bovine zygotes containing two (**A**, **C**) or three (**D**, **E**) pronuclei. **A–E** Bovine zygotes expressing transient microtubule markers (EGFP-MAP4, green) and chromatin marker (H2B-mCherry, magenta) were imaged by light sheet (**D**) or spinning disk (**A–C**, **D**) microscopy every 10 min throughout mitosis and for up to 12 h in total. Graphical representation of the zygotic division on the embryos imaged illustrating the segregation dynamics of the different genomes. Timings are respective to synchronous pro-nuclear evelope breadown (NEBD) (**A–C**) or to NEBD of the leading PN (PN1) in case of asynchrony (**D**, **E**). PN3, asynchronous lagging PN. Arrows indicate the extrusion of a parental genome into a separate blastomere. Arrowheads indicate misaligned and lagging chromosomes resulting in micronuclei formation in the daughter blastomeres. Projected scale bars, 10 μm

For the live-imaging, a total of 55 in vitro-produced bovine zygotes were injected with mRNA at the pronuclear stage to transiently express live fluorescent markers for chromosomes (H2B-mCherry) and microtubules (EGFP-MAP 4) [71]. The low-phototoxic imaging conditions, using either an inverted light sheet microscope or a spinning disk microscope, allowed bovine embryos to be imaged in 3D with a temporal resolution of 10 min throughout the zygotic division (bipolar division: Additional file 3: Movie S3_3A). Of the 55 imaged embryos, three showed a multipolar division in three cells and one in four cells (Fig. 3 and Additional file 4: Movie S4_3B; Additional file 5: Movie S5_3C; Additional file 6: Movie S6_3D; Additional file 7: Movie S7_3E). Two out of these four zygotes showed two pronuclei (Fig. 3B, C), while the other two showed three pronuclei (Fig. 3D, E). Both of the zygotes with only two pronuclei formed an anuclear blastomere after cytokinesis. In one of those (Fig. 3B), the parental genomes congressed into the same spindle and were separated into two mononucleated biparental cells. In the other (Fig. 3C), the parental genomes never congressed and formed two private parental spindles, which, after cleavage, gave rise to a mononucleated mono-parental cell and a multinucleated biparental cell containing an additional copy of the chromosomes from one of the parents. In one of the zygotes showing three pronuclei, one pronucleus was slower than the others in decondensing (Fig. 3D, PN3). This resulted in the asynchronous (50 min apart) formation of two separate spindles, one containing the genome of two pronuclei and the other, which formed later, one pronucleus of only one parent. While the first spindle underwent ana- and telo-phase and gave rise to two mononucleated biparental cells, the mono-parental spindle never proceeded to ana- or telo-phase and the additional parental genome was extruded in a mononucleated cell. The zygote which directly separated into four cells (Fig. 3D) also formed two separate spindles, one biparental and an additional mono-parental one. However, one of the pronuclei participating to the biparental spindle underwent nuclear envelope break down 20 min later than the first one. The lack of synchrony between the two pronuclei could be the cause of micronuclei formation upon cleavages, as the chromosomes might not have had enough time to align correctly onto the spindle before anaphase onset.

### Anuclear blastomeres can be formed following multipolar zygotic division

In 12 blastomeres and two cellular fragments, the haplarithm plot did not identify any GW haplotype despite a seemingly successful whole-genome amplification (WGA) (Additional file 2: Fig. S2; Additional file 8: Fig. S8). Given that blastomeres contained cell membranes upon visual inspection and had comparable sizes to the other blastomeres, we hypothesized that those cells might not contain nuclear DNA. Hence, we performed low-coverage whole-genome sequencing of the WGA product. For all twelve blastomeres and two cellular fragments, an abundance of mitochondrial DNA was observed (Additional file 8: Fig. S8, B). In nine blastomeres and two fragments, nuclear DNA was absent. In three blastomeres, a subset of the reads mapped back to some regions in the reference genome, consistent with signals seen on the haplarithm plot, which suggested the presence of chromosomal fragments (Additional file 8: Fig. S8, C; Additional file 2: Fig. S2). To exclude external DNA contamination, the mitochondrial sequences were reassembled and compared, demonstrating each of the sequences to be cow specific (Additional file 8: Fig. S8, A). Hence, the samples contained mitochondria but no complete nucleus and were categorized as “anuclear.” Overall, nine embryos (36%) carried at least one anuclear blastomere. This observation was confirmed by the live-imaging study, where we observed the formation of anuclear blastomeres in two out of the four zygotes undergoing multipolar division (Fig.3B,C, Additional file 4: Movie S4_3B; Additional file 5: Movie S5_3C).

To further confirm and estimate the incidence of anuclear blastomere formation and determine whether anuclear blastomeres can be observed also in bipolar zygotic cleavages, we stained nuclei with Hoechst for a total of 43 and 65 blastomeres, respectively isolated from a second set of embryos that underwent bipolar zygotic division (*n*=23) or multipolar zygotic division in three (*n*=10) or four (*n*=10) blastomeres. Following multipolar division of the zygote, nine anuclear blastomeres were observed in seven embryos (Additional file 8: Fig. S8, D). No anuclear blastomeres were observed after bipolar divisions.

### Polyspermic fertilization instigates multipolar division

**Fig. 4:**
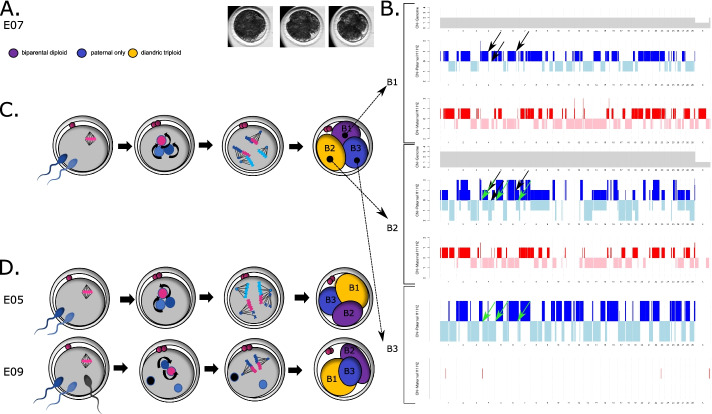
Haplarithmisis unveils GW mosaicism following multipolar zygotic division. **A** Three chronological time-lapse images of E07 (initiation of the cleavage furrow, the ongoing first division and the embryo immediately after cleavage and before cell isolation of the cleaving zygote). **B** A visual interpretation of the haplarithm profiles (Additional file 2: Fig. S2, B) for the biparental diploid (B1), the diandric triploid (B2), and the androgenetic blastomere (B3) of E07. For each blastomere from top to bottom, respectively, we depict the area and copy number inherited from paternal homolog 1 (H1, light blue) or paternal homolog 2 (H2, dark blue), together forming the paternal haplotype and the area and copy number inherited from maternal homolog 1 (H1, red) or maternal homolog 2 (H2, pink), together forming the maternal haplotype. Shifts from H1 to H2 represent recombination sites. Simultaneous presence of H1 and H2 represent regions of heterodisomy. In E07, a distinct recombination profile of the inherited paternal haplotype is retrieved in the biparental diploid (black arrows) and the androgenetic blastomere (green arrows) and a combination thereof is retrieved in the diandric triploid cell, pointing towards dispermic fertilization. Given the presence of a common paternal genome in B1 and B3, segregation from a common paternal pronucleus is implied, assuming one paternal copy to be present in B3. However, copy number is not represented in uniparental blastomeres since the value is normalized to a relative value of two in the absence of chromosomal errors or a second parental genotype. **C** Schematic representation of the suggested mechanistic origin of blastomeres in E07, including replication and pronuclear apposition of the parental genomes and karyokinesis by a tripolar spindle leading to a multipolar division of the zygote. **D** Schematic representation of possible events leading to the segregation of a zygote in a biparental diploid, a diandric triploid, and an androgenetic cell lineage in two other embryos. Curved arrows depict replication of the genome

Based on parental haplotype profiling, 21 embryos (84%) harbored paternal genomes with distinct haplotypes (Additional file 2: Fig. S2). In 18 of these embryos, blastomeres with distinct paternal haplotypes were indicative of polyspermic fertilization (Fig. 4B). In the remaining embryos (E08, E24, E25), all blastomeres contained identical GW paternal homologous recombination sites in combination with the GW presence of regions containing two non-identical paternal genotypes (i.e., heterodisomy, Fig. 2B). The latter made it impossible to distinguish fertilization by multiple sperm from fertilization by a single diploid sperm, caused by a paternal meiotic error (Fig. 2B; Additional file 2: Fig. S2 [14, 72]).

In contrast to the abundance of paternal genomes, an additional maternal genome was identified only in one embryo (E04). Based on haplotype profiling, the presence of the extra maternal genome was due to a meiotic error. Whole-genome maternal meiotic errors in three other embryos were also characterized by the loss of large segments (E16) or the complete loss of the maternal genome (E01 and E10) (Additional file 2: Fig. S2). This suggests that polyspermic fertilization is the main driver of multipolar division.

### Segregation errors occur via different mechanisms

**Fig. 5:**
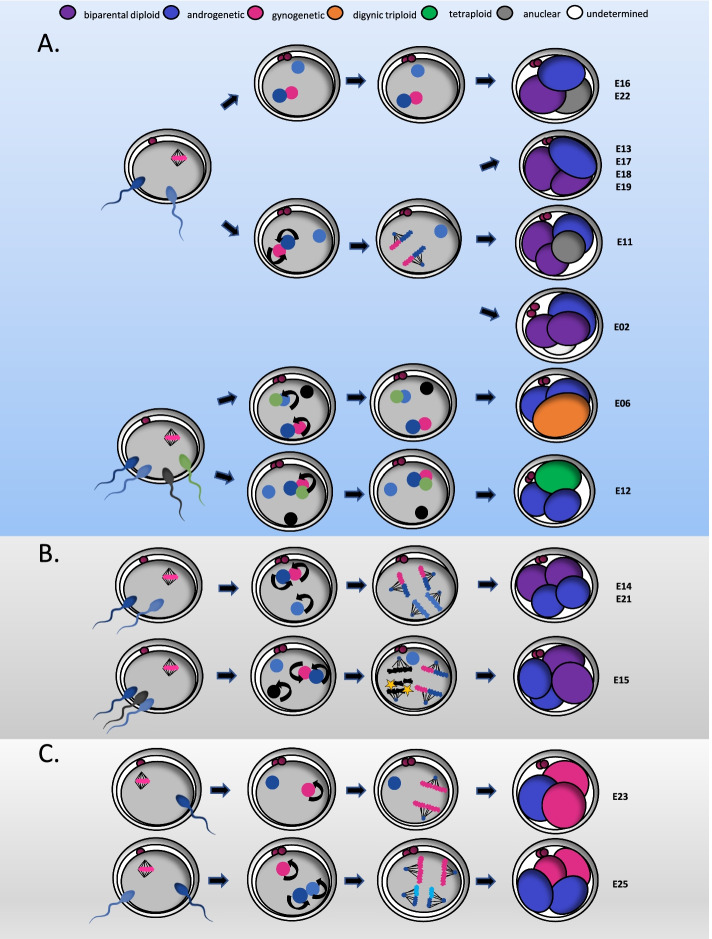
Schematic representation of events that best explain the segregation of a zygote into biparental and androgenetic (**A**, **B**) or androgenetic and gynogenetic blastomeres (**C**). Curved arrows depict replication of the genome. In **A**, extrusion of paternal genome(s) is depicted following polyspermic fertilization. In some embryos, this event occurred in parallel with the replication and karyokinesis of the primary maternal and paternal genome. In E06 and E12, some of the parental genomes were replicated but failed to undergo karyokinesis. In **B**, segregation of an additional paternal genome occurs through a second, paternally organized spindle. In E15, a third paternal genome was protruded to one of the androgenetic blastomeres and genome-wide chromosomal losses occurred on one paternal genome (yellow stars). In **C**, private parental spindles are established around the maternal and the paternal genomes (E25) following polyspermic fertilization, or only around the maternal genome following normal fertilization (E23). In E23, the paternal genome is extruded in a separate blastomere

We classified embryos in five categories, based on blastomere haplotype profiles (Fig. 2B; Fig. 4; Fig. 5; Additional file 2: Fig. S2). The first three categories were consistent with the segregation of a whole parental genome to a distinct cell lineage during the zygotic division, i.e., heterogoneic division. Within these categories, mapping of the segregational origin of the genomic content revealed distinct mechanisms to cause parental genome segregation during multipolar zygotic division. A fourth category of androgenetic embryos and a fifth group of polyploid embryos were consistent with whole-genome segregation errors other than heterogoneic division. Two embryos (E04 and E20) showed distinct profiles which were not categorized due to their complex nature (for details on those, see Additional file 2: Fig. S2, B).

#### Embryos with diandric triploid, biparental diploid, and androgenetic blastomeres

Three embryos (12%) consisted of an androgenetic, a biparental diploid, and a diandric triploid (i.e., containing two distinct paternal set of genes or haplotypes) blastomere. Despite the similar ploidy constitution, the molecular mechanisms preceding segregation were different (Fig. 4; Additional file 2: Fig. S2). Specifically, in two of these embryos (E05 and E07), two distinct paternal haplotypes were present, providing evidence that the oocyte was fertilized by two sperm cells. Both paternal haplotypes were present in the triploid blastomere and one of the other blastomeres (Fig. 4B; Additional file 2: Fig. S2). Hence, a tripolar spindle likely tethered the maternal and both paternal genomes during the zygotic division (Fig. 4C,D). In embryo E07, two copies of all three genomes were present. Therefore, replication of the maternal and both paternal genomes likely preceded the formation of one tripolar or multiple mitotic spindle(s) (Fig. 4B,C). In contrast, embryo E05 contained only one copy of one of the paternal genomes. Hence, one paternal pronucleus was not replicated before the onset of mitosis, but was stochastically incorporated into the hypodiploid and hypotriploid sister blastomeres, respectively, resulting in reciprocal paternal losses and gains for several chromosomes (Fig. 4D; Additional file 2: Fig. S2).

In E09, three distinct paternal haplotypes were present, indicating that the embryo was fertilized by three sperm cells. One of the paternal haplotypes was shared between the diandric triploid and the biparental diploid blastomere. A second and a third paternal haplotype were identified in the diandric triploid and androgenetic blastomere, respectively. Hence, one of the paternal pronuclei and the maternal pronucleus replicated and participated in the karyokinesis, while two other paternal genomes were extruded into the diandric triploid and androgenetic blastomeres (Fig. 4D; Additional file 2: Fig. S2).

#### Embryos with biparental and androgenetic blastomeres

Thirteen embryos (52%) consisted of a combination of biparental and androgenetic blastomeres (Fig. 5A,B; Additional file 2: Fig. S2). Multiple paternal haplotypes were detected in all embryos, indicating polyspermic conceptions. In three embryos, fertilization occurred by more than two sperm.

In embryos with two biparental diploid blastomeres, both paternal genomes were segregated via one spindle (Fig. 5A,B; Additional file 2: Fig. S2), as confirmed by the presence of the same maternal and paternal haplotypes and occasional reciprocal aneuploidies in both biparental blastomeres of some embryos (chromosome 8 in E13, chromosome 9 in E19 and complex in E21).

The androgenetic blastomere(s) in all 13 embryos presented one or more paternal haplotypes that were distinct from the haplotype observed in the biparental blastomere(s) (Additional file 2: Fig. S2). Hence, the extra paternal genomes were either extruded into a separate blastomere (Fig. 5A) or segregated by the operation of an additional paternal spindle (Fig. 5B). The latter was recognized by the presence of two androgenetic blastomeres containing the same paternal haplotype, indicating that a second spindle segregated the chromatids following genome replication (Additional file 2: Fig. S2). The operation of a second spindle was further confirmed by the presence of a segmental deletion of chromosome 3 in one and the reciprocal duplication in the other androgenetic blastomeres of E21 (Additional file 2: Fig. S2). The live-imaging experiment confirmed these last two observations (Fig. 3C,D; Additional file 5: Movie S5_3C; Additional file 6: Movie S6_3D) showing that, if the additional paternal spindle is formed contemporary to the biparental one, it will be able to segregate the replicated chromosomes, instead, if its formation is delayed, it will not be able to undergo ana- and telo-phase, and the additional paternal genome will be extruded into a separate blastomere. In some embryos, additional complexities were found (E06, E12, E15, E16, Fig. 5; for details on those see Additional file 2: Fig. S2).

#### Embryos with androgenetic and gynogenetic blastomeres

In two zygotes (8%), the parental genomes segregated into three or four blastomeres, each containing a maternal or paternal genome (Fig. 5C; Additional file 2: Fig. S2). In E25, regions of heterodisomy in both androgenetic blastomeres indicated fertilization by two haploid or a single diploid sperm followed by genome replication and karyokinesis by two private spindles. Each spindle segregated the paternal or maternal genome into two androgenetic or gynogenetic blastomeres (Fig. 5C; Additional file 2: Fig. S2). Embryo 23 contained only a single androgenetic and two gynogenetic blastomeres. Hence, the paternal genome was likely extruded into a separate blastomere without being segregated by a second spindle. In the live-imaging experiment, we also observed the formation of two private parental spindles (perpendicular to each other) in a zygote with two pronuclei (Fig. 3C, Additional file 5: Movie S5_3C). One spindle segregated the parental genome in two separate blastomeres, giving rise to a mono-parental blastomere; however, the remaining spindle failed to induce a correct cytokinesis, giving rise to an anuclear blastomere and a biparental blastomere containing two copies the genome of one parent.

#### Androgenetic embryos

In two embryos, the blastomeres did not contain a maternal genome. The zygotes cleaved in three or four blastomeres consisting of two anuclear and one or two androgenetic blastomeres, respectively (Additional file 2: Fig. S2). Both androgenetic blastomeres in E10 contained an identical haplotype. Hence, they resulted from genomic replication of the paternal genome followed by karyokinesis. In E01, a single androgenetic and two anuclear blastomeres were observed which pointed towards the extrusion of the paternal genome into a blastomere without active segregation by a spindle.

#### Polyploid embryos

Two embryos (8%) consisted of two polyploid and one anuclear blastomere (Additional file 2: Fig. S2). The polyploid blastomeres were diandric triploid (E24) and tetra-andric pentaploid or triandric tetraploid (E03). Regions of paternal heterodisomy indicated polyspermy in both embryos. Based on genomic profiles that were identical (E24) or semi-identical containing multiple reciprocal aneuploidies (E03), the segregation of all genomes likely occurred by a single bipolar spindle (for more details on the segregation of E03 see Additional file 2: Fig. S2).

One embryo (E08) consisted of a triandric tetraploid blastomere and two anuclear blastomeres (Additional file 2: Fig. S2). Regions of heterodisomy disclosed the presence of three distinct paternal haplotypes in the tetraploid blastomere. Therefore, fertilization occurred by three sperms, followed by cytokinesis and either asynchronous karyokinesis or no karyokinesis at all.

### Genome-wide abnormalities resulting from heterogoneic division contribute to embryonic arrest and persist in the blastocyst-stage bovine embryo

**Fig. 6:**
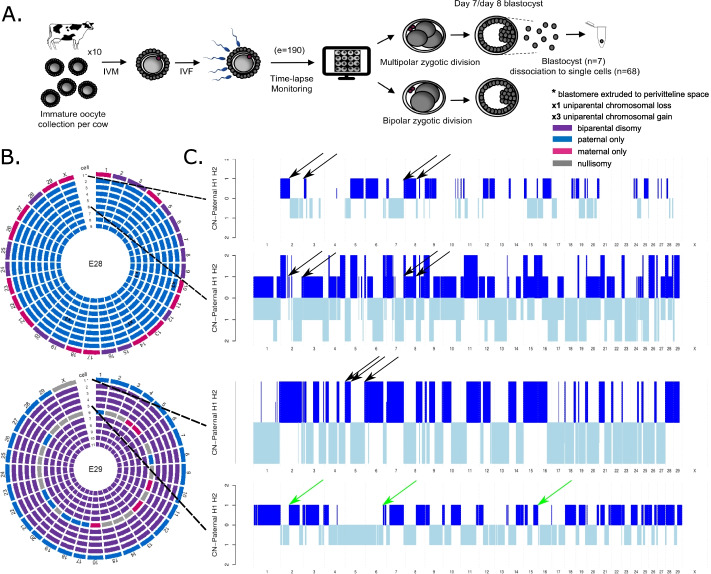
Genome-wide mosaicism persists to the bovine blastocyst stage (Full overview in Additional file 9: Fig. S9). **A** Experimental setup. IVM: in vitro maturation; IVF: in vitro fertilization. **B** Circos plots of two bovine blastocysts in which each circle represents the genome constitution per chromosome of a single cell. In E28, cell 1 presented with alternating biparental disomic and maternal chromosomes while the remaining cells were androgenetic. In E29, cell 1 was found to be androgenetic while cells 2–5 and 7–11 were biparental diploid. Cell 6 displayed alternating paternal chromosomes, maternal chromosomes, or nullisomies. **C** A visual interpretation of the paternal haplarithm profiles (Additional file 9: Fig. S9) of two cells from each embryo. For each blastomere from top to bottom, respectively, we depict the area and copy number inherited from paternal homolog 1 (H1, light blue) or paternal homolog 2 (H2, dark blue), together forming the paternal haplotype. Shifts from H1 to H2 represent recombination sites. Simultaneous presence of H1 and H2 represent regions of heterodisomy. In E28, identical homologous recombination sites were retrieved in the androgenetic cells and biparental disomic regions of cell 1 (black arrows). Heterodisomic regions throughout the genome of cells 2–9 (Pat-BAF = 0.5) revealed the presence of a second paternal haplotype. In E29, the androgenetic and diploid cells present with different paternal homologous recombination sites (green arrows). Copy number is not represented in the androgenetic cell 1 of E29 since the value is normalized to a relative value of two in the absence of chromosomal errors or a second parental genotype. Segmental chromosomal errors are not depicted

To prove that blastomeres containing GW abnormalities can propagate and persist following heterogoneic division, we cultured an additional cohort of bovine embryos to the blastocyst stage. Time-lapse cinematic information, acquired from a total of 190 zygotes, showed that 92.6% of zygotes cleaved and 41.5% developed to the blastocyst stage. From zygotes that underwent cleavage, 64.8% divided into two blastomeres and 35.2% divided directly into three or more blastomeres. The blastocyst rate from bovine zygotes that underwent multipolar division (30.1 ± 8.1%) was reduced compared to zygotes that underwent bipolar division (49.3 ± 7.1%) (*P* = 0.03), which shows that embryos with GW abnormalities resulting from multipolar zygotic division are more prone to embryonic arrest. Seven blastocysts which formed from zygotes that underwent multipolar zygotic division were dissociated and six to twelve cells were genotyped individually (Fig. 6A, Additional file 9: Fig. S9).

The subgroup of sampled cells presented a biparental diploid constitution in two blastocysts (E26, E31) and a GW mosaic constitution in five blastocysts (E27 to E30, E32). Remarkably, all cells located in the perivitelline space contained a GW abnormal profile while five out seven blastocysts contained almost exclusively biparental diploid cells in the embryonic mass. In addition, a number of meiotic (E26) and (reciprocal) (segmental) mitotic aneuploidies (E28, E30, E31) were retrieved and three embryos contained a cell with complex chromosomal aneuploidies (E28, E29, E32). In the five GW mosaic embryos, cells of the same blastocyst showed distinct paternal haplotypes or GW paternal heterodisomy, confirming polyspermy to instigate multipolar zygotic division (Fig. 6C). Specifically, three of the GW mosaic blastocysts contained biparental diploid cells in the embryonic mass and one or two androgenetic cells of a distinct paternal haplotype extruded in the perivitelline space (E27, E29, E30) (Fig. 6B,C). In addition, one cell of E29 presented with complex chromosomal losses, resulting in a fragmented genome containing (segmental) androgenetic and gynogenetic chromosomes and nullisomies. The fourth GW mosaic blastocyst (E28) contained diandric androgenetic cells with GW regions of heterodisomy in the embryonic mass and one biparental diploid cell with complex (segmental) paternal losses extruded in the perivitelline space (Fig. 6B,C). The last GW mosaic blastocyst (E32) contained one triandric tetraploid cell with complex paternal losses, one tetraploid cell and three triploid cells, all containing GW regions of heterodisomy.

## Discussion

In this study, we used a bovine model to demonstrate that heterogoneic division (i.e., the segregation of parental genomes into different blastomeres during the first embryonic division) is common in embryos that undergo multipolar zygotic division and plays an important role in the origin of GW mosaicism in blastocysts. By reconstructing the segregational origin of the parental haplotypes in the daughter blastomeres and by live-imaging experiments, we show that heterogoneic cell division can be caused by distinct mechanisms. In addition, we provide the first direct evidence of the existence of distinct parental cell lineages in human embryos.

### Mixoploidy and chimerism exist in human and bovine embryos and are caused by heterogoneic division of the zygote

Single-cell genotyping and haplotyping has shown that chromosomal instability is extremely frequent during the first cleavages of human, bovine, and primate embryos. This instability is the cause of the high incidence of chromosomal aneuploidy and chromosomal mosaicism in cleavage-stage embryos and blastocysts [8–11, 66, 73]. To select for euploid embryos, which have a higher implantation and baby-take home rate as compared to aneuploid embryos, preimplantation genetic testing for aneuploidies (PGT-A) has become a standard procedure in many human IVF laboratories world-wide. However, most PGT-A methods only interrogate chromosomal copy number changes and do not determine the haplotypes [73–75]. Thanks to the implementation of single-cell haplotyping methods in PGT, the existence of androgenetic, gynogenetic, and triploid embryo biopsies has been uncovered [14, 73, 76–78]. Only by using this method on all cells of cleavage-stage embryos, the existence of mixoploidy and chimerism was uncovered in in vitro and in vivo-derived bovine embryos and in in vitro-derived primate embryos [10, 11, 65, 66]. Here, we demonstrate for the first time directly that mixoploidy and chimerism are also present in human blastocysts.

By tracking division kinetics and determining the genomic structure of all single blastomeres following multipolar zygotic division in a bovine model, we further demonstrate that parental genomes can segregate independently into different blastomeres, resulting in distinct combinations of co-existing biparental, polyploid, and uniparental blastomeres. Cytogenetic evaluation of individual cells in cleavage-stage and blastocyst-stage human embryos in the previous century already reported the co-existence of diploid and haploid or polyploid cells, both following presumed polyspermic fertilization [79–87] or normal fertilization [88–90] and some suggested this to result from the zygotic division [80, 85, 87]. More recently, our group hypothesized bovine mixoploid and chimeric cleavage-stage embryos to be the consequence of parental genome segregation into different blastomeres during the zygotic division, an event we coined the heterogoneic zygotic division [10, 62]. However, former studies were hampered by the lack of genome-wide genetic analysis, the inability to track cytokinetic events or did not analyze all cells of the embryo. Hence, we here provide the first direct evidence for a heterogoneic zygotic division to cause mixoploidy and chimerism. This finding contradicts a most fundamental biological paradigm in early development stating that during the first embryonic—i.e., zygotic—division the replicated and juxtaposed paternal and maternal genomes segregate one copy of each parental genome into two biparental diploid daughter cells [91]. We further demonstrate that resulting GW aberrations are able to persist throughout the preimplantation period forming mixoploid and chimeric blastocysts.

Multipolar zygotic divisions, which coincide with heterogoneic division in the majority of bovine embryos, occur frequently (8.3–26%) in human embryos too [3]. In human, the multipolar zygotic division has been associated with hypodiploid blastomeres and variants of the Polo-like kinase 4 (PLK4) gene [1, 92]. Since PLK4 is a centrosome regulator, tripolar spindles generated by centrosomal dysfunction or dysregulation are thought to randomly segregate the diploid chromosome complement in hypodiploid blastomeres, which are characterized by excessive chromosomal losses rather than the segregation of whole parental genomes through heterogoneic division [1, 2, 73]. The majority of GW mosaic bovine embryos described here contain two different paternal haplotypes indicating the oocyte was fertilized by (at least) two sperm, pointing to polyspermic fertilization as a major trigger of multipolar and heterogoneic division of the zygote. Polyspermy occurs at similar frequencies for human (6–32% [93, 94];) and bovine (15–35% [95, 96];) in in vitro-fertilized zygotes. Yet, in human reproductive medicine, polyspermy is avoided by the use of ICSI (76% of 2019 cycles in Belgium) and the selection against zygotes presenting with more than two pronuclei at day 1. This selection might in its turn avoid most cases of heterogoneic division following polyspermy in human in vitro-produced embryos. In natural conception, however, heterogoneic division caused by polyspermy might occur frequently. Indeed, triploidy, one of the most prevalent causes for early miscarriage, occurs approximately in 1–2% of all pregnancies and is mostly being caused by the fertilization by two sperm cells [97–99]. On the other hand, polyspermic fertilization is no prerequisite for heterogoneic division as shown by the ICSI-derived human blastocysts and a smaller subset of bovine embryos in the current study that were fertilized by one sperm. In addition, a recent study also visualized the segregation of a parental genome to one of the blastomeres following monospermic fertilization and bipolar zygotic division [71]. Therefore, it seems likely that normally fertilized, diploid zygotes could either result in hypodiploid embryos following multipolar division or, in GW mosaic embryos following multipolar or bipolar heterogoneic division. The true incidence of mixoploidy and chimerism in in vitro and in vivo human embryogenesis will require systematic studies where all or at least a representative number of individual blastomeres of embryos of both normally and abnormally fertilized embryos are haplotyped, following both bipolar and multipolar zygotic division.

### Heterogoneic division occurs via a multitude of pathways

Haplotypes and the live-imaging experiments provide indirect evidence for tripolar spindle formation and both indirect and direct (live-imaging) evidence for additional paternal and private parental spindles as well as for the extrusion of paternal genomes. The failure to capture a tripolar or tetrapolar spindle by live-cell imaging might be caused by the fair low number of embryo analyzed in the live-imaging experiments.

For several embryos, it is indicated that often not one but more spindles segregated the genomes into three or four blastomeres (Fig. 4C,D, E05; Fig. 5B,C; Additional file 2: Fig. S2). This is in accordance with our previous experiment showing that in bovine zygotes two parental spindles form independently around each pronucleus, generating two rather than a single bipolar spindle, in a monospermic zygote [71, 100]. In a normal zygotic division, these so-called “dual spindles” align and fuse during early metaphase, resulting in a single bipolar spindle before genome segregation. However, faulty alignment of one of the spindle poles would result in the independent segregation of a whole parental genome. Indeed, experimental induction of spindle non-alignment in mice gave rise to segregation of parental genomes in different directions leading to gross mitotic aberrations (e.g., formation of binucleated blastomeres or multipolar division) [100]. In addition, experimental non-unification of parental genomes led to formation of private parental spindles, each segregating only one of the parental genomes [101]. The physiological occurrence of tripolar spindles is further evidenced by their visualization in human 3PN zygotes [81, 102]. Also additional paternal and private parental spindles have been visualized as additional metaphases and spindles in 3PN bovine, human, and rhesus monkey zygotes [83, 87, 102–107] and as co-existing maternal meiotic and a sperm-derived mitotic-like spindles in presumed normally fertilized zygotes by Van Blerkom and colleagues in so-called “silent fertilizations,” i.e., fertilizations in which no pronuclei are visualized in the zygote [108].

In most embryos, we demonstrate that additional paternal genomes were not segregated by a spindle but were extruded to one or more distinct cell lineages, resulting in androgenetic and polyploid blastomeres (Fig. 4D, E09; Fig. 5A; Additional file 2: Fig. S2). This observation endorses early cytogenetic studies on human 3PN embryos which predicted the exclusion of one haploid genome from the metaphase plate of the first division, resulting in 2n, 2n/3n mosaics and 1n/2n derivatives [79–87]. Based on cytogenetic analysis after the first cleavage [80, 87] of human 3PN zygotes, such an exclusion was suggested to occur by an extrusion of a haploid genome, i.e., a pseudo-cleavage in which cytokinesis occurs while the genome is still packed inside the sperm head or in the pronuclear stage. We visualized such extrusion of a parental genome in one of the bovine embryos used for the live-imaging experiments (Fig. 3D). It remains unknown which molecular mechanisms might trigger the extrusion of a genome.

### Anuclear blastomeres

Unexpectedly, we observed a high incidence of anuclear blastomeres, both in the genomic and in the live-imaging studies. Anuclear blastomeres formed at cytokinesis in absence of a concurrent genome segregation. This finding is in accordance with previous studies which detected anuclear blastomeres in cleavage-stage embryos of different species [66, 109–113] and further shows their origin to be associated with multipolar zygotic division. The molecular mechanisms leading to anuclear blastomere formation remain unknown. Some suggested that dissociation of the karyokinesis from the cytokinesis, due to altered spindle dynamics or altered actin polymerization/depolymerization related to in vitro maturation, could underlie anuclear blastomere formation [109, 111]. However, direct evidence is lacking. Alternatively, we suggest that centrosomes may be involved in the segregation of anuclear blastomeres as it has been shown that centrosomes/centrioles that can prematurely detach from the sperm head [114, 115], dissociate and migrate away from the spindle in the cytoplasm inducing more often in abnormal cleavage kinetics [71] and are able to initiate cytokinesis independently [116].

### Heterogoneic division provides an alternative but overarching explanation for the origin of molar pregnancies and mixoploid and chimeric individuals

**Fig. 7:**
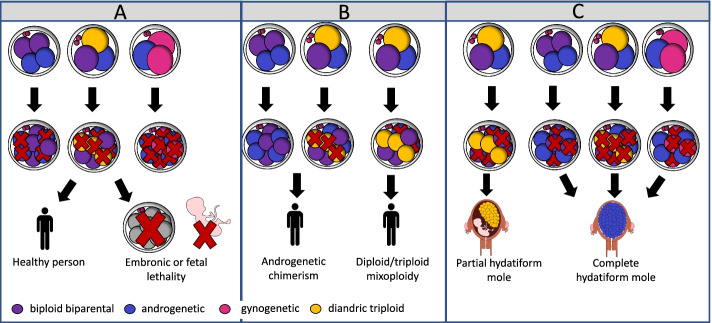
Model explaining the origin of mixoploidy in **A** healthy individuals or cases of developmental arrest **B** rare chimeric individuals and **C** clinical placental outgrowths. Following heterogoneic zygotic division, the different blastomeres of the mosaic embryos may develop differently

Outgrowth or survival of one or more of the blastomeres following heterogoneic division likely contributes to low human fecundity and provides an overarching explanation for the persistence of GW anomalies in fetuses and patients (Fig. 7) [62]. Our time-lapse data reveal an impaired embryonic development rate from bovine zygotes that present with a multipolar division, confirming other studies which in addition found reduced implantation and pregnancy rates in both human [3, 117–121] and cattle [122]. Following heterogoneic division, androgenetic, gynogenetic, biparental, and triploid blastomeres could have a different evolution: some might propagate better than others and perhaps, undergo a selective advantage. As both maternal and paternal transcripts make an important contribution to preimplantation development [123, 124], it can be assumed that polyploid, haploid, and uniparental diploid blastomeres, resulting from whole-genome segregation errors, have a selective disadvantage compared to normal, biparental diploid blastomeres. Similarly to the complex aneuploid embryos, GW mosaic embryo constitutions may therefore cause developmental arrest by an insufficient number or lack of diploid cells and the occurrence of gene dosage imbalances following genome activation [2, 14, 125] (Fig. 7A). On the other hand, compensatory proliferation of the biparental diploid blastomere and/or active selection against the polyploid, haploid, or uniparental diploid cells could result in the progressive depletion of blastomeres with whole-genome errors and ensure a sufficient number of viable blastomeres [125, 126] (Fig. 7A). We demonstrate that the bovine and human blastocysts can contain different genomic constitutions. Similar to what has been observed for complex aneuploid cells [3, 66, 73], several bovine blastocysts contained large complex aneuploid or androgenetic blastomeres in the perivitelline space, in which a developmental block seems to prevent those from getting incorporated in the morula and blastocyst stage. Nonetheless, some human and bovine embryos did retain the GW mosaic or androgenetic embryo constitution up to the blastocyst stage. We hypothesize that, occasionally, polyploid, haploid, and uniparental diploid cells are viable and further develop into rare pre- and post-natal and placental aberrations dependent on the selective pressures at play. These include chimeric and/or mixoploid, androgenetic and triploid, cell populations, occasionally observed in patients with developmental disorders, sesquizygotic twins [127] and molas (Fig. 7B,C).

## Conclusions

In conclusion, this study generated direct proof of heterogoneic division in mono- and polyspermic bovine zygotes and showed that different hitherto unknown molecular mechanisms form the basis of a variety of chimeric and mixoploid embryos. In addition, some GW segregation errors were found to persist to the human and bovine blastocyst stage. These findings demonstrate that failure of the parental genome congression on a single spindle and the asynchrony with which different pronuclei undergo the cell cycle have an important role in inducing GW mis-segregations in zygotes. Moreover, they raise questions about the mechanisms triggering zygotic cell division, zygotic spindle formation, and genome replication. By combining novel live-imaging technologies with single-cell genome and transcriptome analysis of embryos, it will become possible to unravel the cascade of cellular and molecular events involved in the meiotic to mitotic conversion of the zygote and deviations from the normal process that result in whole-genome segregation errors. Since most current PGT technologies overlook GW segregation errors, it seems likely such errors are a hidden cause of embryonic arrest and systematic surveying IVF embryos for such errors, including embryos containing three pronuclei that are usually discarded, may further improve embryo selection, increasing implantation and baby-take home rates.

## Methods

### Bovine embryo in vitro production (IVP)

#### Media and reagents

Basic Eagle’s medium amino acids, Minimal Essential Medium non-essential amino acids, TCM-199-medium, Ca^+2^/Mg^+2^-free PBS, kanamycin, and gentamycin were purchased from Life Technologies Europe (Merelbeke, Belgium) and all other components were obtained from Sigma-Aldrich (Schnelldorf, Germany), unless otherwise stated. All the media were filter-sterilized using a 0.22-μm filter (GE Healthcare, Chicago, USA) before use.

#### Procedure

Bovine embryos were produced by routine in vitro methods [128]. Briefly, bovine ovaries (*Bos taurus*) were collected at the local slaughterhouse and processed within 2 h. The ovaries were washed three times in warm physiological saline solution supplemented with kanamycin (25 mg/mL). Follicles between 2- and 8-mm diameter were punctured with an 18-G needle and a 10-mL syringe in 2.5 mL of Hepes-Tyrode’s albumin-pyruvate-lactate (Hepes-TALP) and kept separate per ovary. Cumulus-oocyte-complexes were collected using a stereomicroscope, washed in Hepes-TALP, and washed in maturation medium, which consisted of modified bicarbonate-buffered TCM-199 supplemented with 50 μg/mL gentamycin and 20 ng/mL epidermal growth factor. Subsequently, maturation occurred per donor in 500 μL maturation medium in four-well plates (Nunc^TM^) for 22 h at 38.5°C in 5% CO_2_ in humidified air. For fertilization, frozen-thawed semen of three different Holstein-Friesian bulls (*Bos taurus*) at a concentration of 1 × 10^6^ spermatozoa/mL was used (one single bull per replicate) following separation of spermatozoa over a discontinuous Percoll gradient (45% and 90%; (GE Healthcare, Chicago, US)). Fertilization was achieved by incubating the matured oocytes with spermatozoa for 21 h at 38.5 °C in 5% CO_2_ in humidified air. Specifically, E01–E20 were fertilized with semen of bull A, E21–E25 were fertilized with semen of bull B, and E26–E32 were fertilized with semen of bull C. The zygotes used for mRNA injection and live-imaging of DNA and microtubules during the zygotic division were fertilized using bulls B and C. The presumptive zygotes were transferred to synthetic oviductal fluid (SOF) supplemented with essential and non-essential amino acids (SOFaa), 0.4% BSA, and ITS (5 μg/mL insulin, 5 μg/mL transferrin, and 5 ng/mL selenium) after removal of excess spermatozoa and cumulus cells by a 3-min vortex step.

### Time-lapse imaging system

One or two Primo Vision^TM^ micro well group culture dishes (Vitrolife, Göteborg, Sweden) per donor cow were used and consist of 9 or 16 small wells covered by a 40-μL droplet of medium and 3.5 mL mineral oil to prevent evaporation. The dishes were placed into the sample holder of a compact, digital inverted microscope (Primo VisionTM; Vitrolife, Göteborg, Sweden) which was placed in the incubator. Presumed zygotes were incubated at 38.5°C for up to 8 days in a trigas incubator (5% CO_2_, 5% O_2_, and 90% N_2_). The focus was set mechanically, and all embryos were positioned in the microscopic field-of-view. Every 5–10 min, a single picture was taken. Using time-lapse imaging and TeamViewer (Microsoft, Washington, USA), embryos were monitored from a distance to allow immediate processing of embryos that showed a direct cleavage of the zygote into three or four blastomeres (multipolar division) or into two blastomeres (bipolar division) (Additional file 1: Movie S1). All images recorded were saved upon analysis of the cleavage kinetics later by the Primo Vision Analyzer Software.

From embryos cleaving in three or more blastomeres [121], those that cleaved into more than four cells and those displaying multiple fragments, obvious larger and smaller blastomeres, or cytokinetic aberrations other than multipolar division (e.g. “membrane ruffling” or “reverse cleavages”) were not considered for analysis. In addition, we did not collect blastomeres from embryos that went through the second or third division round (e.g., during the night, or because of two rapid serial cleavages) following a first multipolar zygotic division.

### Live-imaging of bovine embryos

#### Expression constructs and mRNA synthesis

Synthesis of mRNA was performed using constructs previously described (pGEMHE-H2B-mCherry, [129]; pGEMHE-EGFP-MAP4, [130], purchased via Euroscarf (Oberursel, Germany). Capped and poly-adenylated mRNA was synthesized in vitro from linearized template DNA (1 μg) using the mMESSAGE mMACHINE T7 ULTRA Transcription kit (AM1345; Thermo Fisher Scientific). After purification (74104, RNeasy Mini Kit; QIAGEN), the mRNA was dissolved in 14 μL RNase-free water.

#### mRNA injection and live-imaging of DNA and microtubules in bovine zygotes

Fertilized zygotes were selected from unfertilized oocytes through scoring for two polar bodies. Cow zygotes were then injected with mRNA as previously described [71] with some modifications. In brief, injection of mRNA was performed using an inverted microscope (Olympus IX71) equipped with a micromanipulator (Transfer Man NK2; Eppendorf). Zygotes were held stationary by suction via the holding pipette (MPH-LG-Angled 308; Origio) with the polar body positioned at 12 o’clock and the injection needle (MIC-50-Angled 308; Origio) advanced through the zona pellucida and plasma membrane at the 3 o’clock position for the mRNA injection. The mRNA concentrations injected were 0.3–0.5 μg/μL for H2B-mCherry and 0.6–0.8 μg/μL for MAP4-EGFP in an injection volume of 4–5 pL (~0.5% of the bovine zygote volume).

#### Live-imaging of bovine zygotes

Live-imaging of bovine zygotes was performed either using a spinning disk microscope or an inverted light sheet microscope. For both systems, bovine zygotes were immobilized by embedding them in a gel mix composed by one third of Matrigel (Corning, 356231) and two third of transparent SOF. Zygote images with the Nikon TiE-based CSU-W1 Spinning Disk (equipped with a 1.20 NA Plan Apo VC 60XWI objective and an Andor iXon EMCCD camera) were mounted on an 8 Well IBIDI μ-Slide with glass Bottom (IBIDI, 80807) and embedded in 40 μL gel drops, the drops were covered with 300 μL transparent SOF and further covered 100 μL of mineral oil (Ovoil, Vitrolife, 10029) to prevent evaporation. Zygotes imaged with the inverted light sheet microscope (Luxendo InVi SPIM equipped with a Nikon CFI Apo 25XWI/1.1 NA water immersion detective objective, a Nikon 10×/0.3 NA water immersion illumination objective, a CMOS Hamamatsu, ORCA Flash4.0 V2 camera with line-scan mode in LuxControl) were mounted on a V-shaped sample holder covered with transparent FEP as previously described [71] and embedded in 20-μL gel drops, covered with 130 μL transparent SOF and further covered 150 μL of mineral oil (Ovoil, Vitrolife, 10029) to prevent evaporation. The illumination plane and focal plane were aligned before each imaging session and maintained during the light sheet imaging. In both systems, the sample holder was enclosed in an environmentally controlled incubation box with 5% CO_2_ and 5% O_2_ at 38.5 °C. For imaging of chromatin and microtubule lattice, fluorescence from H2B-mCherry and EGFP-MAP4 was acquired every 10 min using a 488-nM laser and a 561-nM laser. Stacks of 100–104 μm were acquired with a z-step size of 1–1.04 μm.

### Bovine genetic analysis

#### Single-cell isolation and whole-genome amplification

For blastomere isolation following the first division, embryos were washed in warm TCM-199 with 10% fetal bovine serum (FBS) immediately upon the visualization of a multipolar or bipolar division. Thereafter, embryos were treated with pronase to dissolve the zona pellucida (0.1% protease from *S. griseus*, in TCM-199) and subsequently washed in TCM-199 with 10% FBS followed by Ca^+2^/Mg^+2^-free PBS with 0.05% BSA to stimulate blastomere dissociation. Next, embryos were transferred to Ca^+2^/Mg^+2^-free PBS with 0.1% polyvinylpyrrolidone (PVP) for blastomere dissociation with a STRIPPER pipet holder and a 135-μm capillary (Origio, Cooper Surgical, CT, US). When characterized by a small diameter, irregular shape and absence of a clear cell membrane a blastomere was marked as a fragment [131, 132]. For isolation of single cells from blastocyst-stage embryos, zygotes showing a multipolar division were cultured for 7 or 8 days, until they reached the blastocyst stage. Removal of the zona was followed by a wash step with TCM-199 with 10% FBS, and if present, large cells located in the perivitelline space were separated from the blastocyst. Cells of the blastocyst were further dissociated by a short incubation in trypsine-EDTA at 38.5°C, two wash steps in Ca^+2^/Mg^+2^-free PBS with 0.1% PVP and successive pipetting with a STRIPPER pipet holder, and 135 μm and 75 μm capillaries (Origio, Cooper Surgical, CT, US). All blastomeres or single cells were washed in Ca^+2^/Mg^+2^-free PBS with 0.1% PVP and transferred into a 0.2-mL PCR tube containing 2 or 4 μL of Ca^+2^/Mg^+2^-free PBS. For single cells isolated from blastocysts, a micromanipulation system was used to ensure transfer of a single cell. Tubed blastomeres/single cells were placed immediately on ice and stored at −80°C until whole-genome amplification (WGA). DNA from single blastomeres/single cells and, additionally, from entire blastocysts (sibling day-8) was whole-genome amplified by multiple displacement amplification (MDA) with a REPLI-g Single Cell Kit (Qiagen, Hilden, Germany) according to the manufacturer’s instructions with full or half reaction volumes for the fast 3-h protocol. The concentration of WGA DNA was determined by Qubit Broad Range Assay (Invitrogen, Carlsbad, CA, US A) according to the manufacturer’s protocol. Ovarian tissue from the donor cows (i.e., mothers of the respective embryos) and semen from the two bulls (i.e., fathers of the respective embryos) were used to extract bulk DNA (DNeasy Blood and Tissue kit, Qiagen, Hilden, Germany). Bulk DNA from the father and mother of one of the bulls (i.e., paternal grandparents of the respective embryos) was extracted identically from blood.

#### SNP genotyping and haplarithmisis

Some collected samples were not genotyped for technical (e.g., blastomere tubing errors, failed whole-genome amplification or limited spots on SNP arrays) or budgetary reasons. Finally, embryos or which or SNP array analysis failed in one or more blastomeres were not described in the manuscript and 2 embryos were excluded because the quality of the haplarithm plots prohibited trustworthy interpretation.

Whole-genome amplified products were normalized to 150 ng/μL (single cells and sibling embryos) or 50 ng/μL (bulk parental DNA) before downstream processing with the Infinium HD assay super protocol (Illumina, San Diego, CA, USA). Single-cell, multi-cell sibling, and bulk parental or grandparental DNA were genotyped on BovineHD BeadChips (Illumina, San Diego, California, USA). Subsequently, discrete genotypes, B-allele frequency (BAF) values, and LogR values were exported using Ilumina’s GenomeStudio and fed to a modified version of the siCHILD algorithm [64], siCHILD-bovine. Briefly, siCHILD-bovine is a computational workflow that deduces genome-wide (GW) single-cell haplotype and copy number profiles. For initial phasing of the parental genotypes, a blastocyst-stage sibling embryo (E01–E23 and E26–E32) or paternal grandparents (E24-E25) were used. Analysis of the final haplarithm plots according to siCHILD/haplarithmisis principles [64] enabled the characterization of polyploid blastomeres as either having an additional maternal or paternal copy (e.g., digynic or diandric triploidy, respectively) and blastomeres containing only paternal or maternal genomes to be labeled androgenetic or gynogenetic. Furthermore, the origin of the additional copies could be distinguished, being either mitotic or meiotic/polyspermic. A visual overview on the interpretation of the haplarithm plots can be consulted in Additional file 2: Fig. S2*.*

#### Low-coverage whole-genome sequencing and interpretation

For blastomeres or fragments without haplotypes, single-cell low-coverage whole-genome sequencing was performed. Sequencing libraries were prepared starting from 500 ng for each DNA sample with the KAPA HyperPrep Kit (Hoffman-La Roche, Basel, Switzerland) according to the manufacturer’s protocol. Single-end sequencing was performed on an Illumina Hiseq 4000 device (Illumina, San Diego, CA, US). The standard Illumina primary data analysis workflow was used for base calling and quality scoring. Next, reads were demultiplexed per sample and aligned to the reference bovine genome (BosTau8). The number of raw reads in non-overlapping 10-Mb bins were counted for the genomic and mitochondrial sequences and subsequently summarized in a box plot per chromosome and for the mitochondrial sequence. The mitochondrial genomes were assembled de novo with NOVOPlasty [133, 134]. The resulting assemblies were aligned against each other with MAFFT [135], followed by the construction of a phylogenetic tree with neighbor joining [136], which was visualized with Archaeopteryx [137].

### Nuclear staining

Individual blastomeres were fixed overnight in 4% paraformaldehyde and incubated in Hoechst 33342 (1:1000 dilution in PBS/PVP) for 10 min at room temperature in the dark to visualize the nuclei. Evaluation of the blastomere nuclear content was performed the next day by fluorescent microscopy with a Leica DM 5500 B microscope with excitation filter BP 450/90 nM and a 100-W mercury lamp. Blastomeres containing one nucleus or metaphase plate were considered mononuclear, and blastomeres containing no nuclear content were considered anuclear.

### Human embryo culture, biopsy, and blastocyst dissociation

The development and clinical implementation of concurrent GW haplotyping and copy number profiling of single blastomeres during preimplantation genetic testing (PGT) [64, 76, 77] provides a screen of genome-wide error profiles in human embryos fertilized through ICSI. In a retrospective study, 2300 day-3 single blastomere biopsies derived from 2257 cleavage-stage embryos which reached the blastocyst stage were analyzed [73]. Embryo culture, biopsy, and biopsy processing for these embryos were performed under a standard clinical workflow for PGT at UZ Leuven, as previously described by [138]. GW ploidy violations, such as haploidy/GW UPD and triploidy, were detected in 2.4%. These embryos are deemed not eligible for transfer. We classified those embryos as gynogenetic (carrying only maternal DNA) or androgenetic (carrying only paternal DNA), and triploid embryos as digynic or diandric, respectively. Two cleavage-stage embryos with a gynogenetic blastomere were followed up by thawing and dissociation of the resulting frozen blastocyst for single-cell analysis. Manipulations of the whole blastocyst were performed with a STRIPPER pipette with 175 or 135 μm capillaries (Origio, CooperSurgical, CT, US). The dissociation procedure was performed as follows: a short incubation of the blastocyst in Acidic Tyrode’s solution (Sigma-Aldrich, Schnelldorf, Germany) was executed until visual disappearance of the zona pellucida was observed. The blastocyst was consecutively washed in three drops of biopsy medium (LG PGD Biopsy Medium, Life Global) and incubated in trypsin at 37 °C. Subsequently, the blastocyst was washed three times in biopsy medium. Individual cells from the blastocysts were then isolated by manual pipetting using a STRIPPER pipette with a 75-μm capillary (Origio, CooperSurgical, CT, US) and washed three times 1% PVP-PBS. Subsequently, each isolated cell was transferred into a 0.2-mL PCR tube with 2 μL PBS and stored at −20°C until further use. Samples were then whole-genome amplified using REPLI-g Single Cell Kit (Qiagen, Hilden, Germany) for half-volume reactions and with incubation at 30°C for 2 h followed by 65°C during 10 min for inactivation. Analysis was performed using siCHILD/haplarithmisis [64], as mentioned previously under the standard clinical workflow for PGT at UZ Leuven. For both human blastocysts, E01 and E02, an (overall) biparental balanced single blastomere from a sibling embryo (for E02: a maternal meiotic trisomy 15 was present) was used to execute phasing of the blastocysts’ single cells.

### Statistical analyses and data visualization

Data were manually collected using the Primo Vision Analyzer Software and exported to Microsoft Excel (Microsoft Corp., Redmond, WA), where data exploration and organization were done using the PivotTables function (Microsoft Excel). The statistical analyses were performed using R-core (version 4.0.4; R Core Team, Vienna, Austria). Generalized mixed-effects models were used to test the effect of type of division (bipolar vs. multipolar) on blastocyst outcome at day 8 post insemination (yes vs. no). The cow (ovary) nested within the experimental replicate (2-level model) was chosen as a random effect to correct for the individual (cow) factor and eventual medium preparation variability among replicates. The differences between groups were assessed using Tukey’s post hoc test. Results are expressed as least squares means and standard errors. The significance level was set at *P* < 0.05. Circos plots were drawn using the Circlize package in R [139].

## Supplementary Information

Additional file 1: Movie S1. Time-lapse movies showing a bipolar zygotic division and a multipolar zygotic division in three or four cells, respectively.

Additional file 2: Figure S2. Analysis of blastomeres following multipolar zygotic division. A) Interpretation of haplarithm plots. Overview of chromosome-wise haplarithm patterns for distinct genomic constitutions (i.e. biparental disomy, paternal monosomy and paternal meiotic/dispermic uniparental heterodisomy). Corresponding whole-genome errors (i.e. biparental diploid or androgenetic) are characterized by the manifestation of those patterns throughout the (majority of the) genome. Defined single-cell BAF values of the segmented P1, P2, M1 and M2, form haplotype blocks, demarcated by pairwise breakpoints, i.e., homologous recombinations. Haplotype blocks, as well as the distance between the P1-P2 or M1-M2 in the paternal and maternal haplarithm, respectively, and the positioning of homologous recombinations, denote the origin and nature of copy number. The normalized LogR- values are integrated with haplarithm profiles for copy number profiling. Principles of interpretation are according to [64]. B) An overview of haplarithm profiles of 82 blastomeres and two fragments (grey squares) is depicted per category of whole-genome segregation profiles, as discussed in the main text. Each embryo is identified by a description at the top left of the embryo ID and cross (EmbryoID_Embryocross). At the top right, three chronological time-lapse images of the cleaving zygote are depicted. From left to right, the pictures show the initiation of the cleavage furrow, the ongoing first division and the embryo immediately after cleavage and before cell isolation (when video available). For each embryo, a schematic representation of likely steps leading to the genomic profile of each blastomere (B1-B4) or fragment (F1) is given. Chromosome-wise interpretation (1 - X) per blastomere is visualized in the bar above the haplarithm plots (see legend). Below each bar, the paternal haplarithm (pat-BAF), the maternal haplarithm (mat-BAF) and the normalized LogR values (LogR) are depicted. Paternal cross-over sites are depicted by the arrows (black, green or blue). A combination of parental cross-over sites in one blastomere or different cross-over sites in blastomeres of the same embryo uncover polyspermic fertilization or a meiotic error. Maternal cross-over sites (red, pink, orange) were only depicted in gynogenetic blastomeres and in case of whole-genome meiotic errors.

Additional file 3: Movie S3_3A.

Additional file 4: Movie S4_3B.

Additional file 5: Movie S5_3C.

Additional file 6: Movie S6_3D.

Additional file 7: Movie S7_3E. Live-imaging movies showing spindle assembly and cytokinesis in a bovine zygote cleaving in two cells and in the four bovine zygotes cleaving directly into three or four cells (multipolar zygotic division). Additional file 3: Movie S3_3A corresponds to the selected time points in Fig. 3A and shows the assembly of a biparental spindle in a 2PN zygote and the formation of two biparental mononucleated blastomeres. Additional file 4: Movie S4_3B corresponds to the selected time points in Fig. 3B and shows the assembly of a biparental spindle in a 2PN zygote and the formation of one anuclear blastomere and two biparental mononucleated blastomeres. Additional file 5: Movie S5_3C corresponds to Fig. 3C and shows the assembly of two private parental spindle in a 2PN zygote and the formation of one anuclear blastomere, one mono-parental mononucleated and one biparental multinucleated blastomere with an extra copy of one parental genome. Additional file 6: Movie S6_3D corresponds to Fig. 3D and shows the assembly of a biparental spindle and an asynchronous lagging mono-parental spindle in a 3PN zygote and the formation of two biparental blastomeres and the extrusion of a parental genome into a separate blastomere. Additional file 7_ Movie S7_3E corresponds to Fig. 3E and shows the assembly of an additional mono-parental spindle and a biparental spindle (with one PN showing asynchronous NEBD) in a 3PN zygote and the formation of two mono-parental mononucleated blastomeres and two biparental mononucleated biparental blastomeres containing micronuclei.

Additional file 8: Figure S8. Anuclear blastomeres and fragments. A) Phylogenetic tree based on reassembled mitochondrial genomes of 11 anuclear blastomeres and two anuclear fragments, showing a common ancestor for sequenced blastomeres and fragments retrieved from the same embryo. One anuclear blastomere was not included. B) Box plots and median values of the number of raw reads per 10 Mb bin (y-axis) per chromosome (chr1 - chrX) and for the mitochondrial DNA (chrM) (x-axis) as determined by single-cell low-coverage whole-genome sequencing for 12 anuclear blastomeres and two anuclear fragments demonstrate the abundance of mitochondrial DNA. C) Identical plots as in B, excluding chr M demonstrate the presence of fragments of chromosomal fragments in three anuclear blastomeres. D) Overlay of bright field and Hoechst fluorescent image show anuclear (1) and mononucleated (2;3) blastomeres resulting from a multipolar zygotic division in three blastomeres.

Additional file 9: Figure S9. Genome-wide composition of blastocysts following multipolar zygotic division. Circos plots of six embryos that developed to the blastocyst stage following multipolar zygotic division in which each circle represents the interpreted genome constitution per chromosome (1 - X) of a sampled single cell. The interpreted genome spanning the largest part of the chromosome was chosen as overall interpretation per chromosome, as such, segmental chromosomal errors are not depicted. On the left of each circus plot, a picture is shown of each embryo before dissociation (not available for E26_Cross13).

Additional file 10. Review history.

## Data Availability

Human embryo, parental and phasing relatives’ raw genotyping data has been deposited at the European Genome-phenome Archive (EGA), which is hosted by the EBI and the CRG, under accession number EGAS00001005543 (https://ega-archive.org/studies/EGAS00001005543) [140]. It is available to academic users upon request to the Data Access Committee (DAC) of KU Leuven via the corresponding author (J.R.V.). The bovine sequencing data for this study have been deposited in the European Nucleotide Archive (ENA) at EMBL-EBI under accession number PRJEB46925 (https://www.ebi.ac.uk/ena/browser/view/PRJEB46925) [141]. The bovine SNP array genotyping data discussed in this publication have been deposited in NCBI’s Gene Expression Omnibus and are accessible through GEO Series accession number GSE182345 (https://www.ncbi.nlm.nih.gov/geo/query/acc.cgi?acc= GSE182345) [142]. In compliance with the GDPR (General Data Protection Regulation 2016/679) and the study protocol, the human PGT-M blastocyst dataset used in the study is not publicly available. The live-imaging data have been deposited by the research data repository DataverseNL (dataverse.nl) (10.34894/AQ9OIU) [143].
