# Supplementary material for: Parental genomes segregate into distinct blastomeres during multipolar zygotic divisions leading to mixoploid and chimeric blastocysts
Source: Genome Biol. 2022 Oct 3;23:201. doi: 10.1186/s13059-022-02763-2 (PMC9528162; doi:10.1186/s13059-022-02763-2)

Figure S2

Analysis of blastomeres following multipolar zygotic division. A) Interpretation of haplarithm plots. Overview of chromosome-wise haplarithm patterns for distinct genomic constitutions (i.e. biparental disomy, paternal monosomy and paternal meiotic/dispermic uniparental heterodisomy). Corresponding whole-genome errors (i.e. biparental diploid or androgenetic) are characterized by the manifestation of those patterns throughout the (majority of the) genome. Defined single-cell BAF-values of the segmented P1, P2, M1 and M2, form haplotype blocks, demarcated by pairwise breakpoints, i.e., homologous recombinations. Haplotype blocks, as well as the distance between the P1-P2 or M1-M2 in the paternal and maternal haplarithm, respectively, and the positioning of homologous recombinations, denote the origin and nature of copy number. The normalized logR- values are integrated with haplarithm profiles for copy number profiling. Principles of interpretation are according to (64). **B)** An overview of haplarithm profiles of 82 blastomeres and two fragments (grey squares) is depicted per category of whole-genome segregation profiles, as discussed in the main text. Each embryo is identified by a description at the top left of the embryo ID and cross (EmbryoID_Embryocross). At the top right, three chronological time-lapse images of the cleaving zygote are depicted. From left to right, the pictures show the initiation of the cleavage furrow, the ongoing first division and the embryo immediately after cleavage and before cell isolation (when video available). For each embryo, a schematic representation of likely steps leading to the genomic profile of each blastomere (B1-B4) or fragment (F1) is given. Chromosome-wise interpretation (1 - X) per blastomere is visualized in the bar above the haplarithm plots (see legend). Below each bar, the paternal haplarithm (pat-BAF), the maternal haplarithm (mat-BAF) and the normalized LogR-values (LogR) are depicted. Paternal cross-over sites are depicted by the arrows (black, green or blue). A combination of parental cross-over sites in one blastomere or different cross-over sites in blastomeres of the same embryo uncover polyspermic fertilization or a meiotic error. Maternal cross-over sites (red, pink, orange) were only depicted in gynogenetic blastomeres and in case of whole-genome meiotic errors.

Figure S2

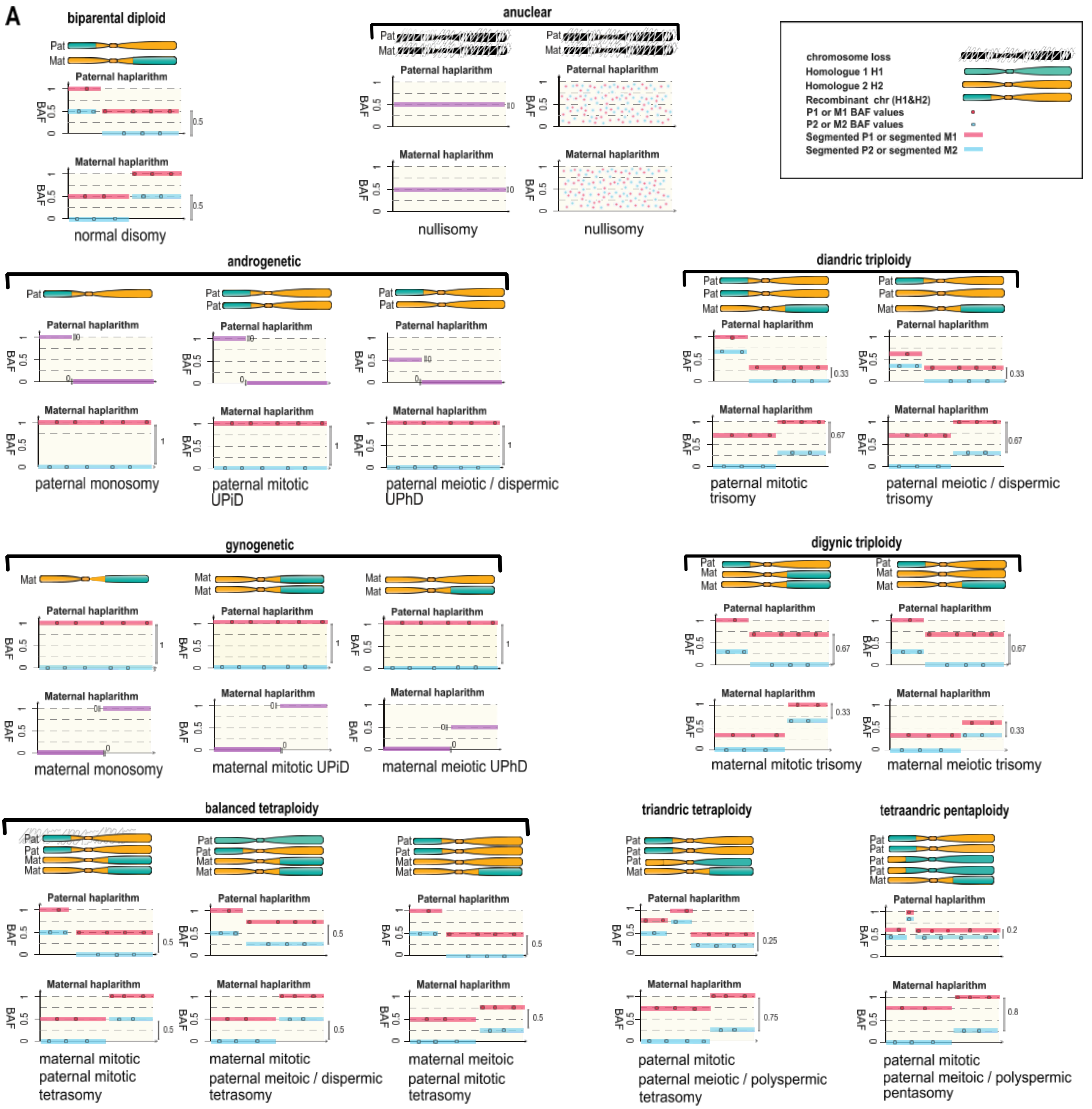

B

Legend

- biparental disomy
- paternal only
- maternal only
- diandric trisomy
- digynic trisomy
- tetrasomy
- pentasomy
- hexasomy
- nullisomy
- low quality nuclear, presumed biparental disomy
- low quality nuclear, presumed paternal
- low quality nuclear, undetermined

x1 uniparental chromosomal loss

x2 uniparental chromosomal gain

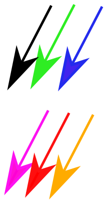

Each color points towards homologous recombination sites of different paternal haplotypes.

Each color points towards homologous recombination sites of different maternal haplotypes.

Figure S2B (continued)

1. Embryos consisting of diandric triploid, biparental diploid and androgenetic blastomeres

E05_Cross03

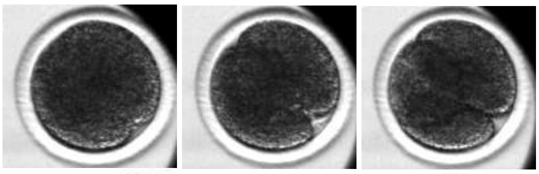

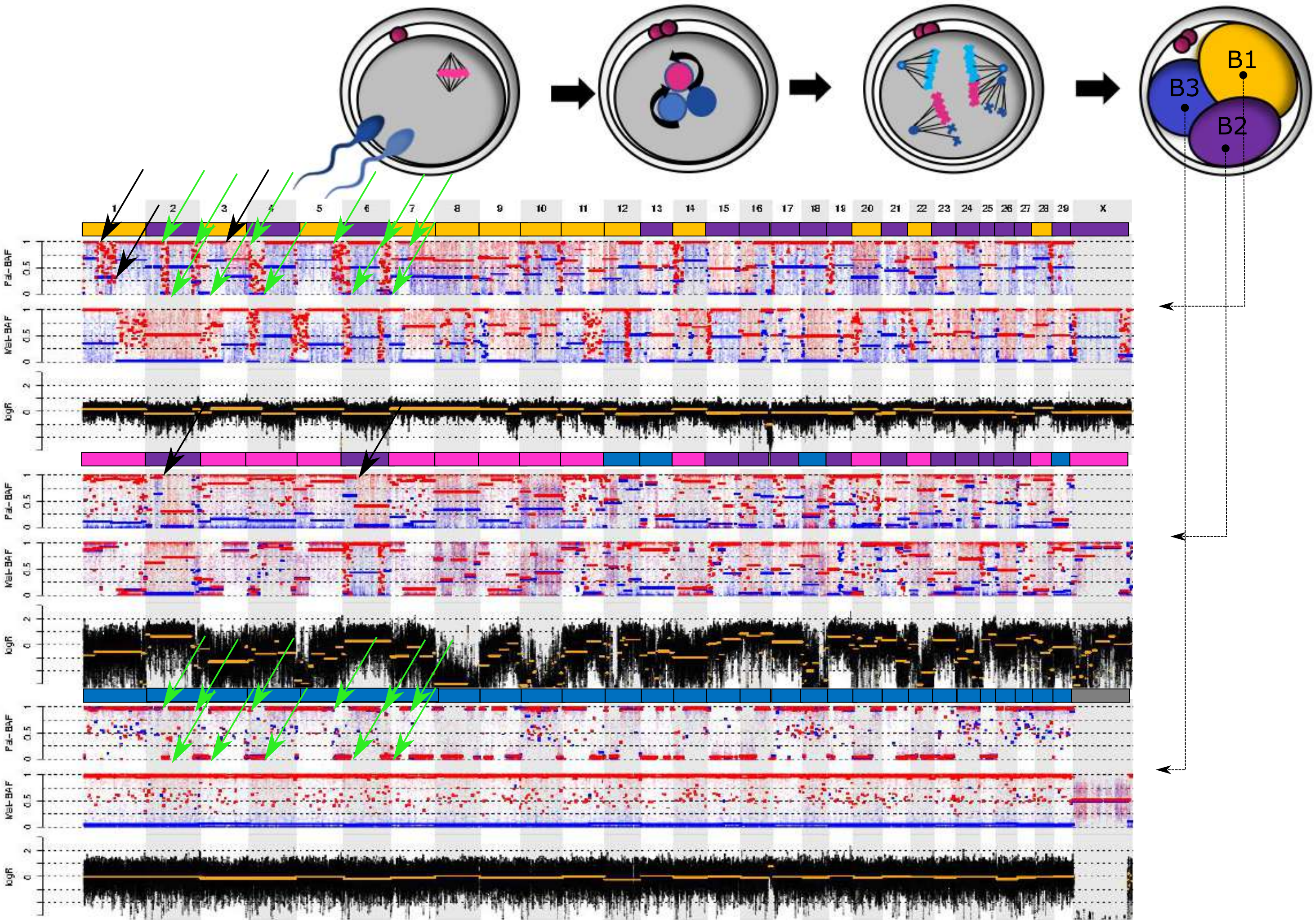

Figure S2B (continued)

E07_Cross04

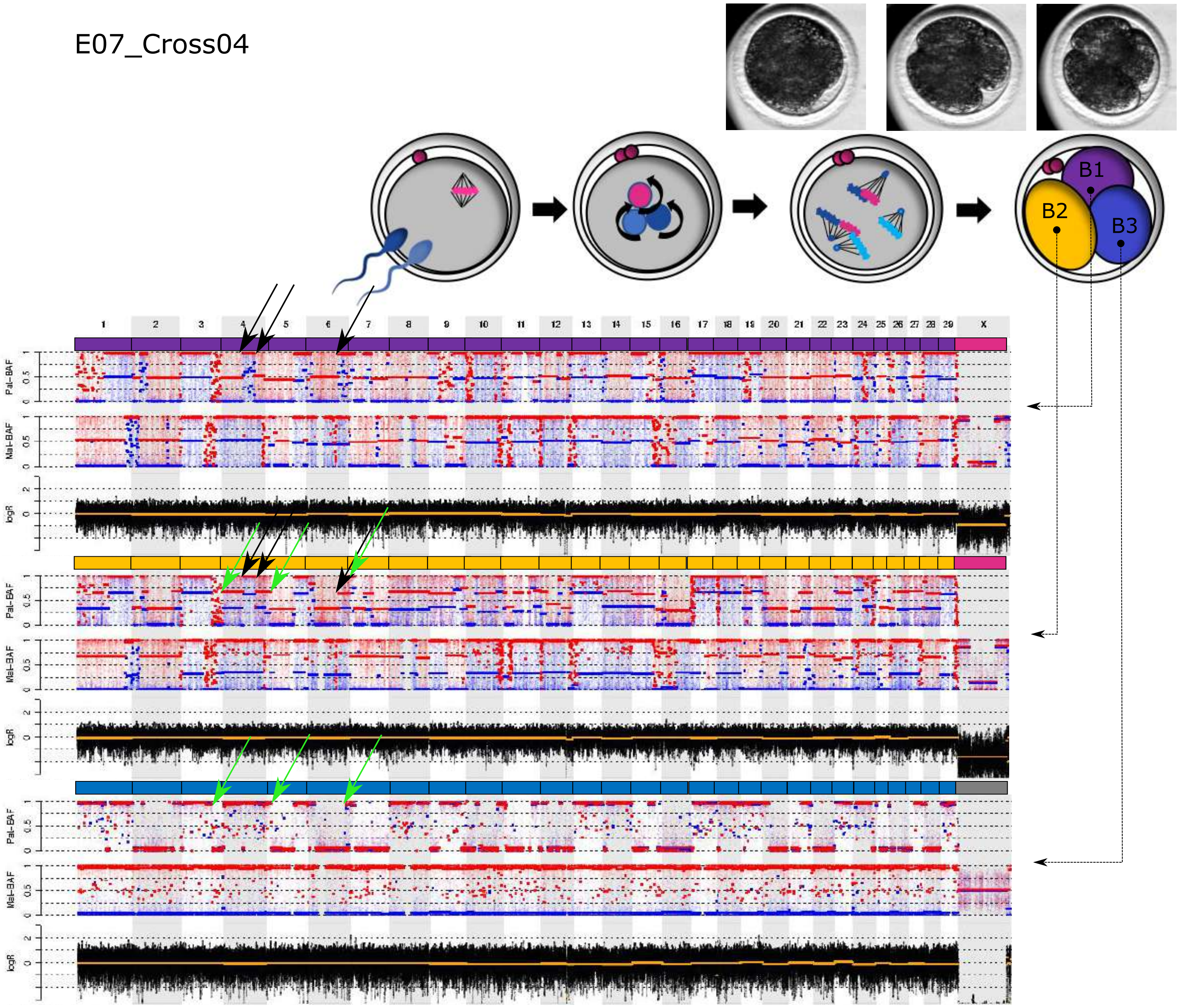

Figure S2B (continued)

E09_Cross04

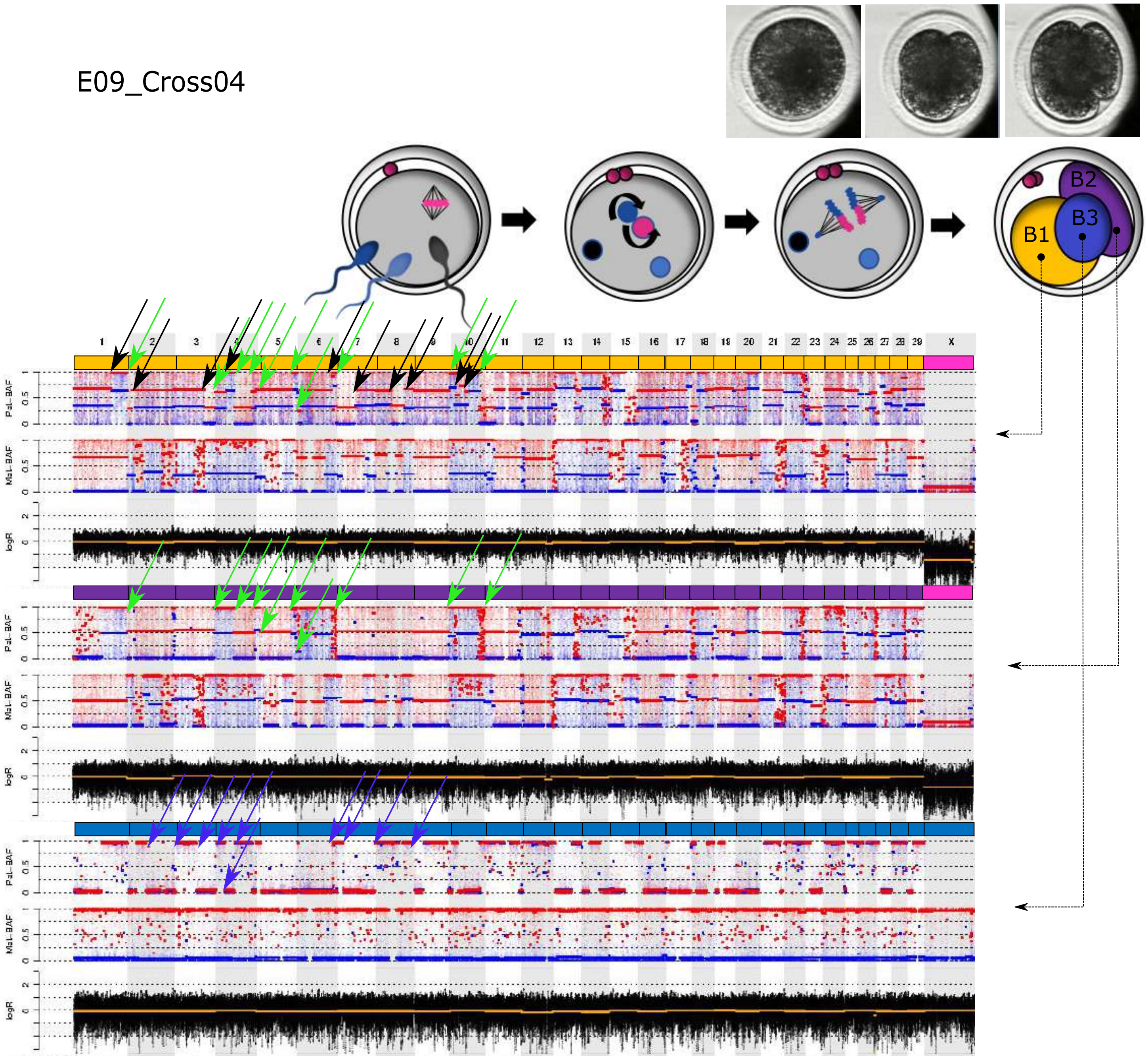

Figure S2B (continued)

2. Embryos consisting of biparental and androgenetic blastomeres

Some embryos harbored complexities additional to those discussed in the main text and Fig. 4. In one androgenetic blastomere of E06, regions of heterodisomy throughout the genome indicated the presence of three paternal copies of two distinct haplotypes. This might have occurred via extrusion of one replicated and one non-replicated paternal pronucleus into the same androgenetic blastomere. The simultaneous presence of a digynic triploid blastomere with two identical maternal copies in the same embryo, pointed towards the apposition of two parental nuclei, but replication of only the maternal nucleus before the zygotic division. In E12, four paternal haplotypes were present of which two were included in the tetraploid biparental cell with balanced parental genomes containing only one maternal haplotype. Hence, fertilization occurred by four sperm and two paternal genomes were extruded in a different blastomere. The other paternal genomes and the replicated maternal genome remained in the tetraploid blastomere. Regions of heterodisomy throughout the genome in an androgenetic blastomere of E15 hinted to the involvement of an inactive, third paternal nucleus during the segregation of the second paternal pronucleus by an additional paternal spindle. Moreover, lost chromosomes throughout the genome of the other androgenetic blastomere suggested the partial loss of the paternal nucleus segregated by the ectopic spindle. In E16, (segmental) maternal chromosomes were missing throughout the genome, likely due to a meiotic error.

Figure S2B (continued)

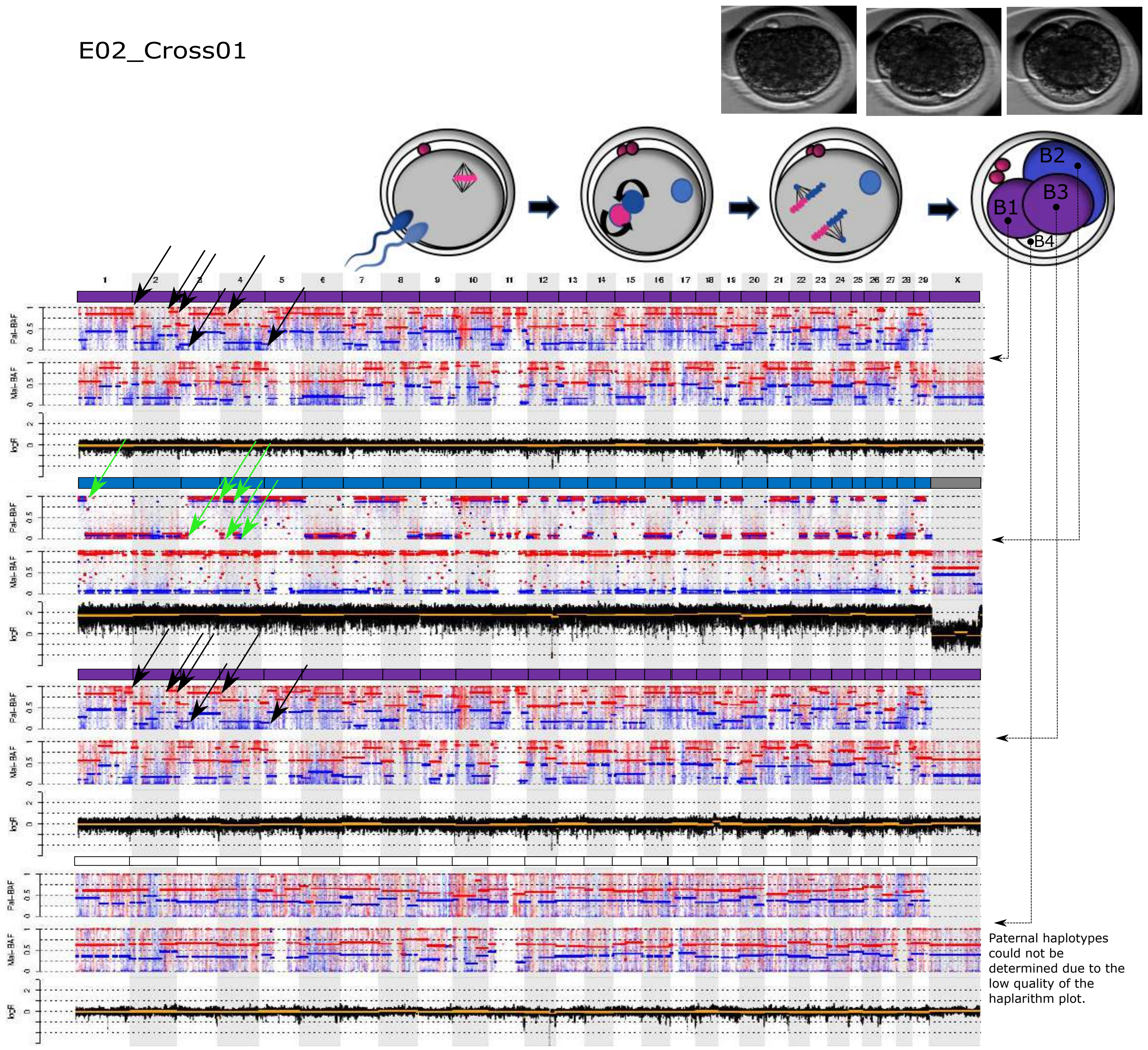

Figure S2B (continued)

E06_Cross04

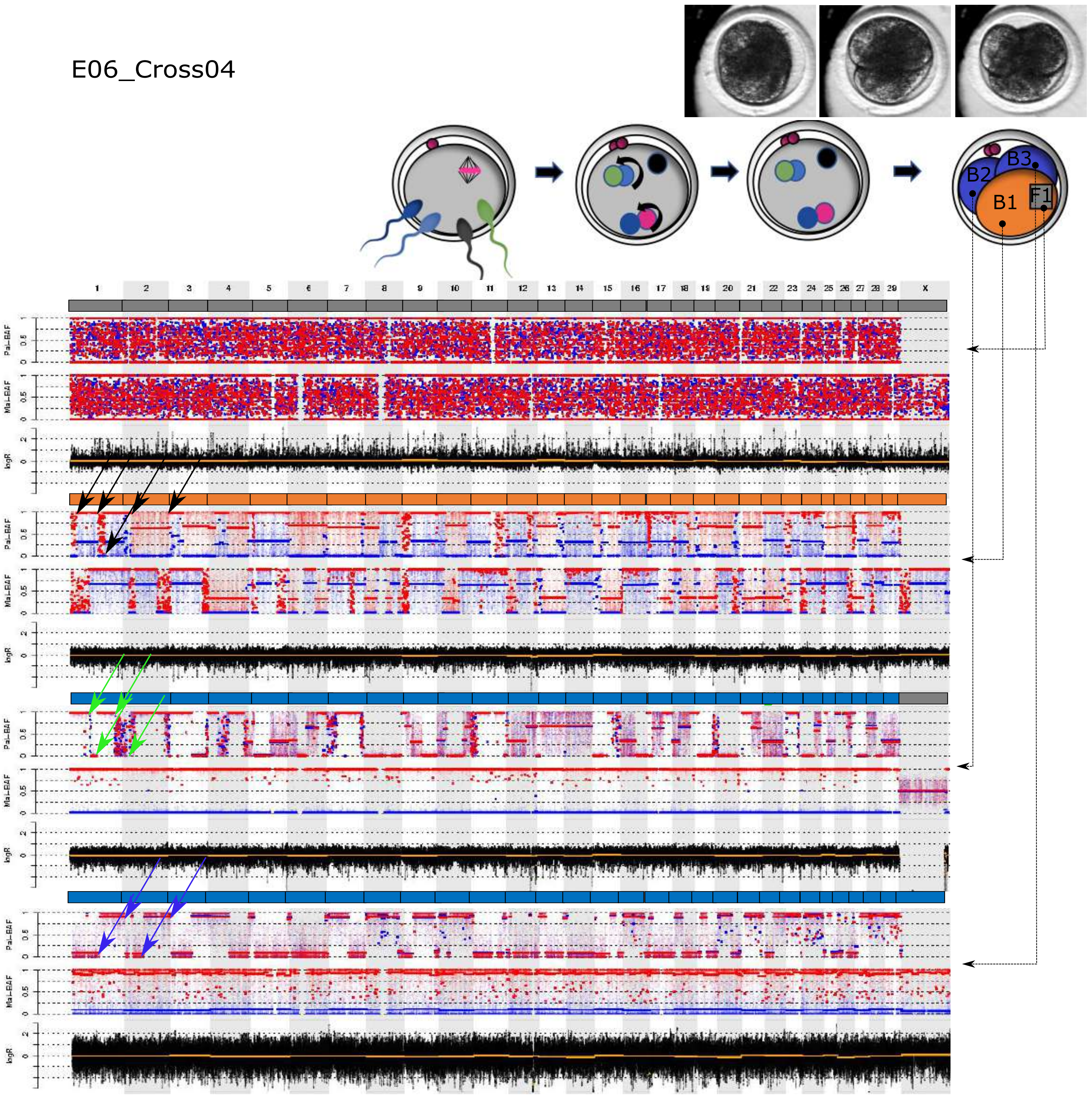

Figure S2B (continued)

E11_Cross05

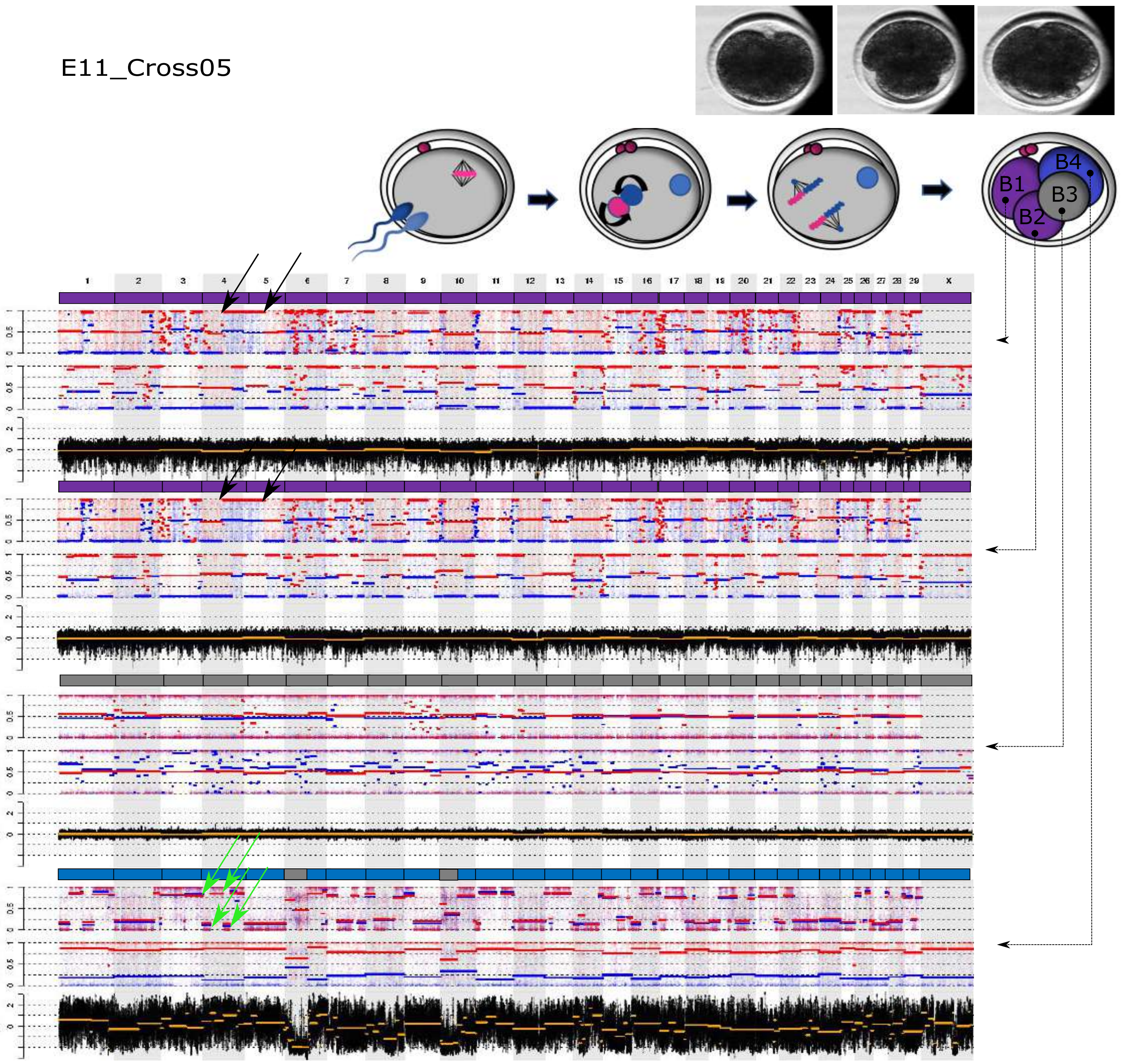

Figure S2B (continued)

E12_Cross05

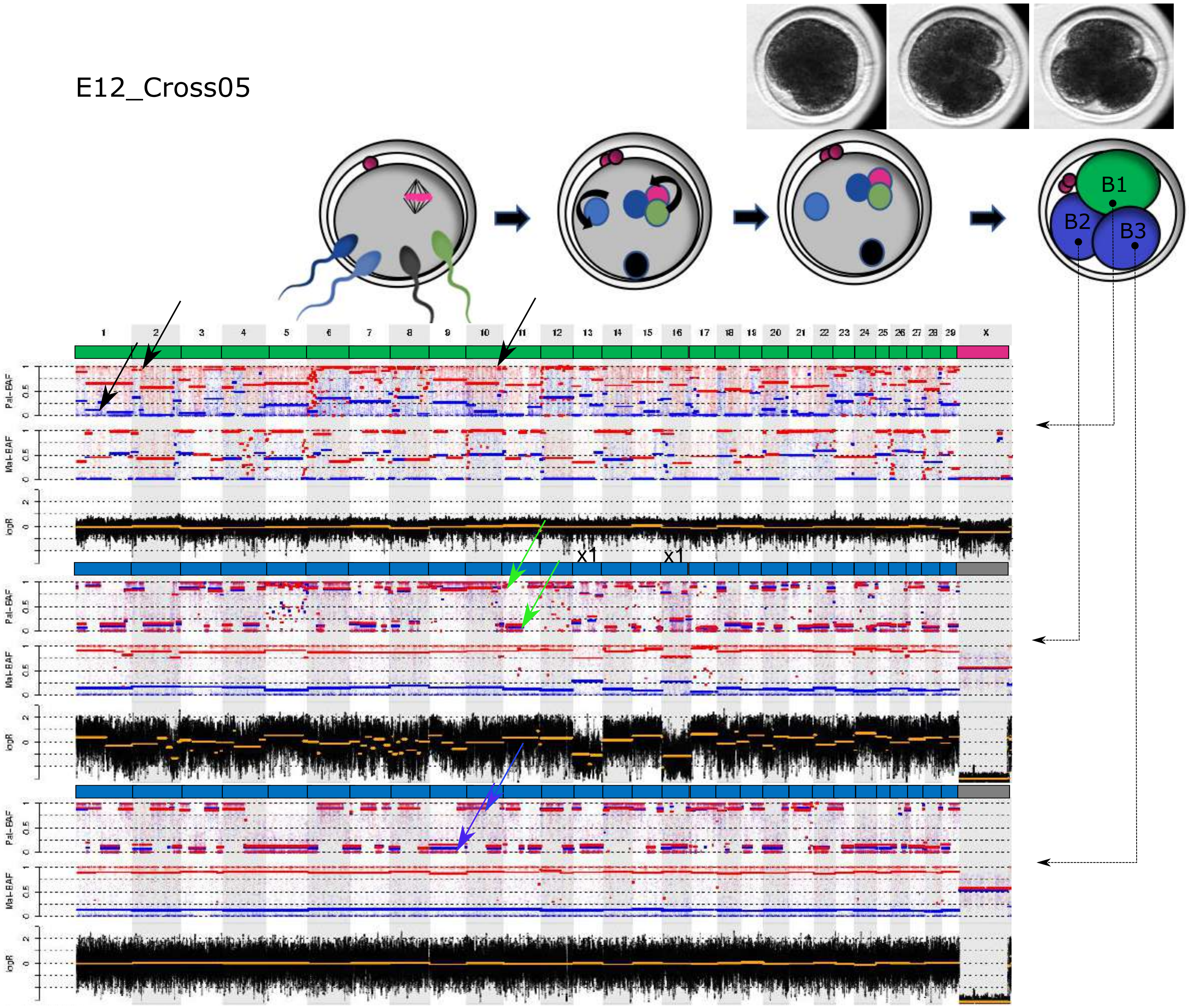

Figure S2B (continued)

E13_Cross11

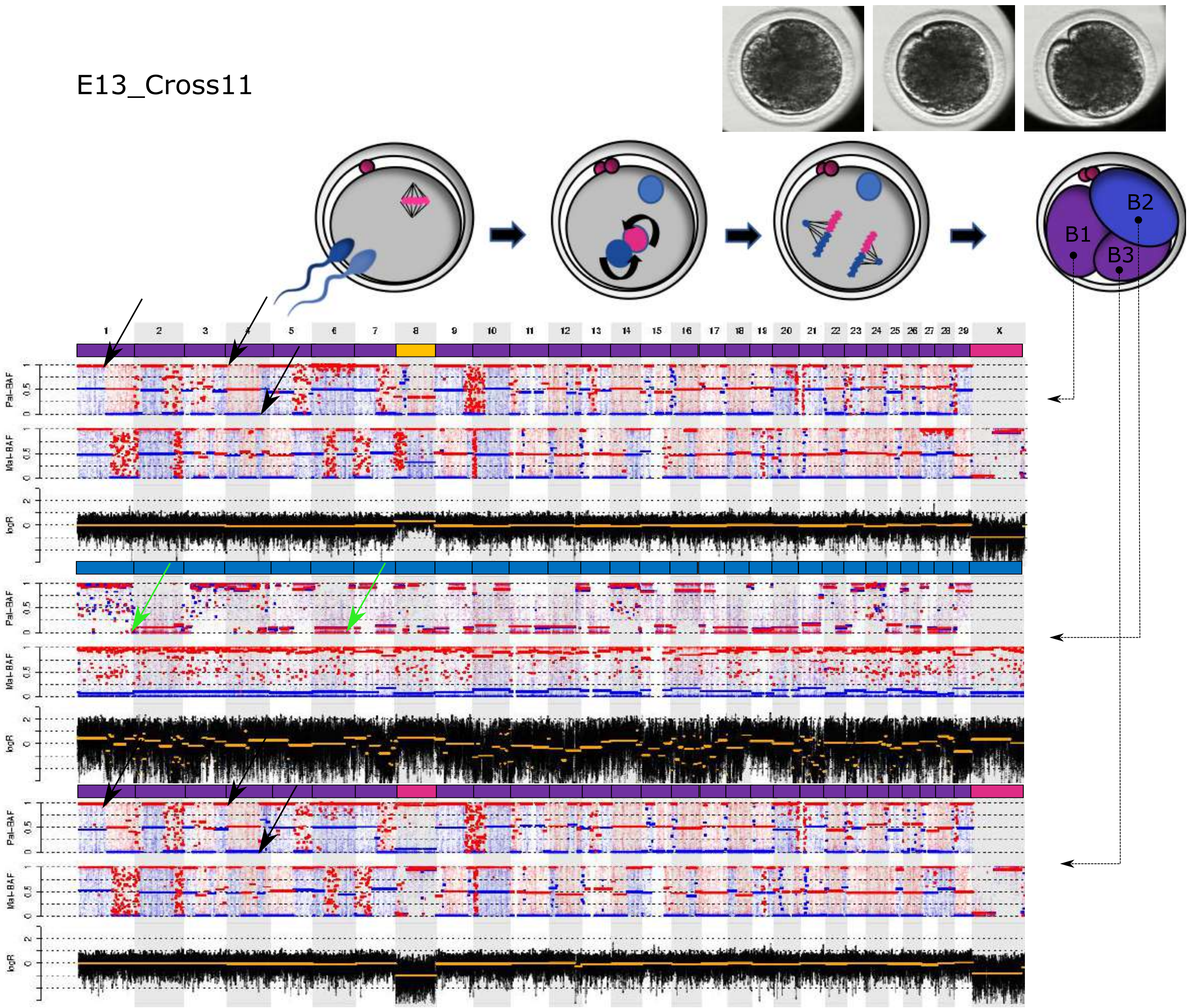

Figure S2B (continued)

E14_Cross07

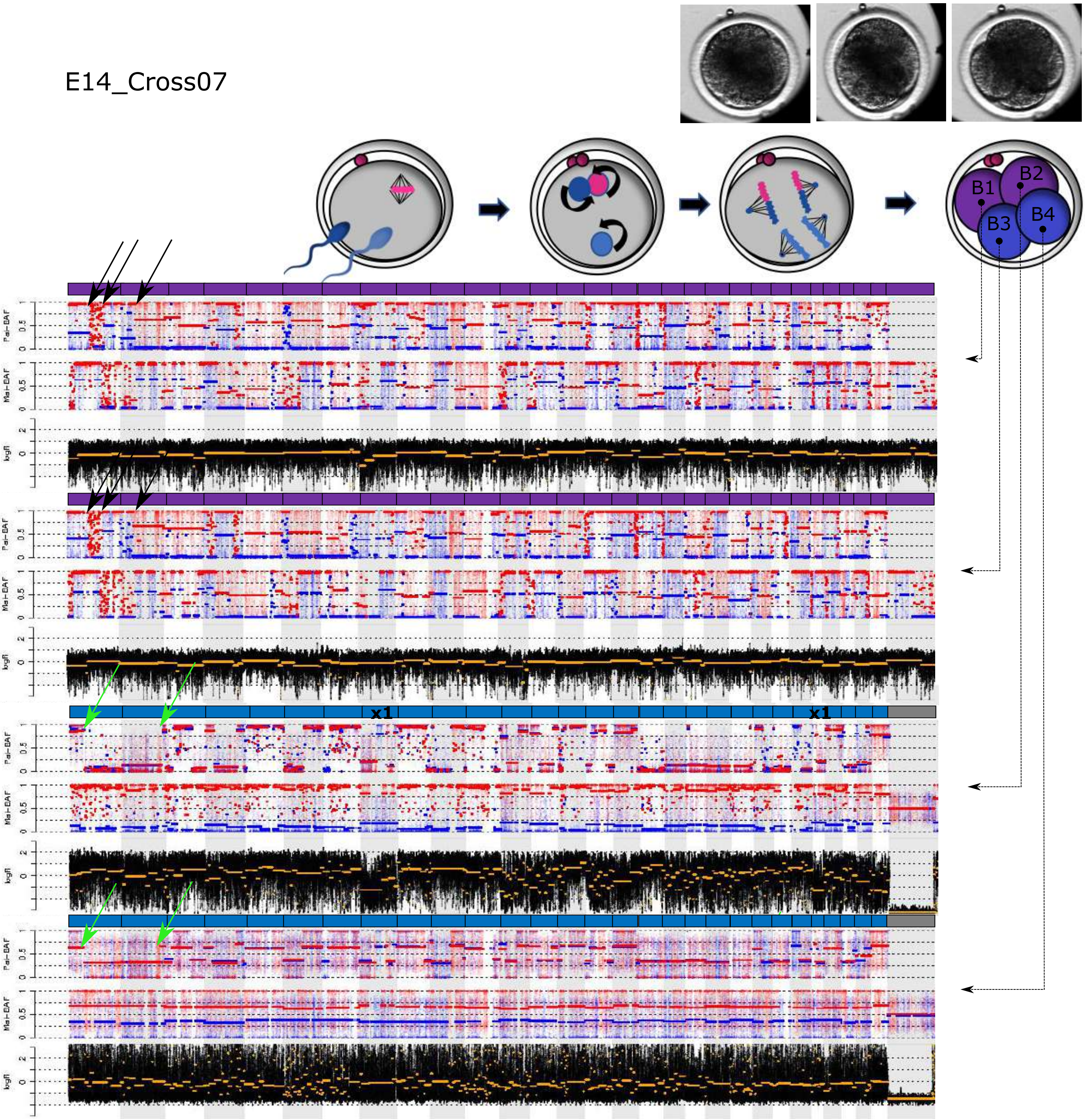

Figure S2B (continued)

E15_Cross07

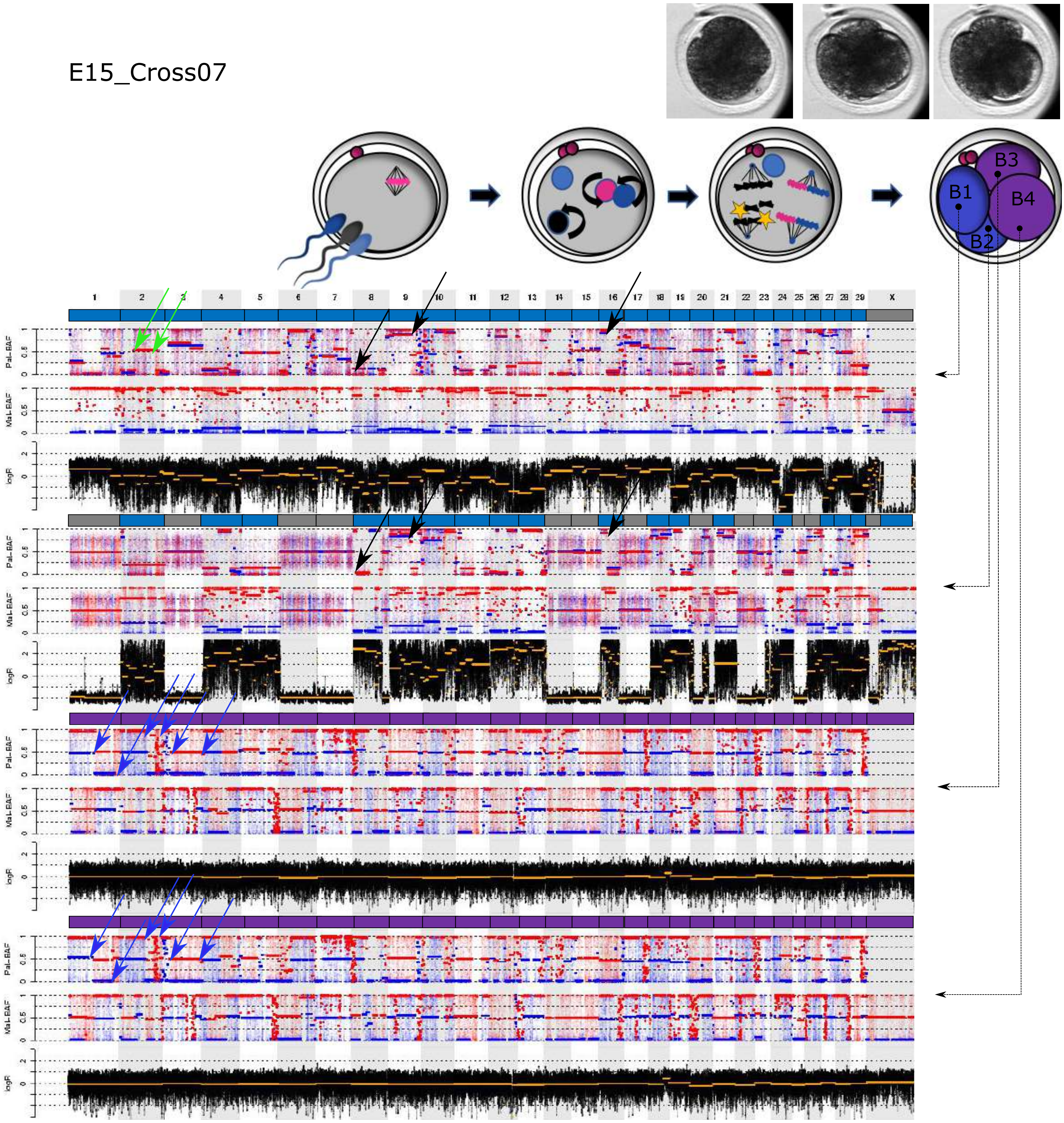

Figure S2B (continued)

E16_Cross07

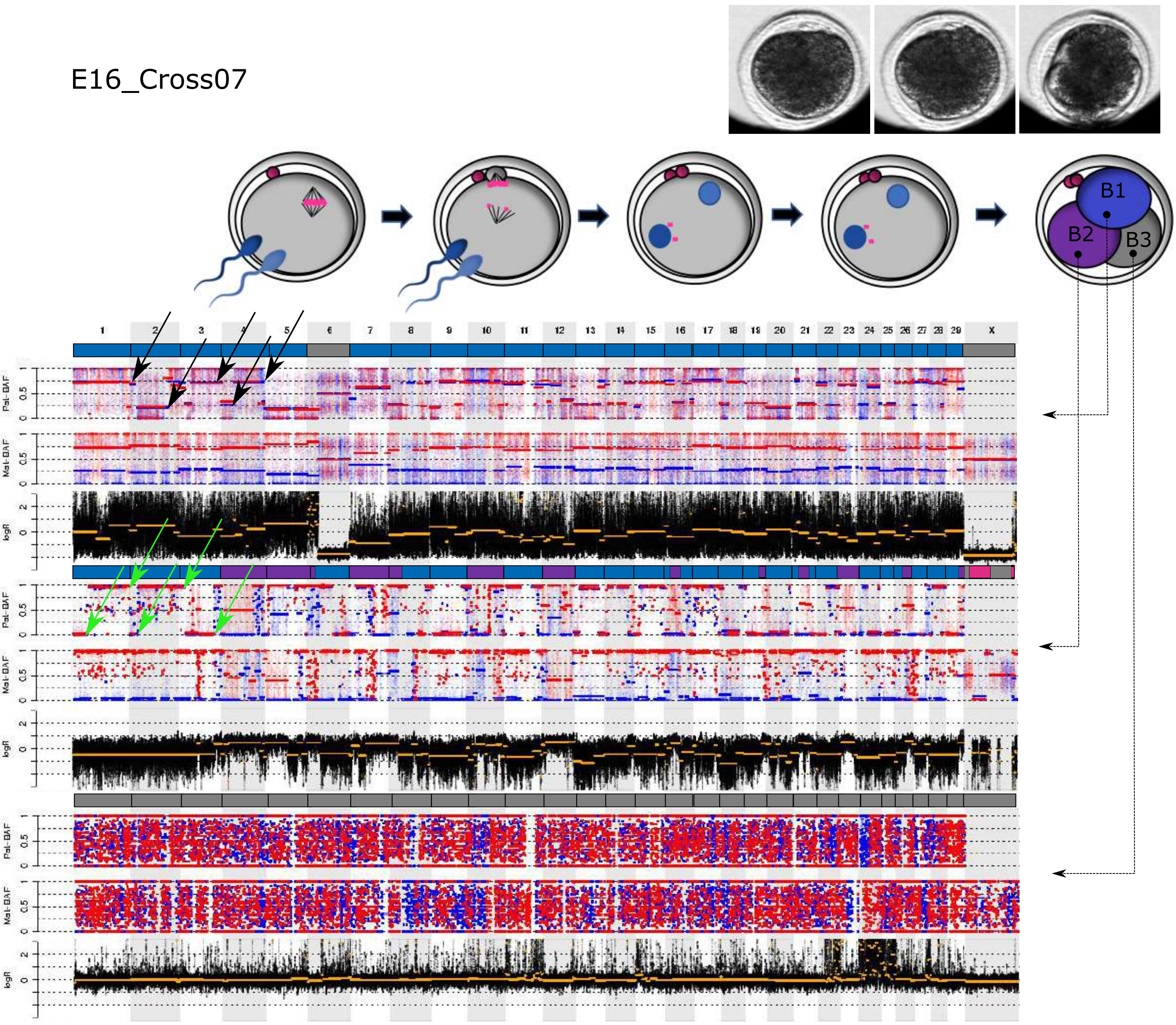

Figure S2B (continued)

E17_Cross07

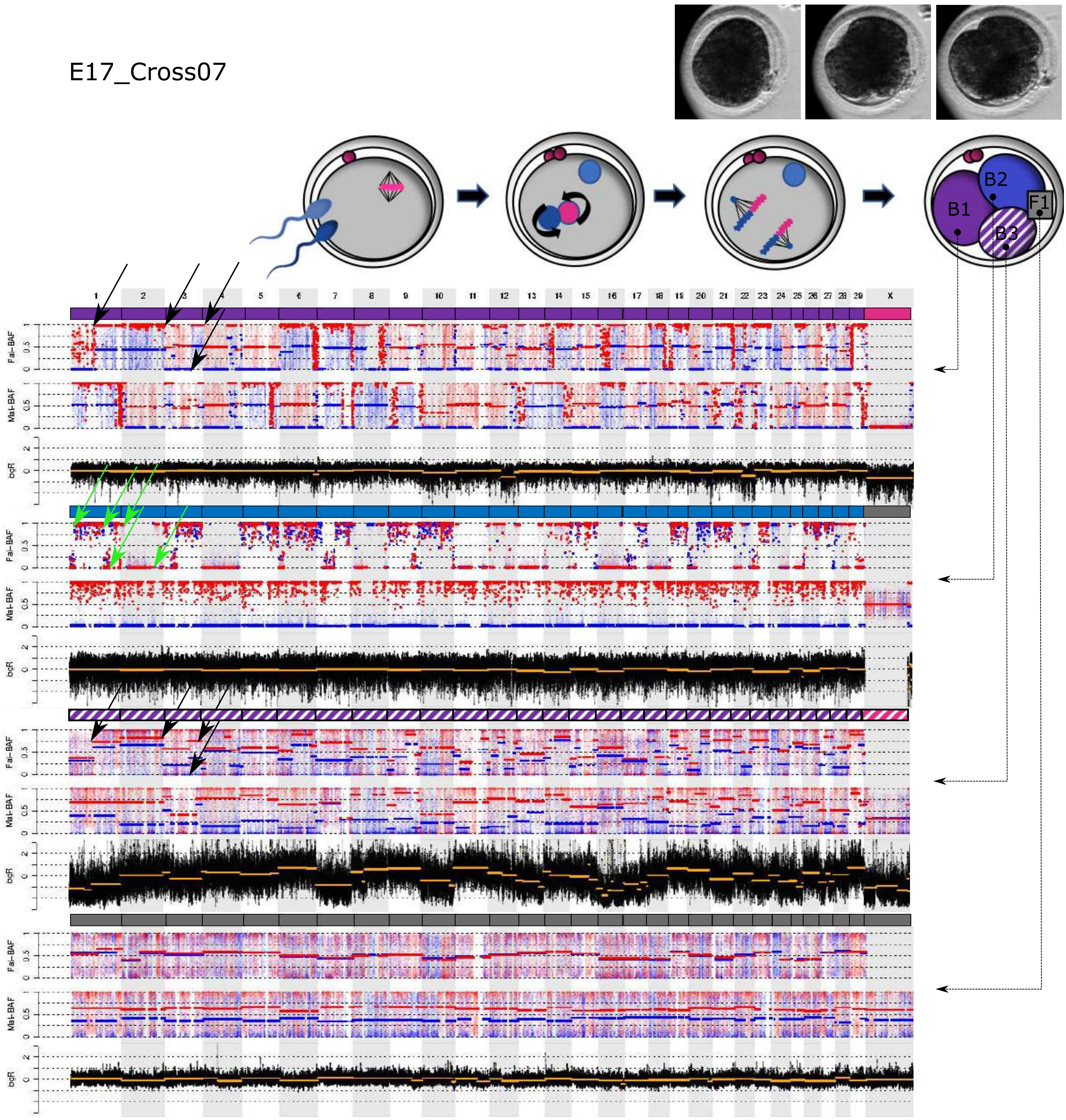

Figure S2B (continued)

E18_Cross08

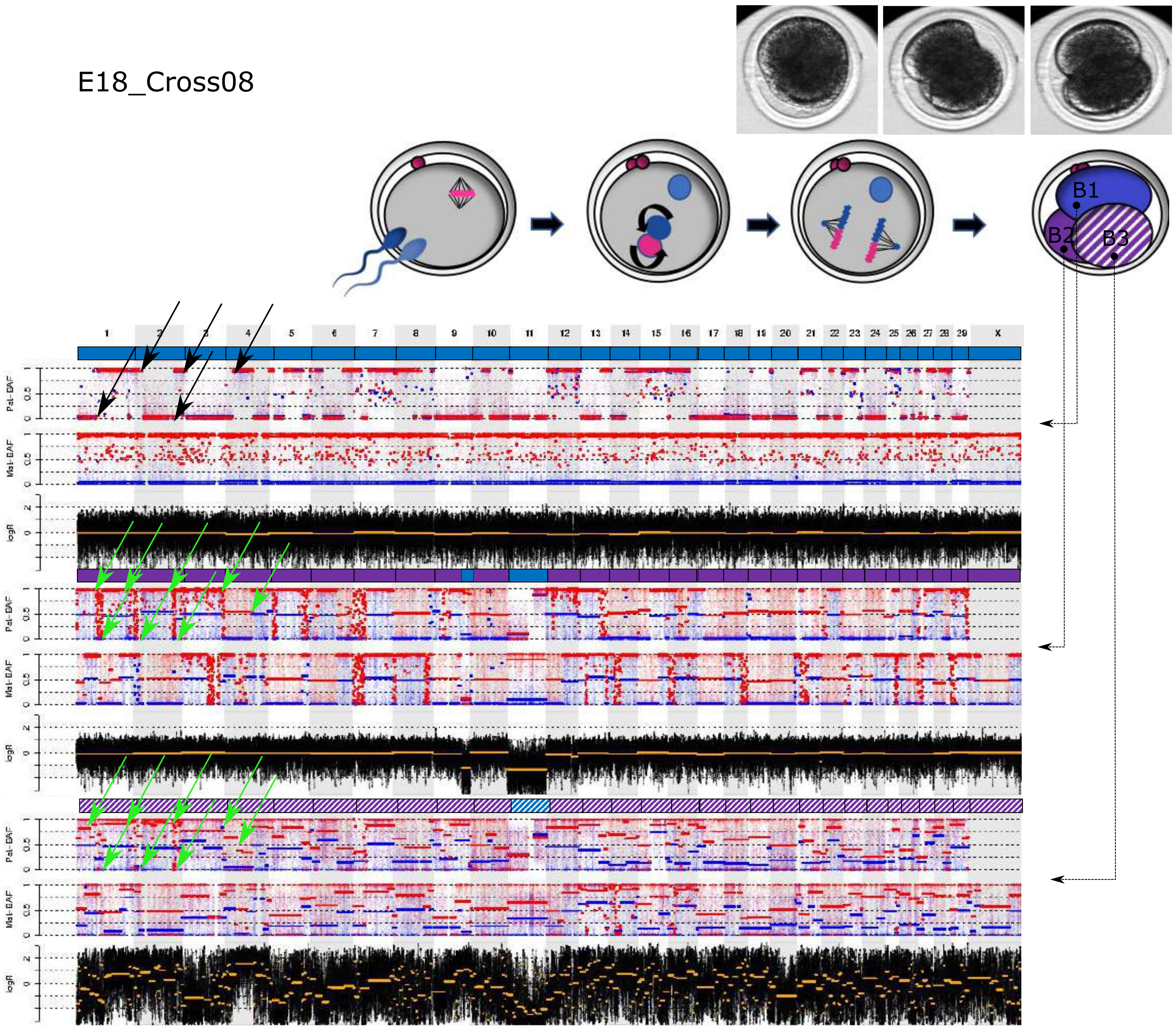

Figure S2B (continued)

E19_Cross08

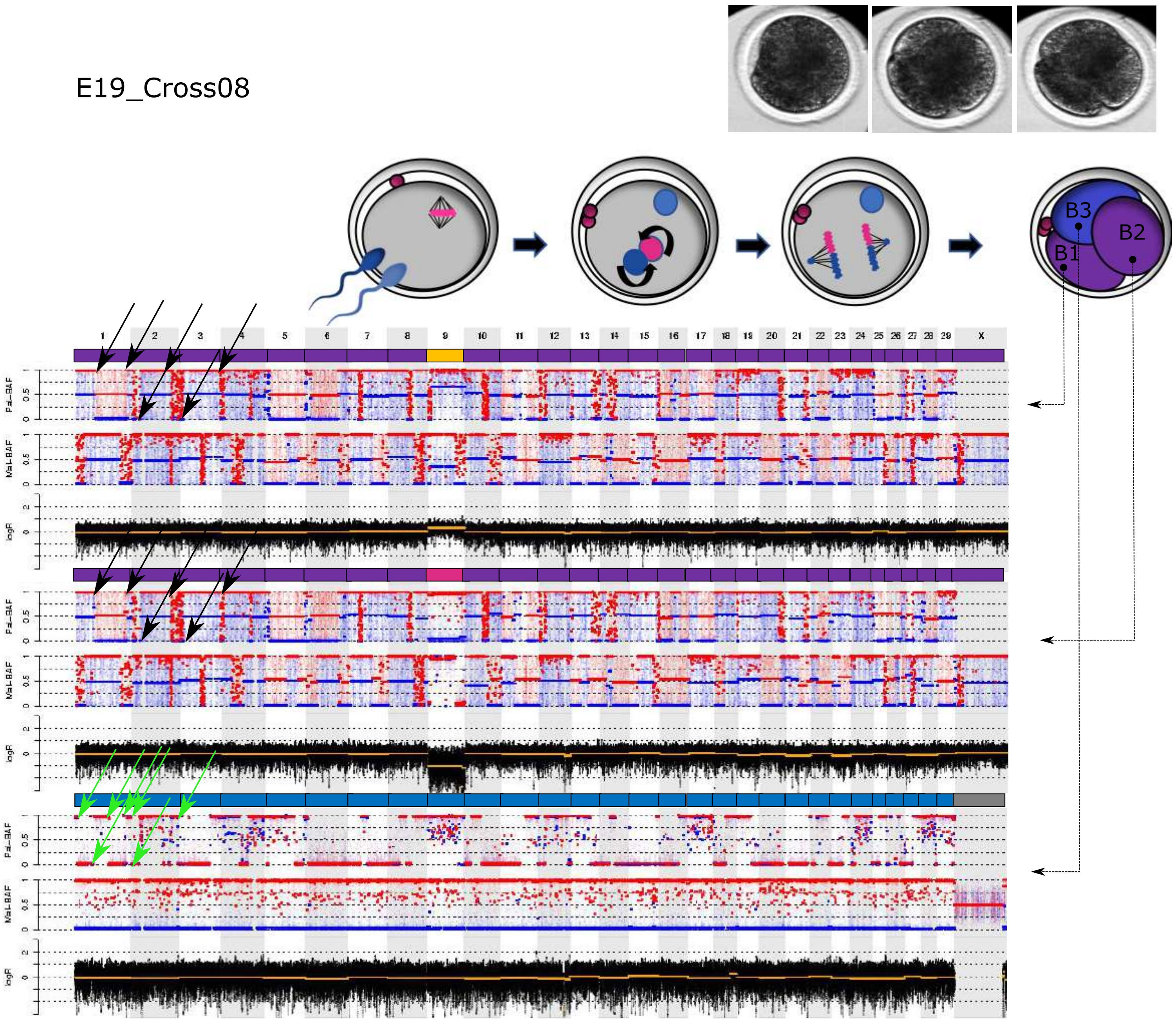

Figure S2B (continued)

E21_Cross09

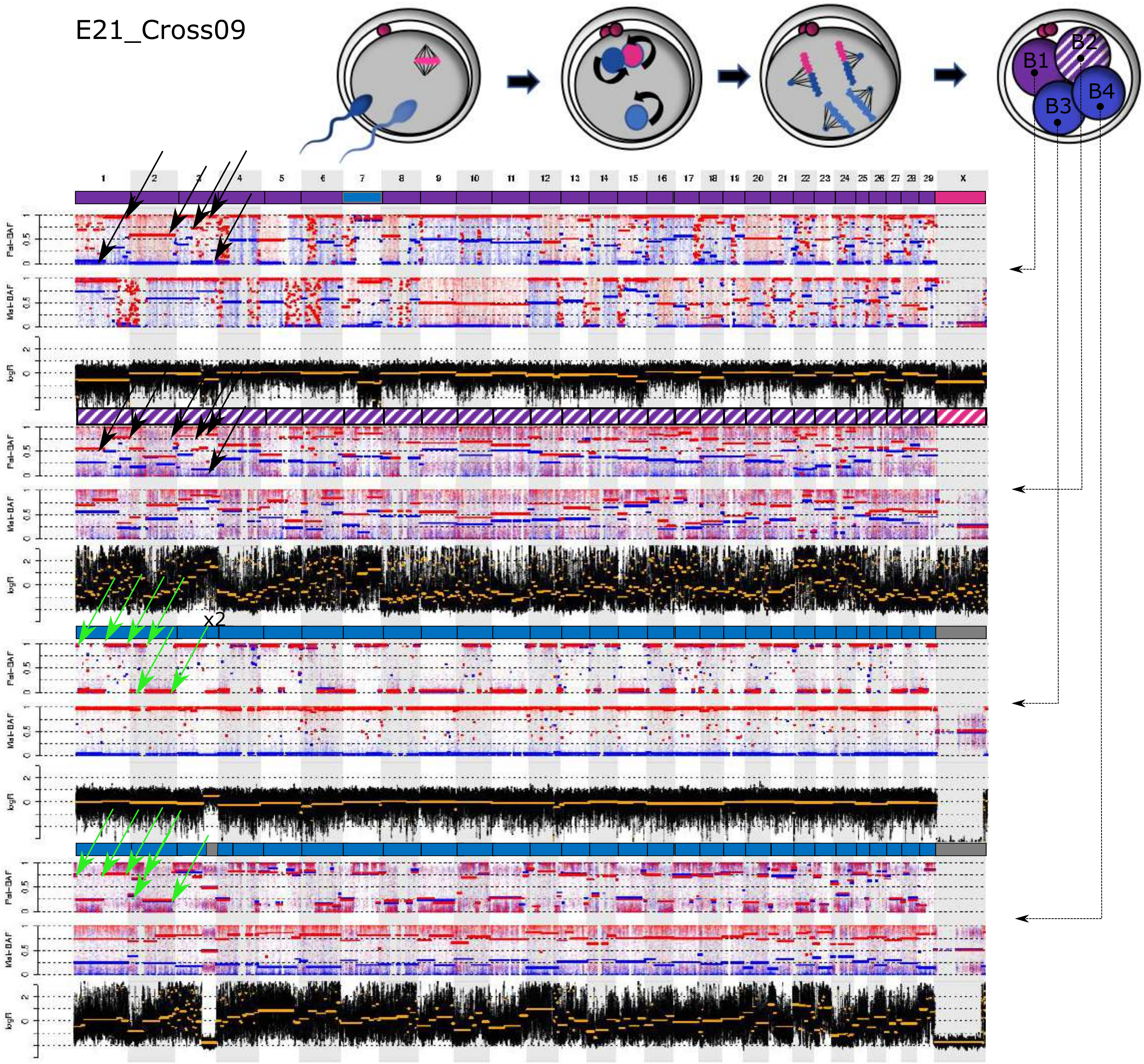

Figure S2B (continued)

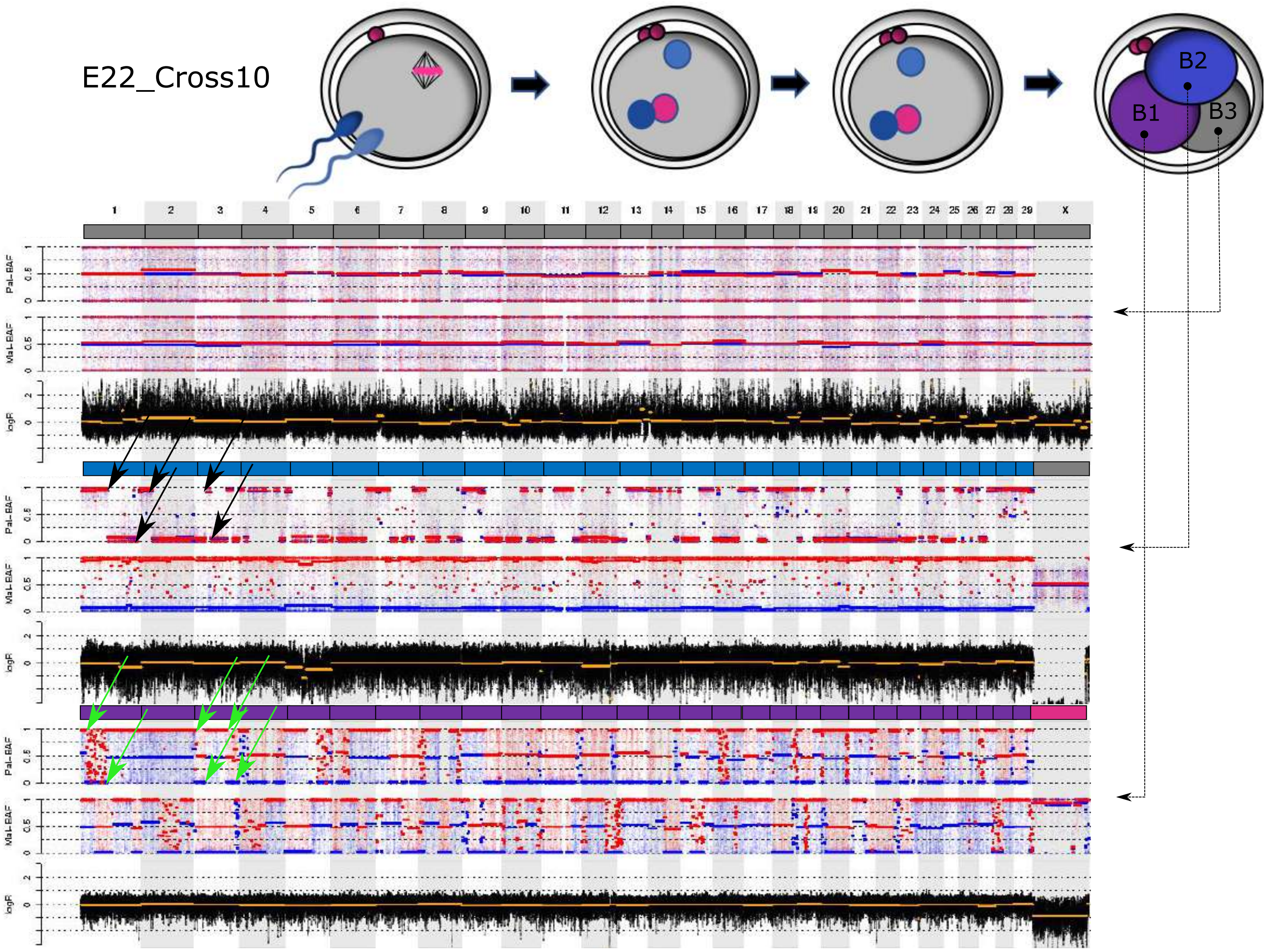

Figure S2B (continued)

3. Embryos consisting of androgenetic and gynogenetic blastomeres

E23_Cross11

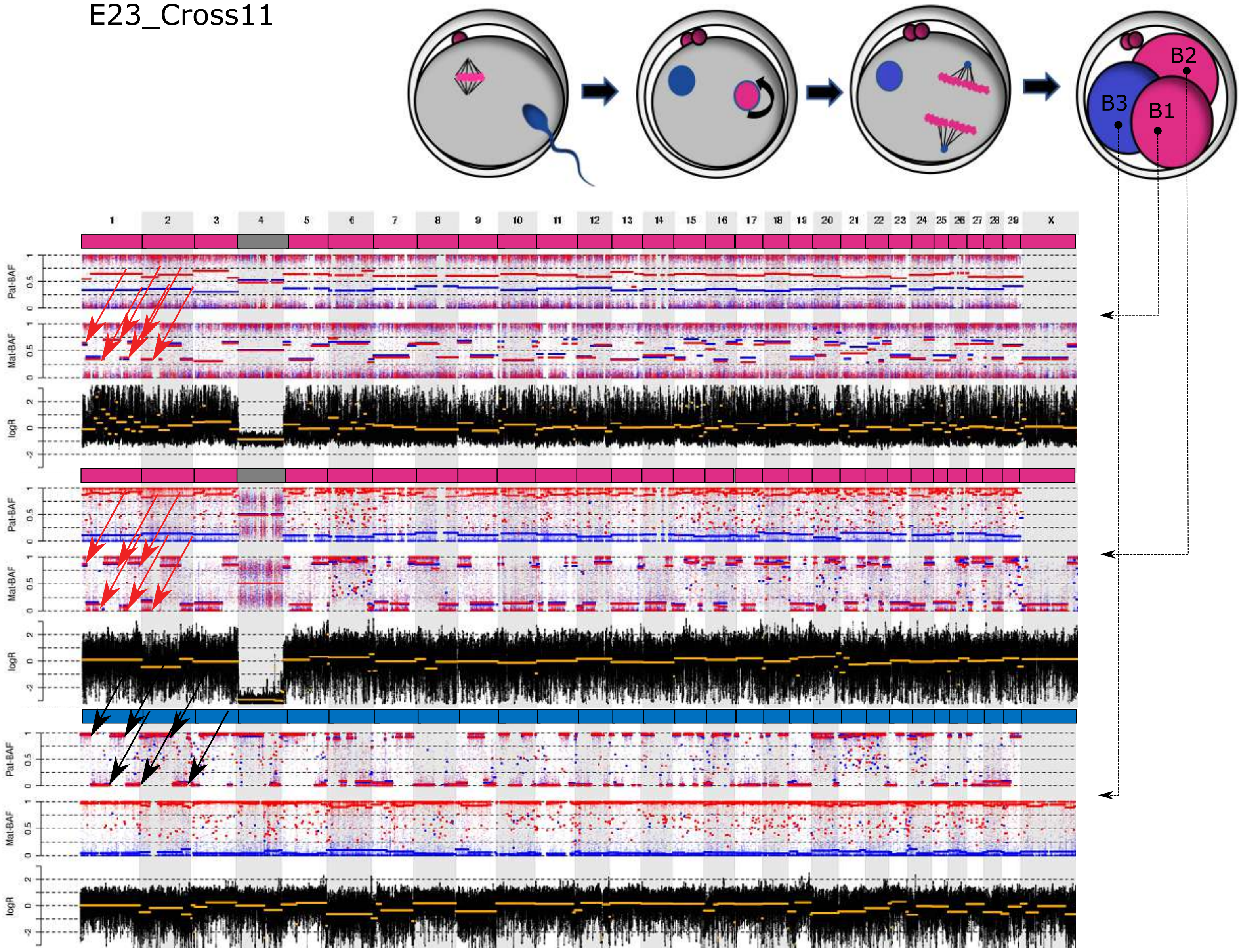

Figure S2B (continued)

E25_Cross12

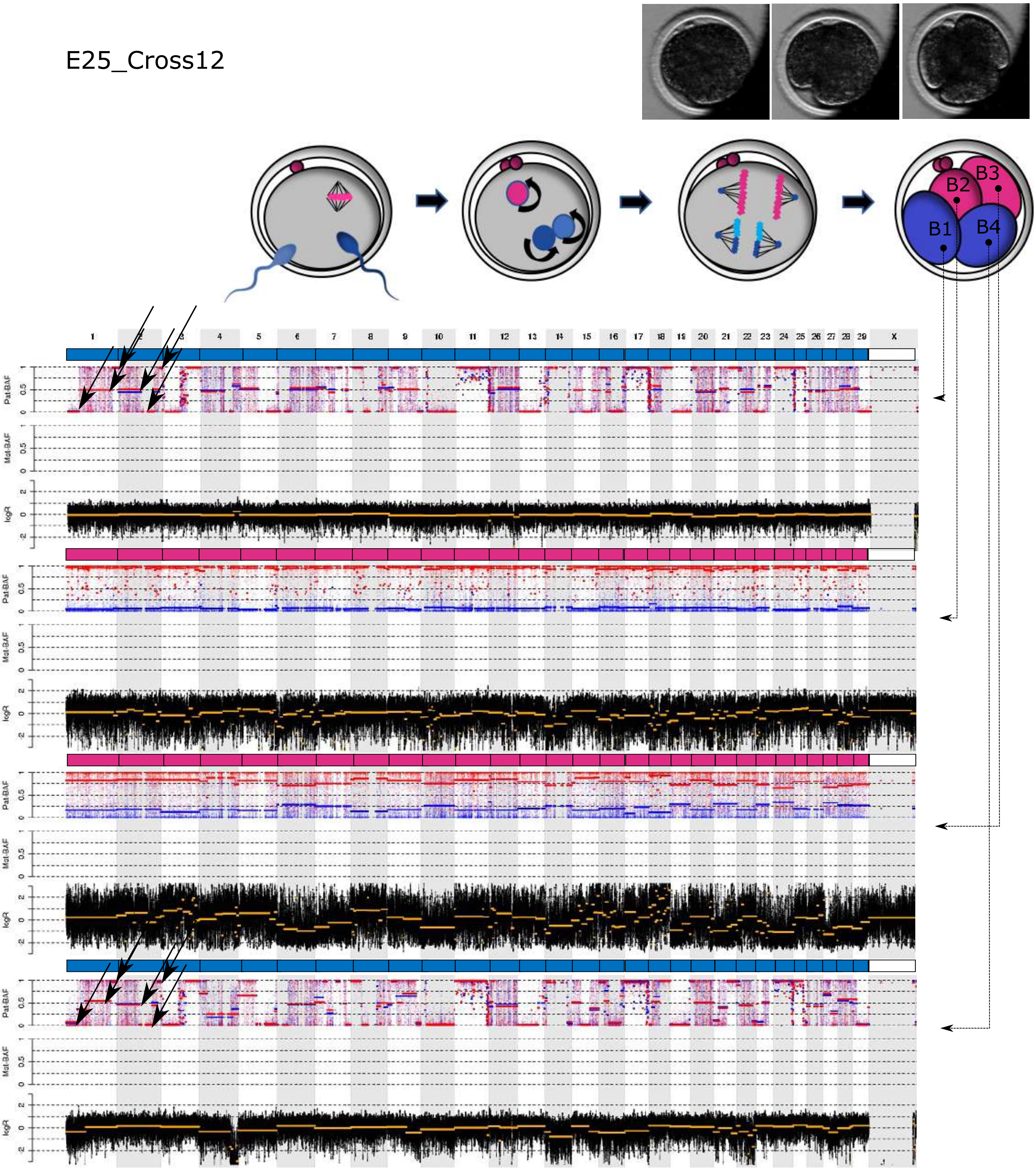

Figure S2B (continued)

4. Androgenetic embryos

E01_Cross01

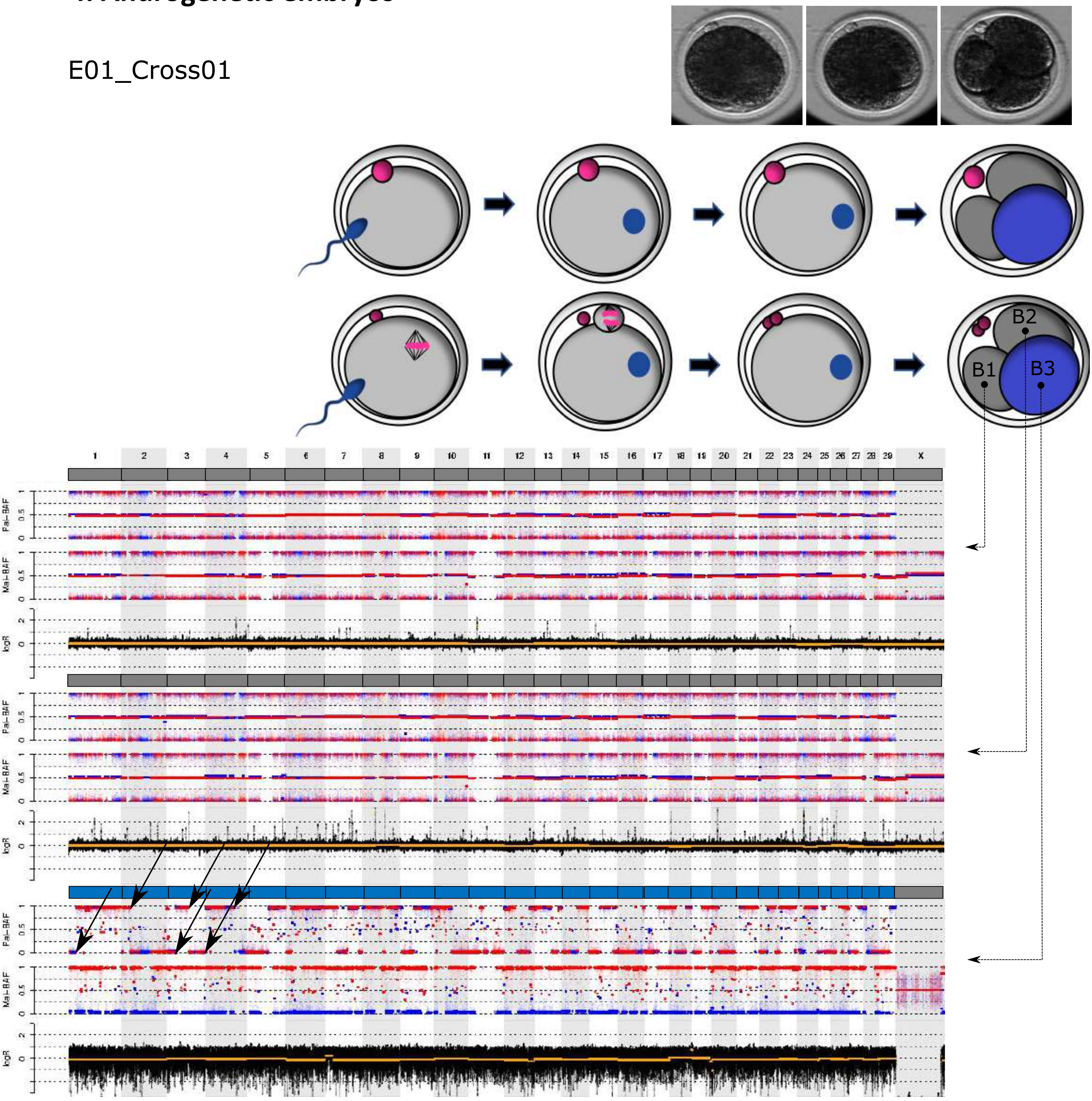

Figure S2B (continued)

E10_Cross05

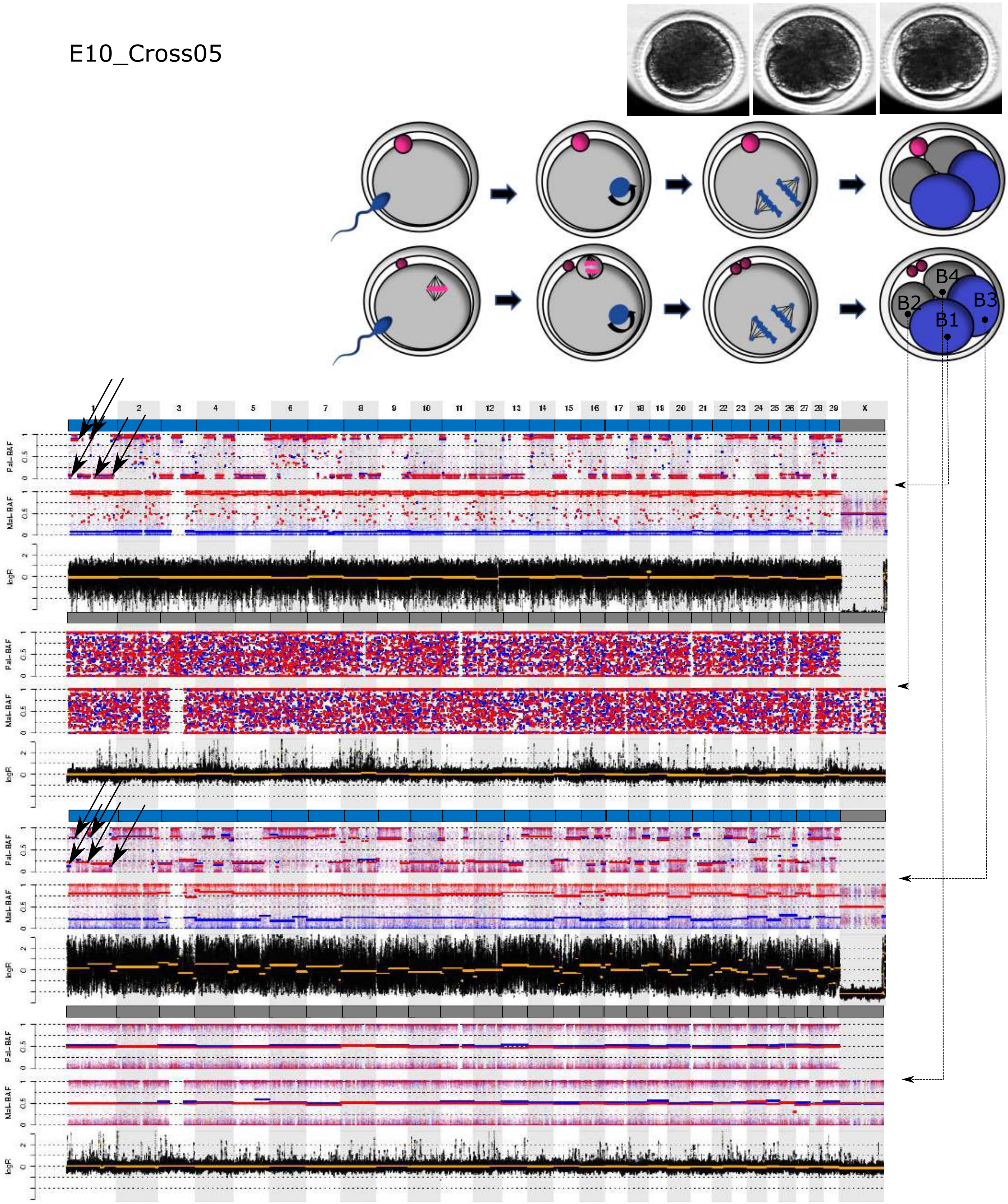

Figure S2B (continued)

5. Polyploid embryos

Some embryos harbored complexities additional to those discussed in the main text. In E03, reciprocal aneuploidy resulted in diandric trisomies in one blastomere and tetra-andric hexasomies in the other. As the diandric trisomic regions contained two identical paternal haplotypes, polyspermic fertilization followed by two replications of one of the paternal genomes is thought to have caused the presence of two copies of the same paternal genome and one copy of a different paternal genome. Additional cross-over sites in the tetra-andric pentasomic regions pointed towards the presence of an additional paternal genome in the third blastomere. A combination of replication and segregation errors in two of the three paternal genomes might have caused the complex embryonic profile.

Figure S2B (continued)

E03_Cross03

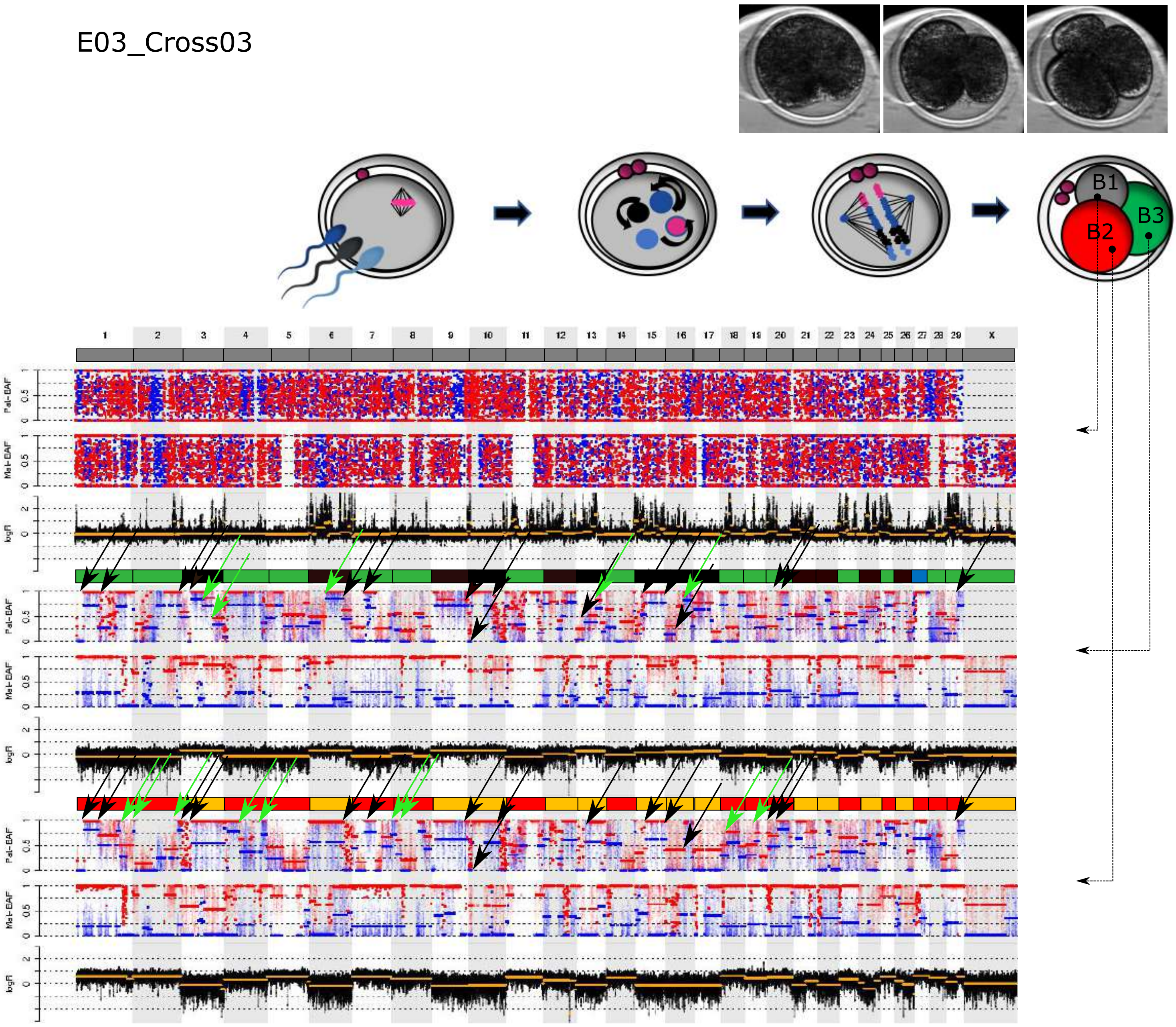

Figure S2B (continued)

E08_Cross04

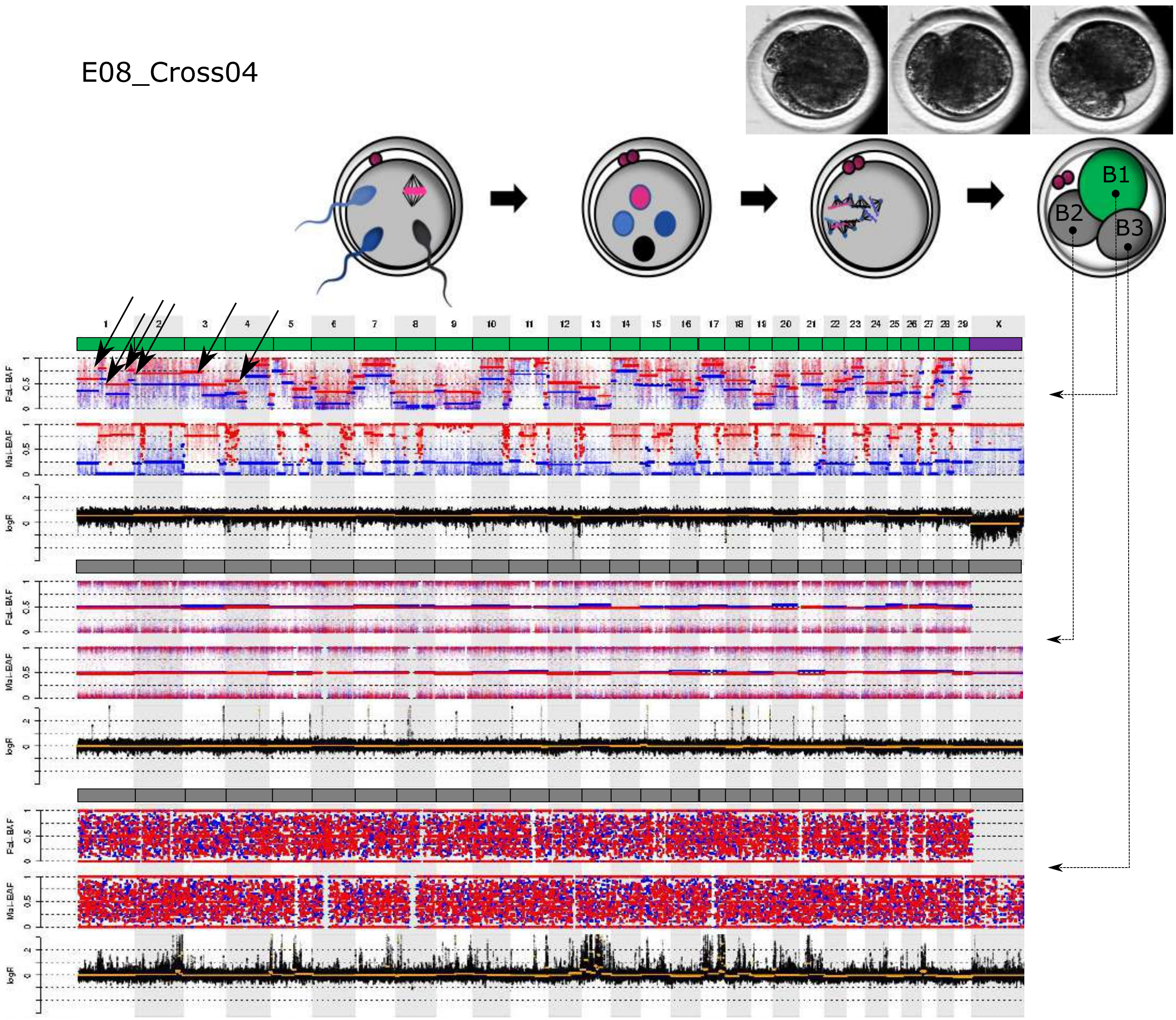

Figure S2B (continued)

E24_Cross12

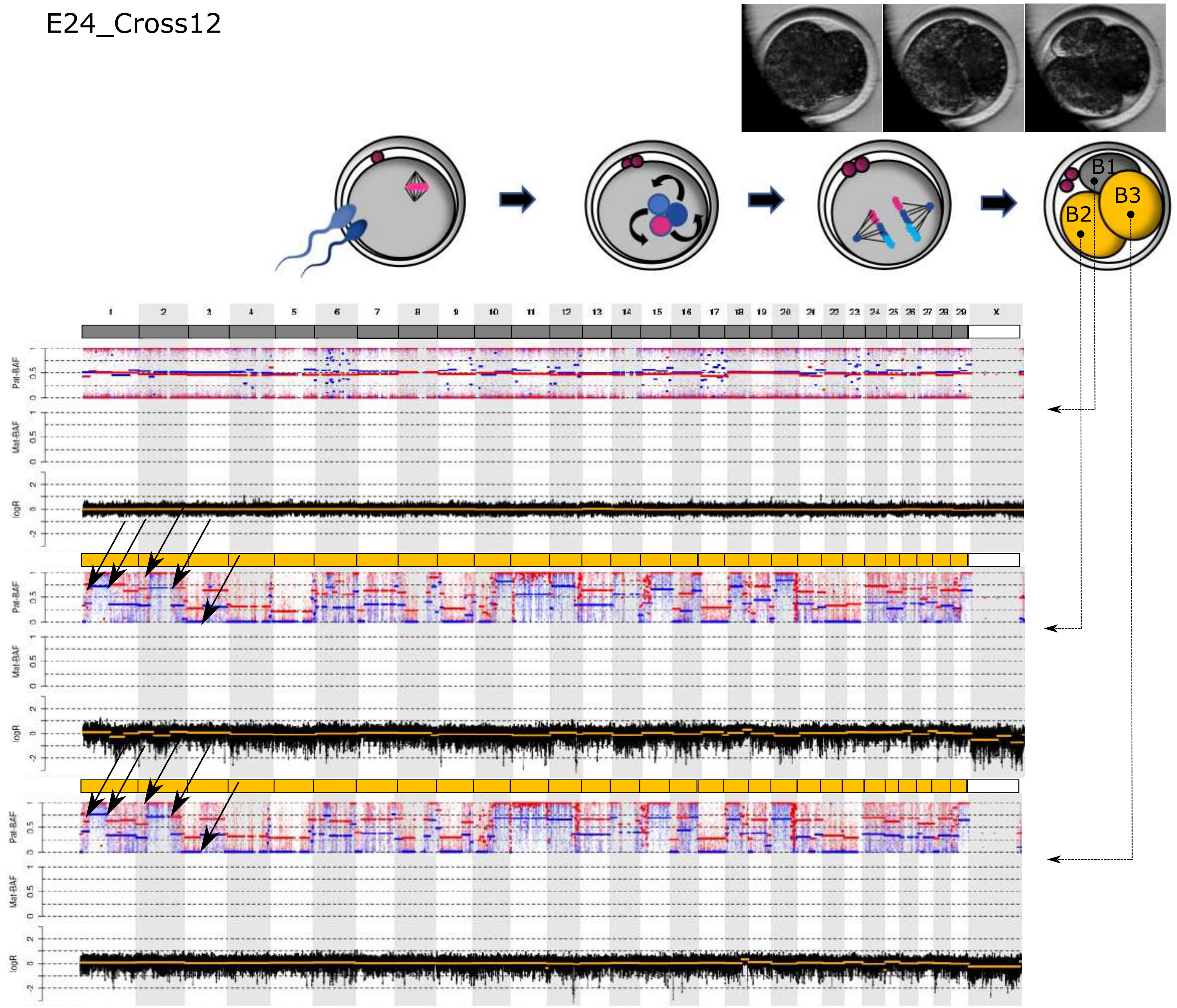

Figure S2B (continued)

6. Other profiles

Embryo E04, with two nuclear and one anuclear blastomere, was fertilized by one sperm. In the blastomeres containing DNA, a digynic triploid profile was likely a result of an erroneous first meiotic division due to the non-extrusion of DNA in the polar body or the reabsorption of the first polar body in the zygote. Visualization of two polar bodies in the time-lapse video indicated that non-extrusion of the maternal genome into the polar body was more likely. In addition, complex, non-reciprocal maternal and paternal chromosomal losses were observed throughout the genome of one blastomere resulting in diploid biparental chromosomes, maternal heterodisomy and monosomies of maternal or paternal origin. Those errors may have originated from errors during the first mitotic division.

Embryo E20 presented a complex mosaic chromosome profile. Three blastomeres contained the GW presence of multiple, seemingly random chromosome aneuploidies. Alternating (segmental) paternal disomies and maternal monosomies or disomies were found in a first blastomere, and alternating biparental chromosomes and maternal monosomies were found throughout the genome of a second blastomere. A third blastomere showed an androgenetic profile with segmental losses throughout the genome. Different recombination patterns in the regions where a paternal haplotype was simultaneously present in different blastomeres pointed towards dispermic fertilization. The profile would be consistent with the replication of three parental genomes followed by a tripolar spindle segregating the chromosomes randomly in three blastomeres.

Figure S2B (continued)

E04_Cross02

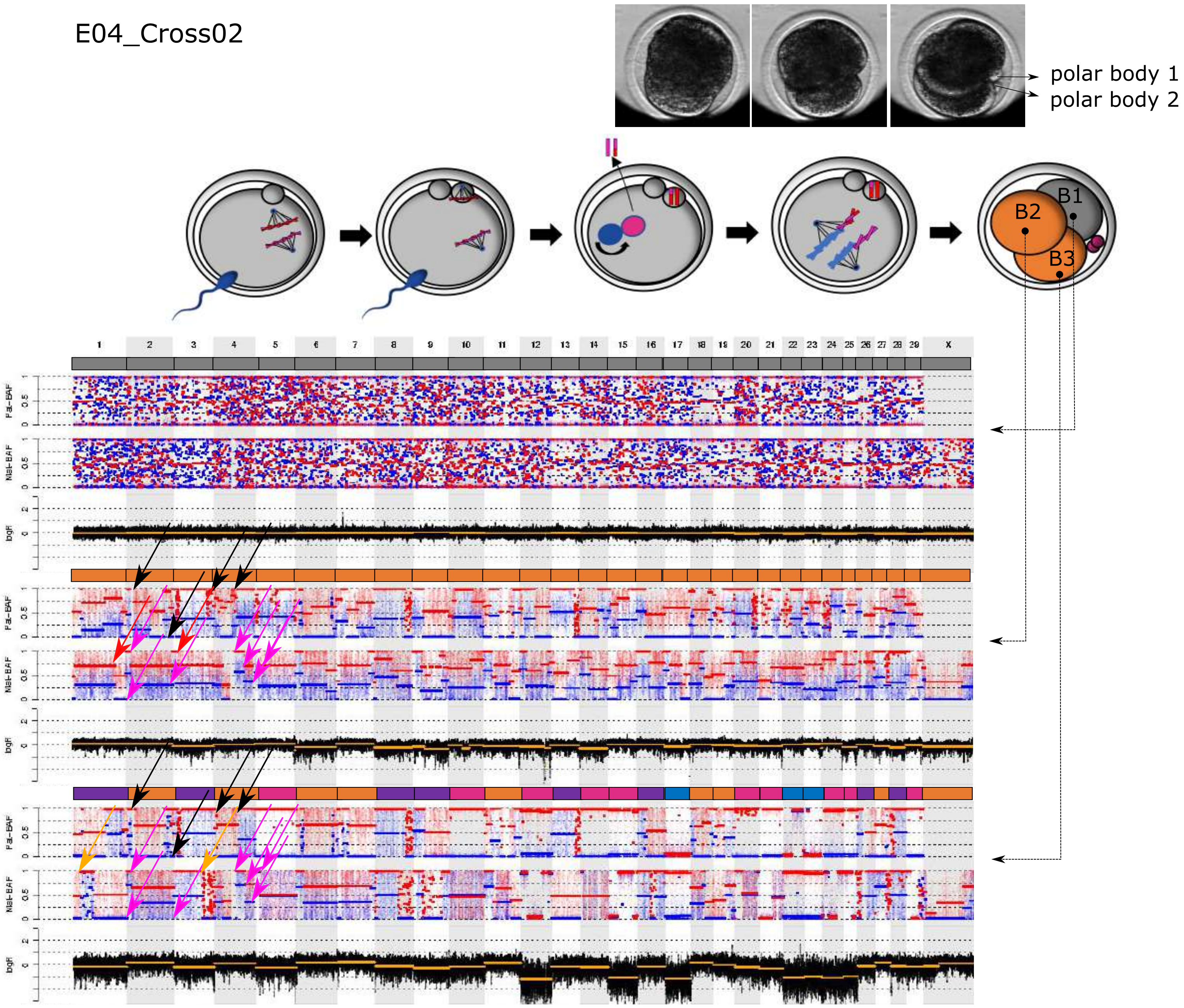

Figure S2B (continued)

E20_Cross08

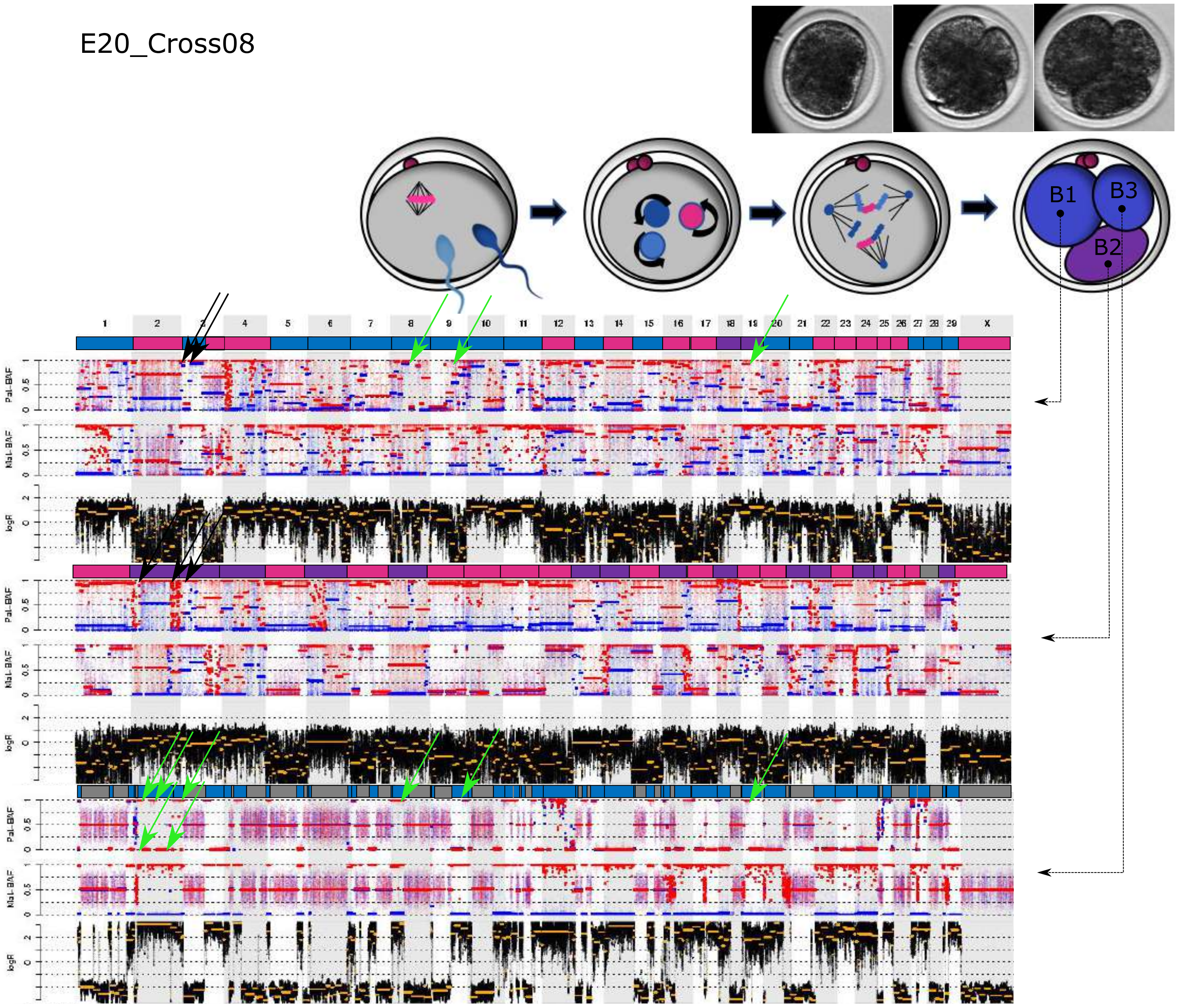

Supplement: Supplementary file 2 — Additional file 2: Figure S2. Analysis of blastomeres following multipolar zygotic division. A) Interpretation of haplarithm plots. Overview of chromosome-wise haplarithm patterns for distinct genomic constitutions (i.e. biparental disomy, paternal monosomy and paternal meiotic/dispermic uniparental heterodisomy). Corresponding whole-genome errors (i.e. biparental diploid or androgenetic) are characterized by the manifestation of those patterns throughout the (majority of the) genome. Defined single-cell BAF values of the segmented P1, P2, M1 and M2, form haplotype blocks, demarcated by pairwise breakpoints, i.e., homologous recombinations. Haplotype blocks, as well as the distance between the P1-P2 or M1-M2 in the paternal and maternal haplarithm, respectively, and the positioning of homologous recombinations, denote the origin and nature of copy number. The normalized LogR- values are integrated with haplarithm profiles for copy number profiling. Principles of interpretation are according to [64]. B) An overview of haplarithm profiles of 82 blastomeres and two fragments (grey squares) is depicted per category of whole-genome segregation profiles, as discussed in the main text. Each embryo is identified by a description at the top left of the embryo ID and cross (EmbryoID_Embryocross). At the top right, three chronological time-lapse images of the cleaving zygote are depicted. From left to right, the pictures show the initiation of the cleavage furrow, the ongoing first division and the embryo immediately after cleavage and before cell isolation (when video available). For each embryo, a schematic representation of likely steps leading to the genomic profile of each blastomere (B1-B4) or fragment (F1) is given. Chromosome-wise interpretation (1 - X) per blastomere is visualized in the bar above the haplarithm plots (see legend). Below each bar, the paternal haplarithm (pat-BAF), the maternal haplarithm (mat-BAF) and the normalized LogR values (LogR) are depicted. P [file 13059_2022_2763_MOESM2_ESM.pdf]
