# Supplementary material for: Parental genomes segregate into distinct blastomeres during multipolar zygotic divisions leading to mixoploid and chimeric blastocysts
Source: Genome Biol. 2022 Oct 3;23:201. doi: 10.1186/s13059-022-02763-2 (PMC9528162; doi:10.1186/s13059-022-02763-2)
Supplement: Supplementary file 8 — Additional file 8: Figure S8. Anuclear blastomeres and fragments. A) Phylogenetic tree based on reassembled mitochondrial genomes of 11 anuclear blastomeres and two anuclear fragments, showing a common ancestor for sequenced blastomeres and fragments retrieved from the same embryo. One anuclear blastomere was not included. B) Box plots and median values of the number of raw reads per 10 Mb bin (y-axis) per chromosome (chr1 - chrX) and for the mitochondrial DNA (chrM) (x-axis) as determined by single-cell low-coverage whole-genome sequencing for 12 anuclear blastomeres and two anuclear fragments demonstrate the abundance of mitochondrial DNA. C) Identical plots as in B, excluding chr M demonstrate the presence of fragments of chromosomal fragments in three anuclear blastomeres. D) Overlay of bright field and Hoechst fluorescent image show anuclear (1) and mononucleated (2;3) blastomeres resulting from a multipolar zygotic division in three blastomeres. [file 13059_2022_2763_MOESM8_ESM.pdf]

Figure S8

Anuclear blastomeres and fragments. A) Phylogenetic tree based on reassembled mitochondrial genomes of 11 anuclear blastomeres and two anuclear fragments, showing a common ancestor for sequenced blastomeres and fragments retrieved from the same embryo. One anuclear blastomere was not included. **B)** Box plots and median values of the number of raw reads per 10 Mb bin (y-axis) per chromosome (chr1 - chrX) and for the mitochondrial DNA (chrM) (x-axis) as determined by single-cell low-coverage whole-genome sequencing for 12 anuclear blastomeres and two anuclear fragments demonstrate the abundance of mitochondrial DNA. **C)** Identical plots as in B, excluding chr M demonstrate the presence of fragments of chromosomal fragments in three anuclear blastomeres. **D)** Overlay of bright field and Hoechst fluorescent image show anuclear (1) and mononucleated (2;3) blastomeres resulting from a multipolar zygotic division in three blastomeres.

Figure S8

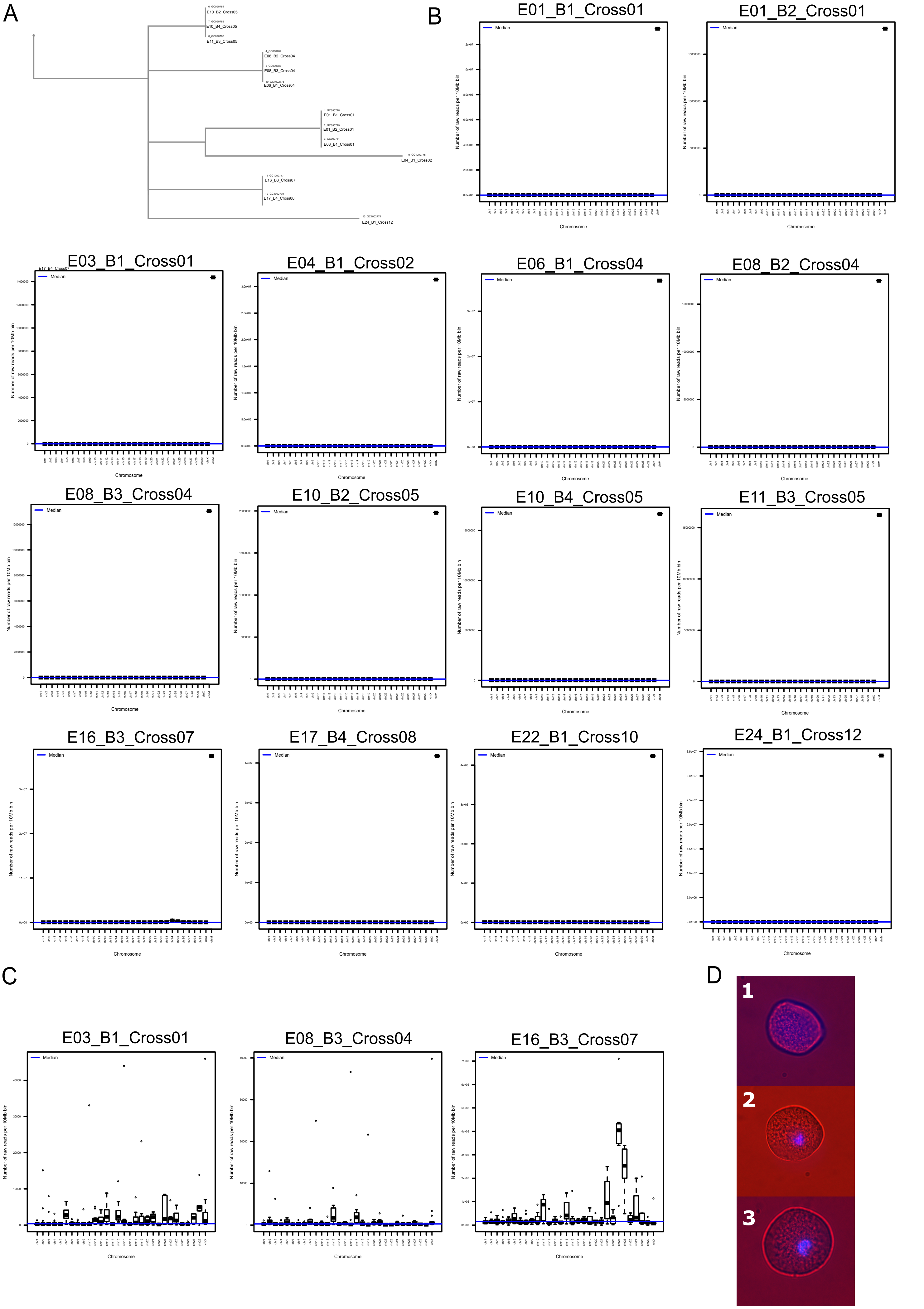
A
E10_B2_Cross05
E10_B4_Cross05
E11_B3_Cross05
E08_B2_Cross04
E08_B3_Cross04
E06_B1_Cross04
E01_B1_Cross01
E01_B2_Cross01
E03_B1_Cross01
E04_B1_Cross02
E16_B3_Cross07
E17_B4_Cross08
E24_B1_Cross12
B
E01_B1_Cross01
E01_B2_Cross01
E03_B1_Cross01
E04_B1_Cross02
E06_B1_Cross04
E08_B2_Cross04
E08_B3_Cross04
E10_B2_Cross05
E10_B4_Cross05
E11_B3_Cross05
E16_B3_Cross07
E17_B4_Cross08
E22_B1_Cross10
E24_B1_Cross12
Median
Number of raw reads per 10Mb bin
Chromosome
C
E03_B1_Cross01
E08_B3_Cross04
E16_B3_Cross07
D
1
2
3
