# Supplementary material for: Parental genomes segregate into distinct blastomeres during multipolar zygotic divisions leading to mixoploid and chimeric blastocysts
Source: Genome Biol. 2022 Oct 3;23:201. doi: 10.1186/s13059-022-02763-2 (PMC9528162; doi:10.1186/s13059-022-02763-2)
Supplement: Supplementary file 9 — Additional file 9: Figure S9. Genome-wide composition of blastocysts following multipolar zygotic division. Circos plots of six embryos that developed to the blastocyst stage following multipolar zygotic division in which each circle represents the interpreted genome constitution per chromosome (1 - X) of a sampled single cell. The interpreted genome spanning the largest part of the chromosome was chosen as overall interpretation per chromosome, as such, segmental chromosomal errors are not depicted. On the left of each circus plot, a picture is shown of each embryo before dissociation (not available for E26_Cross13). [file 13059_2022_2763_MOESM9_ESM.pdf]

Figure S9

Genome-wide composition of blastocysts following multipolar zygotic division. Circos plots of six embryos that developed to the blastocyst-stage following multipolar zygotic division in which each circle represents the interpreted genome constitution per chromosome (1 - X) of a sampled single cell. The interpreted genome spanning the largest part of the chromosome was chosen as overall interpretation per chromosome, as such, segmental chromosomal errors are not depicted. On the left of each circus plot, a picture is shown of each embryo before dissociation (not available for E26_Cross13).

Figure S9

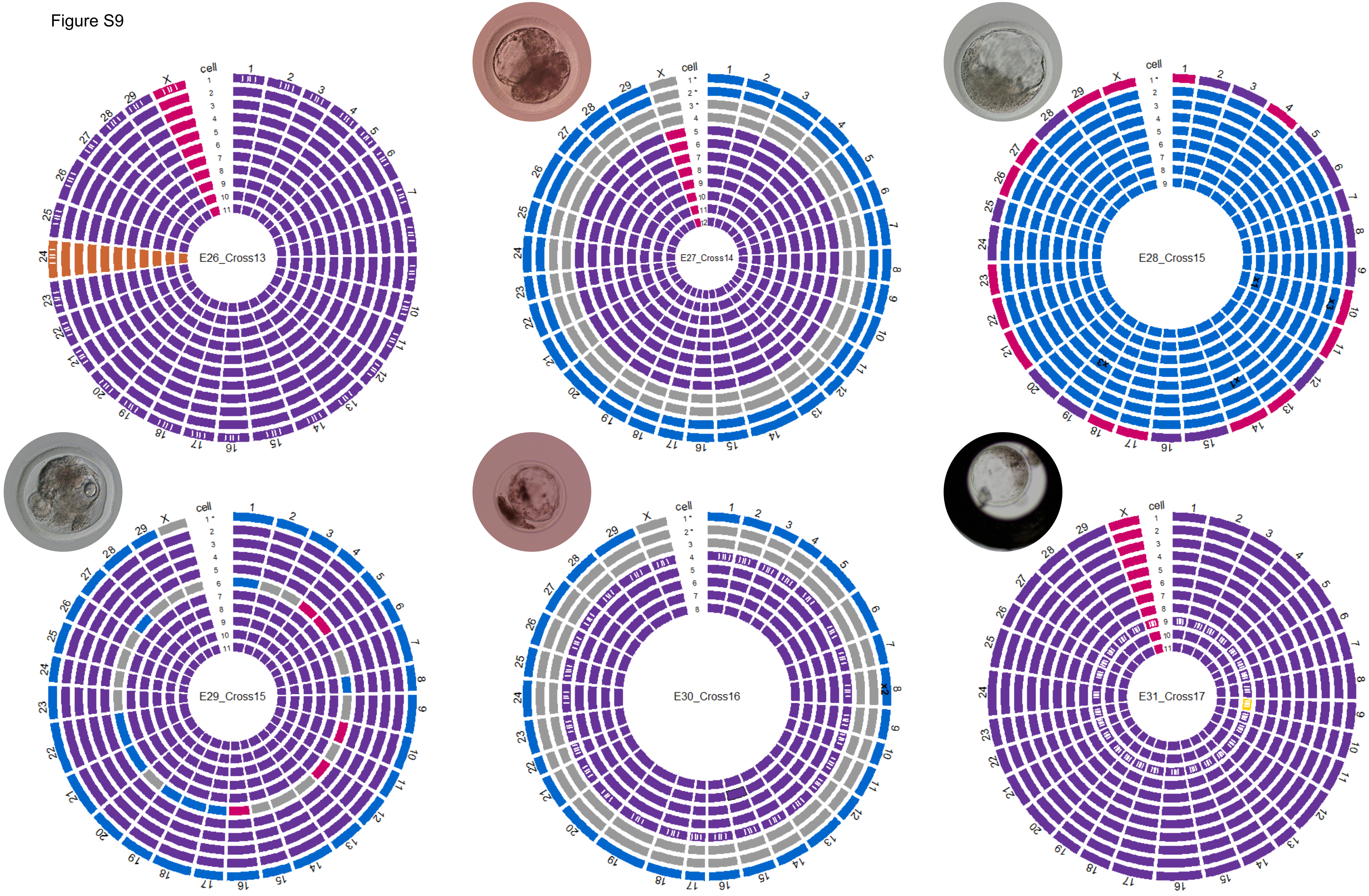

biparental disomy · paternal only · maternal only · digynic trisomy · diandric trisomy · triandric tetrasomy · nullisomy

low quality, presumed biparental disomy · low quality, presumed maternal · low quality, presumed digynic trisomy · low quality, presumed diandric trisomy

* blastomere extruded to perivitteline space **x1** uniparental chromosomal loss **x2/x3** uniparental chromosomal gain

Figure S9 (continued)

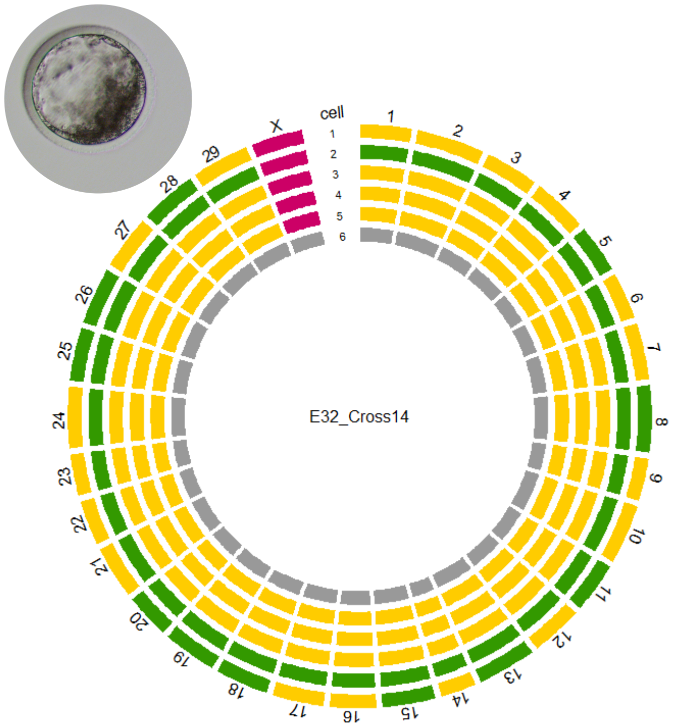

biparental disomy paternal only maternal only digynic trisomy diandric trisomy triandric tetrasomy nullisomy

low quality, presumed biparental disomy low quality, presumed maternal low quality, presumed digynic trisomy low quality, presumed diandric trisomy

* blastomere extruded to perivitteline space **x1** uniparental chromosomal loss **x2/x3** uniparental chromosomal gain
