# Supplementary material for: Parental genomes segregate into distinct blastomeres during multipolar zygotic divisions leading to mixoploid and chimeric blastocysts
Source: Genome Biol. 2022 Oct 3;23:201. doi: 10.1186/s13059-022-02763-2 (PMC9528162; doi:10.1186/s13059-022-02763-2)
Supplement: Supplementary file 10 — Additional file 10. Review history. [file 13059_2022_2763_MOESM10_ESM.docx]

Review History

**First round of review**

**Reviewer 1**

**Are you able to assess all statistics in the manuscript, including the appropriateness of statistical tests used?** Yes, and I have assessed the statistics in my report.

**Comments to author:**

Tine de Coster and colleagues hand in a combination of systematic experimental observations of early stage mammalian embryos and interpretations allowing conclusions about novel mechanisms of early cell divisions. In particular, they combine whole genome analysis of human embryo samples with a more systematic study of individual blastomeres of in vitro fertilized bovine embryos, where more samples are readily accessible while still being a good model for human conception and development. Taken together, I find this a truly remarkable study that, from my point of view, certainly deserves publication in Genome Biology.

There are two general concerns/comments, which, as I am aware, are both not easy to address. Yet, from my point of view, addressing these would be a means to give the story a possibly final twist to become a hallmark paper.

1) The authors conclude throughout the story from their genomic data about spindle organization, i.e. the execution of chromosome separation / karyokinesis. Ideally, at least for some examples, the authors should directly visualize tripolar/tetrapolar/multipolar spindles e.g. in bovine embryos. Given the rather high incidence of ca. 1/3 zygotic division failures one would, even when observing a rather small sample size, expect to see aberrant phenotypes regularly.

2) The genome analysis focuses on situations derived from polyspermic fertilization in bovine embryos and subsequent zygotic multipolar division. I think it would be evenly interesting and important to analyze more systematically what happens in the majority of embryos with an initial "normal" bipolar zygotic division. Is the system (as discussed and suggested by many other studies) error prone even after fertilization, which may then lead to mixoploid blastomeres despite "regular" fertilization, zygotic division and development onset. Or, alternatively, are genome wide aberrations in individual cells hardly/not at all observed when zygotic division happens normally. Either one would significantly sharpen the conclusions.
I therefore could image that a, possibly smaller, experimental sample form regularly dividing embryos would nicely add-up to the existing data and would allow more experimentally documented conclusions.

Formal issues:

The abstract is unnecessarily long and therefore not ideally suited to clearly pinpoint the main conclusions of the paper. Experimental details may be reduced to better peel out the main points….
.. similarly… is the discussion lengthy and speculates about too many different scenarios of mis-segregation. I suggest bundling (at least some) observations and try to generalize, quicker leading to statements about the overall novel cell biological and molecular principles.

The authors use samples of different origin (human, bovine) for good reasons. It should be more clearly stated, however, when and why they change from one system to the other.

Minor issues:

Some numbers are not clear to me: 387 embryos started, of which 88,9% (344) cleaved. Of these, 35,2% (121) displayed three or more blastomeres. In the next sentence, 25 are further analyzed. Part of the 121? Randomly chosen, or by any rational ?

Colors in Fig. 1 discriminating biparental unbalanced and maternal only are hard to separate, it may be easier with "more" different colors. Btw: "biparental unbalanced" is only found directly in the figure but neither in the legend nor in the results section.

Clarify, or better consistently use, paternal/maternal monosomy and paternal/maternal only.

Fig. 4 legend: "Likely events leading to the segregation…" sounds somewhat vague. It may be better to say "Schematic drawing of procedures that best explain the segregation…"

Fig. 6, legend: please assign A,B,C of the figure also in the legend.

**Reviewer 2**

**Are you able to assess all statistics in the manuscript, including the appropriateness of statistical tests used?** Yes, and I have assessed the statistics in my report.

**Comments to author:**

In this study, the authors combine genetic testing data from multiple individual cells of human and bovine embryos to demonstrate the existence and surprising prevalence of mixoploidy (embryos with mosaic ploidies) and/or chimerism (mosaic embryos with multiple cell lineages that trace ancestry to different sperm). While such abnormalities are enriched among arrested embryos, a subset of affected embryos actually survive to the blastocyst stage. Polyspermic fertilization appears to be the predominant cause. Notably, the detection of mosaic ploidies require the use of haplotype-based methods, as conventional approaches to PGT-A often miss these phenomena due to technical limitations.

Broadly, I found this to be a convincing and important study that applies detailed haplotype-based analysis to a large sample of individual cells to offer a unique view on early development. The (albeit few) statistical analyses are appropriate. I have a few broad comments on the impact/interpretation of the work, as well as some stylistic suggestions and conceptual questions that I hope will be addressed.

Major comments

1) The sample of human embryos (n=2) makes it challenging to draw strong conclusions at this stage, but it is at least suggestive that the same error mechanisms that operate in bovine may also operate in humans.

2) Related to the point above, the authors demonstrated that polyspermic fertilization is the main driver of chimerism/mixoploidy in bovine embryos. How common is polyspermy in human IVF (which if I understand correctly, mostly uses ICSI)? How common is polyspermy in human natural conception? Is there any reason to believe that polyspermy rates would differ between human and bovine? The answers to these questions would offer crucial context for how much mixoploidy/chimerism are contributing to pregnancy loss in human IVF and natural conception. If polyspermy is not extremely common, then I don't see how the authors can make claims such as the one that "heterogoneic division" is a major contributor to low human fertility

3) The figures are extremely dense--especially those displaying the raw BAFs. I think that drastically simplifying all of the figures would help expand the potential audience for this work, without sacrificing much in regard to the overall message.

4) The flow of the text is similarly challenging, as it reads as a very long list of detailed case studies of individual embryos, where hypothetical mechanisms of chimerism/mixploidy formation are deduced from the haplotype patterns of individual embryos (or small sets of embryos). It would greatly assist readers if the authors would do more to synthesize their findings and provide more concise descriptions of the various error modes. As written, these detailed descriptions of particular mechanisms of error feel somewhat esoteric, written for a narrow specialist audience. It would help, for example, to tally and compare how many embryos fall into the various error categories described. Are some much more common than others? Are some more strongly associated with embryonic arrest? This would help frame the work as more than a series of anecdotes and emphasize the bigger picture. These concerns apply both to the Results and Discussion, which should be made more concise if the hope is to appeal to a broad readership.

5) The comments that the findings "challenge cell biological dogmas" are overstatements, in my opinion. These are simply errors in the canonical processes of cell division and chromosome segregation.

Minor comments:

1) line 54: suggest changing "man" to "humans"

2) line 354: "In this study, we demonstrate for the first time that genome-wide mosaicism exists in human
preimplantation embryos" is also an overstatement in my opinion. Previous studies, including by these authors, have offered at least indirect evidence of mixploidy in humans (see references 78-89 of current paper, for example). Is the distinction simply that the authors see it in _preimplantation_ embryos?

3) line 396: "gen" -> "gene"

**Authors Response**

**When revising the manuscript, please also ensure that the manuscript is formatted according to our instructions and that editable figures are provided with the revised manuscript. Please see:**

[**https://eur03.safelinks.protection.outlook.com/?url=https%3A%2F%2Fgenomebiology.biomedcentral.com%2Fsubmission-guidelines%2Fpreparing-your-manuscript%2Fresearch&amp;data=05%7C01%7Ctine.decoster%40ugent.be%7C243453df60c04ea342b908da275f045b%7Cd7811cdeecef496c8f91a1786241b99c%7C1%7C0%7C637865587341383258%7CUnknown%7CTWFpbGZsb3d8eyJWIjoiMC4wLjAwMDAiLCJQIjoiV2luMzIiLCJBTiI6Ik1haWwiLCJXVCI6Mn0%3D%7C3000%7C%7C%7C&amp;sdata=MPGqFP65ZHRTA%2BrNaHiM9WAb0VTHfd7s17w2%2FymkAvs%3D&amp;reserved=0**](https://eur03.safelinks.protection.outlook.com/?url=https%3A%2F%2Fgenomebiology.biomedcentral.com%2Fsubmission-guidelines%2Fpreparing-your-manuscript%2Fresearch&amp;data=05%7C01%7Ctine.decoster%40ugent.be%7C243453df60c04ea342b908da275f045b%7Cd7811cdeecef496c8f91a1786241b99c%7C1%7C0%7C637865587341383258%7CUnknown%7CTWFpbGZsb3d8eyJWIjoiMC4wLjAwMDAiLCJQIjoiV2luMzIiLCJBTiI6Ik1haWwiLCJXVCI6Mn0%3D%7C3000%7C%7C%7C&amp;sdata=MPGqFP65ZHRTA%2BrNaHiM9WAb0VTHfd7s17w2%2FymkAvs%3D&amp;reserved=0) **for further details.**

Authors: We have provided separate figure files for the figures in .pdf or .tif format, according to the instructions provided in the web link. For the sake of readability, we have kept the figures also within the revised manuscript.

In the revised manuscript, Fig. 3 has been added as a new figure (Fig. 3_new.tif) and Fig. 1, 4 and 6 have been updated (Fig. 1_update.pdf, Fig. 4_update.pdf and Fig. 6_update.pdf.) Also, the additional files have been updated (Additional files_update_compressed.pdf).

**We also require all data to be deposited to a relevant repository prior to resubmission, although you may supply a reviewer access token if desired. Please note that if we decide to publish your manuscript we will require that all data be made publicly accessible and the 'live' accession numbers included in a separate Availability of Data and Materials section of the manuscript.**

Authors: Human embryo, parental and phasing relatives’ raw genotyping data has been deposited at the European Genome-phenome Archive (EGA), which is hosted by the EBI and the CRG, under accession number EGAS00001005543. It is available to academic users upon request to the Data Access Committee (DAC) of KU Leuven via the corresponding author (J.R.V). In compliance with the GDPR (General Data Protection Regulation 2016/679) and the study protocol, the human PGT-M blastocyst dataset used in the study is not publicly available. The bovine sequencing data for this study have been deposited in the European Nucleotide Archive (ENA) and are accessible at EMBL-EBI under accession number PRJEB46925 (https://www.ebi.ac.uk/ena/browser/view/PRJEB46925). The bovine SNP data have been deposited in NCBI's Gene Expression Omnibus and are accessible through GEO Series accession number GSE182345 (https://www.ncbi.nlm.nih.gov/geo/query/acc.cgi?acc= GSE182345). The live imaging data have been deposited by the research data repository DataverseNL (dataverse.nl) (https://doi.org/10.34894/AQ9OIU).

**Reviewer #1 comments:**

**Tine de Coster and colleagues hand in a combination of systematic experimental observations of early stage mammalian embryos and interpretations allowing conclusions about novel mechanisms of early cell divisions. In particular, they combine whole genome analysis of human embryo samples with a more systematic study of individual blastomeres of *in vitro* fertilized bovine embryos, where more samples are readily accessible while still being a good model for human conception and development. Taken together, I find this a truly remarkable study that, from my point of view, certainly deserves publication in Genome Biology.**

**There are two general concerns/comments, which, as I am aware, are both not easy to address. Yet, from my point of view, addressing these would be a means to give the story a possibly final twist to become a hallmark paper.**

**The authors conclude throughout the story from their genomic data about spindle organization, i.e. the execution of chromosome separation / karyokinesis. Ideally, at least for some examples, the authors should directly visualize tripolar/tetrapolar/multipolar spindles e.g. in bovine embryos. Given the rather high incidence of ca. 1/3 zygotic division failures one would, even when observing a rather small sample size, expect to see aberrant phenotypes regularly.**

Authors: We agree that videos would strengthen the manuscript. Hence, we performed a live-imaging experiment on 55 bovine embryos. Four of these embryos divided in 3 or 4 blastomeres. Therefore we included these four, illustrating the extrusion of “empty blastomeres”, the formation of private parental spindles both in normally fertilized and polyspermic embryos, the formation of additional paternal spindles and the extrusion of an additional paternal genome (Abstract: line 30-31; Background: line 111-113; Results: line 148-152, 182-216, 231-233; 331-336, 360-365; Discussion: line 477-478, 557-562, 622-626, 647-649, 701-706; Methods: line 751-752, 784-819; Availability of data and materials: line 952-953; Author contributions: line 972-973; Acknowledgements: 977-988; Additional files: line 1361-1376). Timed snapshots of the videos have been added in Fig. 3 (Fig.3_new.tif). The videos can be retrieved in Additional Movie S2-6 (Movie S2_3A, Movie S3_3B, Movie S4_3C, Movie S5_3D, Movie S6_3E).

**The genome analysis focuses on situations derived from polyspermic fertilization in bovine embryos and subsequent zygotic multipolar division. I think it would be evenly interesting and important to analyze more systematically what happens in the majority of embryos with an initial "normal" bipolar zygotic division. Is the system (as discussed and suggested by many other studies) error prone even after fertilization, which may then lead to mixoploid blastomeres despite "regular" fertilization, zygotic division and development onset. Or, alternatively, are genome wide aberrations in individual cells hardly/not at all observed when zygotic division happens normally. Either one would significantly sharpen the conclusions.**

**I therefore could image that a, possibly smaller, experimental sample form regularly dividing embryos would nicely add-up to the existing data and would allow more experimentally documented conclusions.**

Authors: Thank you for this question. The aim of this study was to provide direct evidence of heterogoneic cell division. To test our hypothesis, we therefore focused this study on the analysis of embryos undergoing a multipolar zygotic division. Initially, we aimed to also analyze an additional cohort of blastomeres from selected, normally fertilized embryos presenting with a multipolar division, to explore whether heterogoneic cell division might occur in normally fertilized oocytes. Yet, selecting normally fertilized embryos containing 2 PN in bovine embryos was impossible, due to the lipid-rich cytoplasm. In our recent publication, where we analyzed spindle assembly in 1421 fixed and 21 live-imaged bovine zygotes, we observed failure of the parental spindles to merge in the 19% of 2PN zygotes. In the live-imaged bovine zygotes where parental spindles failed to merge (n=2), heterogoneic cell division occurred while the zygote cleaved in two blastomeres only (Schneider *et al.* 2021). Therefore heterogoneic cell division seems to occur also in ‘regular’ fertilized eggs which cleave into two blastomeres.

We have included a reflection on the occurrence of heterogoneic following normal fertilization and bipolar zygotic division cell division (Discussion: line 544-561)

**The abstract is unnecessarily long and therefore not ideally suited to clearly pinpoint the main conclusions of the paper. Experimental details may be reduced to better peel out the main point.**

**Similarly… is the discussion lengthy and speculates about too many different scenarios of mis-segregation. I suggest bundling (at least some) observations and try to generalize, quicker leading to statements about the overall novel cell biological and molecular principles.**

Authors: we have adapted the abstract and the manuscript. Especially, in the abstract we highlighted the essence and reduced the methodological details in the second half. In the main text, we tried to make the text more understandable for a broad public, reduce the amount of detailed information and cut some repetitions.

Specifically, we moved details on some embryos within the section “Embryos with biparental and androgenetic blastomeres” (Results: line 337-352) and within the section “Polyploid embryos (Results: line 392-399) to Additional Figures S2B (Additional files_update_compressed.pdf, Fig. S2B). Similarly, we have moved the paragraph “other embryos”, describing genome-wide profiles and the likely causative events of two complex embryos which could not be categorized together with the other embryos (Results: line 404-421) to Additional Figures S2B (Additional files_update_compressed.pdf, Fig. S2B). In the discussion, we largely removed details on spindle formation in the different embryos, as the core information is covered in the result section and discussion line 578-598 (Discussion: line 598-628). Also, in the discussion, paragraph “Other whole genome segregation errors” was removed, as heterogoneic cell division is the main focus of our manuscript (Discussion: line 641-659).

We have altered the term “ectopic” paternal spindle to “additional” paternal spindle throughout the manuscript, because ectopic might point to an abnormal position of the spindle, which is not necessarily the case and therefore, might be confusing.

We used a multitude of terms for to describe the extrusion of a parental genome into a separate blastomere without a ‘spindle segregation’ such as: ejection, cytoplasmic protrusion etc. We changed it throughout the manuscript to the term ‘extrusion” of a paternal genome.

**The authors use samples of different origin (human, bovine) for good reasons. It should be more clearly stated, however, when and why they change from one system to the other.**

Authors: we specified this more clearly in throughout the manuscript (e.g. Abstract: line 26; Background: line 108, 112; Results: line 120, 147, 182; Discussion: line 473, 495).

**Minor issues:**

**Some numbers are not clear to me: 387 embryos started, of which 88,9% (344) cleaved. Of these, 35,2% (121) displayed three or more blastomeres. In the next sentence, 25 are further analyzed. Part of the 121? Randomly chosen, or by any rational?**

We clarified the rationale for embryo selection in the Material and Method section (line 777-782 and line 852-856). We further clarified the different sets by adding “For analysis of genomic profiles and additionally for staining purposes” and adding “a first and a set” after the initial description of the cytokinetic events in 387 embryos (Results: line 153, 158 and 246).

**Colors in Fig. 1 discriminating biparental unbalanced and maternal only are hard to separate, it may be easier with "more" different colors. Btw: "biparental unbalanced" is only found directly in the figure but neither in the legend nor in the results section.**

Authors: we clarified the results section (Results: line 125-131) and updated Fig. 1 legend and figure with a black color to represent genome-wide (pentagynic) hexasomies (Fig. 1_update.pdf). The black color was also chosen to represent the (penta-andric) hexasomies in bovine embryo E03_Cross03 of which the haplarithm plot can be retrieved in the Additional files (Additional files_update_compressed.pdf, Fig. S2B).

**Clarify, or better consistently use, paternal/maternal monosomy and paternal/maternal only.**

Authors: paternal or maternal only can have some different interpretations: maternal or paternal isodisomy, heterodisomy or monosomy (see Additional files_update_compressed.pdf, Fig. S2A: androgenetic and gynogenetic). In addition, haplarithm plots do not allow to discriminate between uniparental (maternal or paternal) isodisomy and monosomy, except when the LogR value of the specific chromosome diverges from 0 in the same or other chromosomes due to chromosomal errors. That is because the B-allele frequency values are identical in these types of errors and the LogR values calculated for the majority of SNPs are normalized to zero (for examples of identical plots of paternal/maternal monosomy and isodisomy, see Additional files_update_compressed.pdf, Fig. S2A). For these reasons, we decided to avoid the use of too many different colors in the figures by depicting chromosomes that have a full maternal content with a pink and chromosomes with a full paternal content with a blue colour. However, when a distinction could be made (on a chromosomal/genome-wide level) between uniparental isodisomy, heterodisomy or monosomy, due to the presence of heterodisomy or due to chromosomal errors, we have given the more correct name in the manuscript.

**Fig. 4 legend: "Likely events leading to the segregation…" sounds somewhat vague. It may be better to say "Schematic drawing of procedures that best explain the segregation…"**

Authors: we have adapted the text in the figure legend of Fig.4 (Results: line 307) and similarly, also the figure legend of Fig. 5 (Results: line 367).

**Fig. 6, legend: please assign A,B,C of the figure also in the legend.**

Authors: we have adapted the figure legend of what is now Fig. 7 accordingly (Discussion: line 708-709).

**Reviewer #2 comments:** **In this study, the authors combine genetic testing data from multiple individual cells of human and bovine embryos to demonstrate the existence and surprising prevalence of mixoploidy (embryos with mosaic ploidies) and/or chimerism (mosaic embryos with multiple cell lineages that trace ancestry to different sperm). While such abnormalities are enriched among arrested embryos, a subset of affected embryos actually survive to the blastocyst stage. Polyspermic fertilization appears to be the predominant cause. Notably, the detection of mosaic ploidies requires the use of haplotype-based methods, as conventional approaches to PGT-A often miss these phenomena due to technical limitations.**

**Broadly, I found this to be a convincing and important study that applies detailed haplotype-based analysis to a large sample of individual cells to offer a unique view on early development. The (albeit few) statistical analyses are appropriate. I have a few broad comments on the impact/interpretation of the work, as well as some stylistic suggestions and conceptual questions that I hope will be addressed.**

**Major comments**

**1) The sample of human embryos (n=2) makes it challenging to draw strong conclusions at this stage, but it is at least suggestive that the same error mechanisms that operate in bovine may also operate in humans.**

Authors: We do agree that those findings suggest the same error mechanism to operate in humans and we agree that having human embryos analyzed following a tripolar zygotic division to prove that identical genome-wide segregation errors operate in human would make a valuable addition and we hope to perform such analysis in the future. Due to ethical, legislative and practical issues concerning the generation and use of human embryos, we were unable to set up a prospective analysis in human embryos. Hence, we resorted to clinically asserted embryos and the analysis of human blastocysts that were considered non-eligible for transfer to dissociate single-cells from.

The embryos used here do demonstrate the existence of distinct parental cell lineages in human embryos and were identified as a consequence of haplotype-based screening of cleavage embryos, using abnormal haplotyped embryos for a full dissection. Since haplotype based diagnostic embryo screening is only performed in less than a handful of clinical diagnostic laboratories, those phenomena have never been recognized and show that haplotype-based analyses of embryos will/would be important and will further improve embryo selection.

**2) Related to the point above, the authors demonstrated that polyspermic fertilization is the main driver of chimerism/mixoploidy in bovine embryos. How common is polyspermy in human IVF (which if I understand correctly, mostly uses ICSI)? How common is polyspermy in human natural conception? Is there any reason to believe that polyspermy rates would differ between human and bovine? The answers to these questions would offer crucial context for how much mixoploidy/chimerism are contributing to pregnancy loss in human IVF and natural conception. If polyspermy is not extremely common, then I don't see how the authors can make claims such as the one that "heterogoneic division" is a major contributor to low human fertility**

Authors: In our dataset of *in vitro* generated bovine embryos presenting with a multipolar zygotic division, polyspermy affected the majority of embryos, leading to genome-wide segregation errors leading and mixoploidy and/or chimerism.

Polyspermy occurs in natural conception. In human, *in vivo* derived pre-implantation embryos have barely been studied, but indirect evidence points to the occurrence of polyspermy. Triploidy, mostly being caused by the fertilization by two sperm cells (Zaragoza *et al.* 2000), is one of the most prevalent causes for early miscarriage. In an unbiased analysis of 85000 pregnancies, triploidy accounted for 1.5% of all pregnancies and 17% of all spontaneous miscarriages (Huskey, 1998)^[1]^. Similar numbers are found in published studies (Levy *et al.* 2014; Wang *et al.* 2017). Obviously, a number of triploid and especially mixoploid/chimeric embryos will not implant. Hence, the numbers could be higher. For human *in vitro* derived pre-implantation embryos, polyspermy following fertilization by IVF occurs at a rate of 6-32% (van der Ven *et al.* 1985; Xia 2013). Hence, dispermic conceptions in human are frequent.

In human *in vitro* derived embryos, polyspermy is avoided by the injection of one sperm cell during ICSI cycles. Data from 34 Belgian reproductive centers show that in 2019, 40 774 cycles were performed of which 15% were fertilized by IVF, 76% with ICSI and 9% with a mixture of IVF and ICSI^[2]^. This means that a large number of cycles (at least more than 6000) is still being fertilized by means of IVF. Following IVF, dispermic conceived embryos are selected against in reproductive laboratories as zygotes with three or more pronuclei (PN) at the time of visual assessment are discarded, as recommended by Eshre Guidelines ^[3]^. That is to avoid the risk of adverse developmental outcomes including abnormal fetal development, molar pregnancy, miscarriage, neonatal death and congenital anomalies. However, selection is 1) not everywhere applied, and 2) not waterproof because PN can develop and appear asynchronous and PN status is assessed at one timepoint only, implying the arrest/development of polyspermic embryos and warranting additional haplotyping-based assessment to avoid transfer embryos containing genome-wide errors.

Polyspermy (and chimerism and mixoploidy) is higher in ART cycles, which would be associated with oocyte incompetency due to hormonal stimulation or *in vitro* maturation and inadequate triggering of the cortical reaction due to frozen semen samples (Xia 2013; Ribas *et al.* 2018).

Although studies applying similar *in vitro* maturation and IVF techniques of bovine oocytes report 15-30% of polyspermy (Wang *et al.* 1997; Ribas *et al.* 2018), which is comparable to polyspermy rates in human *in vitro* produced embryos following IVF, polyspermy rates in bovine and human artificial reproduction systems might differ for two reasons. First, in contrast to human, we cannot select against *in vitro* produced bovine zygotes with multiple PN because of the lipid cytoplasm. Second, we produce embryos starting from immature slaughterhouse-derived oocytes while human *in vitro* produced embryos are derived from more mature oocytes from hormonal treatment of women.

We have included this rationale in our manuscript (Discussion: line 527-561)

Since we do not know the contribution to infertility, we did remove the word ‘major’ but maintain the concept that the phenomenon will contribute to some implantation and developmental failures (Abstract: line 49).

**3) The figures are extremely dense--especially those displaying the raw BAFs. I think that drastically simplifying all of the figures would help expand the potential audience for this work, without sacrificing much in regard to the overall message.**

Authors: we are aware of the fact that the haplarithm plots are difficult to read for those who are not in the field, or have not followed-up on our previous publications. Yet we felt that we had to keep the haplarithm plots inside the main manuscript, because these plots are the only figures presenting the underlying data and support many of the statements provided in the manuscript with evidence. To meet the remark, we have created a new visualization of haplotypes in the main manuscript (Results: line 290-308 and line 459-470; Fig. 4_update.pdf and Fig. 6_update.pdf).

**4) The flow of the text is similarly challenging, as it reads as a very long list of detailed case studies of individual embryos, where hypothetical mechanisms of chimerism/mixoploidy formation are deduced from the haplotype patterns of individual embryos (or small sets of embryos). It would greatly assist readers if the authors would do more to synthesize their findings and provide more concise descriptions of the various error modes. As written, these detailed descriptions of particular mechanisms of error feel somewhat esoteric, written for a narrow specialist audience. It would help, for example, to tally and compare how many embryos fall into the various error categories described. Are some much more common than others? Are some more strongly associated with embryonic arrest? This would help frame the work as more than a series of anecdotes and emphasize the bigger picture. These concerns apply both to the Results and Discussion, which should be made more concise if the hope is to appeal to a broad readership.**

Authors: we have adapted the abstract and the manuscript. Especially, in the abstract we highlighted the essence and reduced the methodological details in the second half. In the main text, we tried to make the text more understandable for a broad public, reduce the amount of detailed information and cut some repetitions.

Specifically, we moved details on some embryos within the section “Embryos with biparental and androgenetic blastomeres” (Results: line 337-352) and within the section “Polyploid embryos (Results: line 392-399) to Additional Figures S2B (Additional files_update_compressed.pdf, Fig. S2B). Similarly, we have moved the paragraph “other embryos”, describing genome-wide profiles and the likely causative events of two complex embryos which could not be categorized together with the other embryos (Results: line 404-421) to Additional Figures S2B (Additional files_update_compressed.pdf, Fig. S2B). In the discussion, we largely removed details on spindle formation in the different embryos, as the core information is covered in the result section and discussion line 578-598 (Discussion: line 598-628). Also, in the discussion, paragraph “Other whole genome segregation errors” was removed, as heterogoneic cell division is the main focus of our manuscript (Discussion: line 641-659).

We have altered the term “ectopic” paternal spindle to “additional” paternal spindle throughout the manuscript, because ectopic might point to an abnormal position of the spindle, which is not necessarily the case and therefore, might be confusing.

We used a multitude of terms for to describe the extrusion of a parental genome into a separate blastomere without a ‘spindle segregation’ such as: ejection, cytoplasmic protrusion etc. We changed it throughout the manuscript to the term ‘extrusion” of a paternal genome.

**5****) The comments that the findings "challenge cell biological dogmas" are overstatements, in my opinion. These are simply errors in the canonical processes of cell division and chromosome segregation.**

Authors: We changed this statement to “These findings… raise questions about the mechanisms triggering zygotic cell division, zygotic spindle formation and genome replication”. (Conclusion: line 715-720).

**Minor comments:**

**Line 54: suggest changing "man" to "humans"**

Authors: this has been changed following the reviewers’ suggestion (Background: line 69).

**Line 354: "****In this study, we demonstrate for the first time that genome-wide mosaicism exists in human preimplantation embryos" is also an overstatement in my opinion. Previous studies, including by these authors, have offered at least indirect evidence of mixoploidy in humans (see references 78-89 of current paper, for example). Is the distinction simply that the authors see it in _preimplantation_ embryos?**

Authors: We thank the reviewer for the critical remark and we agreed to rephrase the sentence (Discussion: line 478-480, line 502-503).

For humans, indeed, earlier cytogenetic studies have pointed to the existence of genome-wide mosaicism in human cleavage-stage embryos (karyotyping and FISH) and blastocysts (FISH). However, in contrast to the haplotype-based methods we apply here, cytogenetic methods used in these former studies suffer from the limitation of not providing genome-wide evidence for the presence of genome copy-numbers (FISH) or parental haplotypes (FISH and karyotyping).

Direct evidence on genome-wide mosaicism comes from our own former studies, which were executed using genome-wide haplotyping methods. However, we have only reported on the existence of chimerism and mixoploidy in cleavage-stage bovine embryos. Taken together with the reports on mixoploidy/chimerism in cleavage-stage primate embryos and the fact that bovine and non-human primate embryogenesis parallel human embryogenesis, including chromosomal error rate (Lequarre et al. 2003; Van Soom et al. 2010; Destouni and Vermeesch 2017; Turner et al. 2019; Suzuki et al. 2021), it could be assumed that similar processes take place in human. Yet, except for reports genome-wide aberrations in PGT-derived biopsies (no genome-wide mosaicism) (McCoy *et al.* 2015; Dimitriadou *et al.* 2017; Destouni *et al.* 2018; Kubicek *et al.* 2019; Tšuiko *et al.* 2021) and one study in which single cell transcriptome suggests the possibility of mixoploidy in one of the analyzed embryos (Starosti *et al.* 2020) no one has ever provided direct genetic evidence of genome-wide mosaicism in human pre-implantation embryos.

For these reasons, we argue that we provide the first direct evidence of mixoploidy and chimerism in human blastocysts and the first direct evidence of chimerism at earlier stages.

**Line 396: "gen" -> "gene"**

Authors: The typo has been changed (Discussion: line 530).

**Additional comments of the authors:**

Authors: We have added three co-authors and their institution for their contribution in the revised version of our manuscript (line 3-5, line 11-15, line 954-959).

Destouni A., Dimitriadou E., Masset H., Debrock S., Melotte C., Van Den Bogaert K., Esteki M. Z., Ding J., Voet T., Denayer E., De Ravel T., Legius E., Meuleman C., Peeraer K., and Vermeesch J. R. (2018). Genome-wide haplotyping embryos developing from 0PN and 1PN zygotes increases transferrable embryos in PGT-M. *Hum. Reprod.* **33**, 2302–2311. doi:10.1093/humrep/dey325

Destouni A., and Vermeesch J. R. (2017). How can zygotes segregate entire parental genomes into distinct blastomeres? The zygote metaphase revisited. *BioEssays* **39**, 1–7. doi:10.1002/bies.201600226

Dimitriadou E., Melotte C., Debrock S., Esteki M. Z., Dierickx K., Voet T., Devriendt K., De Ravel T., Legius E., Peeraer K., Meuleman C., and Vermeesch J. R. (2017). Principles guiding embryo selection following genome-wide haplotyping of preimplantation embryos. *Hum. Reprod.* **32**, 687–697. doi:10.1093/humrep/dex011

Kubicek D., Hornak M., Horak J., Navratil R., Tauwinklova G., Rubes J., and Vesela K. (2019). Incidence and origin of meiotic whole and segmental chromosomal aneuploidies detected by karyomapping. *Reprod. Biomed. Online* **38**, 330–339. doi:10.1016/j.rbmo.2018.11.023

Lequarre A. S., Marchandise J., Moreau B., Massip A., and Donnay I. (2003). Cell cycle duration at the time of maternal zygotic transition for in vitro produced bovine embryos: effect of oxygen tension and transcription inhibition. *Biol. Reprod.* **69**, 1707–1713. doi:10.1095/biolreprod.103.017178

Levy B., Sigurjonsson S., Pettersen B., Maisenbacher M. K., Hall M. P., Demko Z., Lathi R. B., Tao R., Aggarwal V., and Rabinowitz M. (2014). Genomic imbalance in products of conception: single-nucleotide polymorphism chromosomal microarray analysis. *Obstet. Gynecol.* **124**, 202–209. doi:10.1097/AOG.0000000000000325

McCoy R. C., Demko Z. P., Ryan A., Banjevic M., Hill M., Sigurjonsson S., Rabinowitz M., and Petrov D. A. (2015). Evidence of Selection against Complex Mitotic-Origin Aneuploidy during Preimplantation Development. *PLoS Genet.* **11**, e1005601. doi:10.1371/journal.pgen.1005601

Ribas B. N., Missio D., Junior Roman I., Neto N. A., Claro I., dos Santos Brum D., and Leivas F. G. (2018). Superstimulation with eCG prior to ovum pick-up improves follicular development and fertilization rate of cattle oocytes. *Anim. Reprod. Sci.* **195**, 284–290. doi:https://doi.org/10.1016/j.anireprosci.2018.06.006

Schneider I., de Ruijter-Villani M., Hossain M. J., Stout T. A. E., and Ellenberg J. (2021). Dual spindles assemble in bovine zygotes despite the presence of paternal centrosomes. *J. Cell Biol.* **220**,. doi:10.1083/jcb.202010106

Van Soom A., Vandaele L., Peelman L. J., Goossens K., and Fazeli A. (2010). Modeling the interaction of gametes and embryos with the maternal genital tract: from in vivo to in silico. *Theriogenology* **73**, 828–837. doi:10.1016/j.theriogenology.2010.01.001

Starosti M. R., Sosin O. A., and McCo R. C. (2020). Single-cell analysis of human embryos reveals diverse patterns of aneuploidy and mosaicism. *Genome Res.* **30**, 814–826. doi:10.1101/gr.262774.120

Suzuki R., Okada M., Nagai H., Kobayashi J., and Sugimura S. (2021). Morphokinetic analysis of pronuclei using time-lapse cinematography in bovine zygotes. *Theriogenology*. doi:https://doi.org/10.1016/j.theriogenology.2021.02.021

Tšuiko O., Vanneste M., Melotte C., Ding J., Debrock S., Masset H., Peters M., Salumets A., De Leener A., Pirard C., Kluyskens C., Hostens K., van de Vijver A., Peeraer K., Denayer E., Vermeesch J. R., and Dimitriadou E. (2021). Haplotyping-based preimplantation genetic testing reveals parent-of-origin specific mechanisms of aneuploidy formation. *npj Genomic Med.* **6**, 81. doi:10.1038/s41525-021-00246-0

Turner K. J., Silvestri G., Black D. H., Dobson G., Smith C., Handyside A. H., Sinclair K. D., and Griffin D. K. (2019). Karyomapping for simultaneous genomic evaluation and aneuploidy screening of preimplantation bovine embryos: The first live-born calves. *Theriogenology* **125**, 249–258. doi:10.1016/j.theriogenology.2018.11.014

van der Ven H. H., Al-Hasani S., Diedrich K., Hamerich U., Lehmann F., and Krebs D. (1985). Polyspermy in in vitro fertilization of human oocytes: frequency and possible causes. *Ann. N. Y. Acad. Sci.* **442**, 88–95. doi:10.1111/j.1749-6632.1985.tb37508.x

Wang Y., Cheng Q., Meng L., Luo C., Hu H., Zhang J., Cheng J., Xu T., Jiang T., Liang D., Hu P., and Xu Z. (2017). Clinical application of SNP array analysis in first-trimester pregnancy loss: a prospective study. *Clin. Genet.* **91**, 849–858. doi:10.1111/cge.12926

Wang W., Hosoe M., Li R., and Shioya Y. (1997). Development of the competence of bovine oocytes to release cortical granules and block polyspermy after meiotic maturation. *Dev. Growth Differ.* **39**, 607–615. doi:10.1046/j.1440-169x.1997.t01-4-00008.x

Xia P. (2013). Biology of Polyspermy in IVF and its Clinical Indication. *Curr. Obstet. Gynecol. Rep.* **2**, 226–231. doi:10.1007/s13669-013-0059-2

Zaragoza M. V, Surti U., Redline R. W., Millie E., Chakravarti A., and Hassold T. J. (2000). Parental origin and phenotype of triploidy in spontaneous abortions: predominance of diandry and association with the partial hydatidiform mole. *Am. J. Hum. Genet.* **66**, 1807–1820. doi:10.1086/302951

**Second round of review**

**Reviewer 1**

De Coster, Vermeesch and colleagues hand in a revised version of their manuscript “Parental genomes segregate into distinct blastomeres during multipolar zygotic divisions leading to mixoploid and chimeric blastocysts”.

While the original manuscript was already in good shape, this new version comes out with some additional, very worthwhile experiments that further strengthen the arguments and conclusions.

In particular, directly visualizing aberrant spindles as the obvious structural basis for embryonic genome aberrations and mixoploidy make a very nice add-on to the previous experiments. The imaging came out beautiful and impressively demonstrates what was suggested earlier. I also recapitulate the technical obstacles that preclude pre-selection of embryos to pinpoint 2N situations. What is now added to the discission still reasonably addresses this point. Finally, I also appreciate the extensive re-writing of the manuscript including clarification of experimental “systems” or situations, and having concisely restructured the abstract of the manuscript.

Taken together, besides some few spelling errors that will be corrected, the current version of the manuscript will certainly make an excellent contribution to Genome Biology and should be published essentially in its present form.

**Reviewer 2**

I appreciate the authors' careful consideration of my comments, as well as those of the other reviewer. I am broadly satisfied with these revisions. The live imaging of multipolar divisions in bovine embryos is a nice addition. While the figures are still very dense, I think they are improved, and I appreciate the authors' motivation for transparency. The Discussion is also more concise and readable, and some of the overstatements that I noted before have been appropriately moderated. This is an important contribution to the literature on the origins of complex chromosome abnormalities in mammalian embryos.

1. http://www.biologia.uniba.it/Materiale%20extra%20Rocchi/07-Anomalie%20cromosomiche.ppt
2. <https://www.belrap.be/Documents/Reports/Global/BelrapSummaryReport2019.pdf>
3. Eshre Guideline Group on Good Practice in IVF Labs et al., 2016
